# NIST Interlaboratory Study on Glycosylation Analysis of Monoclonal Antibodies: Comparison of Results from Diverse Analytical Methods*[Fn FN2]

**DOI:** 10.1074/mcp.RA119.001677

**Published:** 2019-10-07

**Authors:** Maria Lorna A. De Leoz, David L. Duewer, Adam Fung, Lily Liu, Hoi Kei Yau, Oscar Potter, Gregory O. Staples, Kenichiro Furuki, Ruth Frenkel, Yunli Hu, Zoran Sosic, Peiqing Zhang, Friedrich Altmann, Clemens Gru«nwald-Grube, Chun Shao, Joseph Zaia, Waltraud Evers, Stuart Pengelley, Detlev Suckau, Anja Wiechmann, Anja Resemann, Wolfgang Jabs, Alain Beck, John W. Froehlich, Chuncui Huang, Yan Li, Yaming Liu, Shiwei Sun, Yaojun Wang, Youngsuk Seo, Hyun Joo An, Niels-Christian Reichardt, Juan Echevarria Ruiz, Stephanie Archer-Hartmann, Parastoo Azadi, Len Bell, Zsuzsanna Lakos, Yanming An, John F. Cipollo, Maja Pucic-Bakovic, Jerko Štambuk, Gordan Lauc, Xu Li, Peng George Wang, Andreas Bock, René Hennig, Erdmann Rapp, Marybeth Creskey, Terry D. Cyr, Miyako Nakano, Taiki Sugiyama, Pui-King Amy Leung, Paweł Link-Lenczowski, Jolanta Jaworek, Shuang Yang, Hui Zhang, Tim Kelly, Song Klapoetke, Rui Cao, Jin Young Kim, Hyun Kyoung Lee, Ju Yeon Lee, Jong Shin Yoo, Sa-Rang Kim, Soo-Kyung Suh, Noortje de Haan, David Falck, Guinevere S. M. Lageveen-Kammeijer, Manfred Wuhrer, Robert J. Emery, Radoslaw P. Kozak, Li Phing Liew, Louise Royle, Paulina A. Urbanowicz, Nicolle H. Packer, Xiaomin Song, Arun Everest-Dass, Erika Lattová, Samanta Cajic, Kathirvel Alagesan, Daniel Kolarich, Toyin Kasali, Viv Lindo, Yuetian Chen, Kudrat Goswami, Brian Gau, Ravi Amunugama, Richard Jones, Corné J. M. Stroop, Koichi Kato, Hirokazu Yagi, Sachiko Kondo, C. T. Yuen, Akira Harazono, Xiaofeng Shi, Paula E. Magnelli, Brian T. Kasper, Lara Mahal, David J. Harvey, Roisin O'Flaherty, Pauline M. Rudd, Radka Saldova, Elizabeth S. Hecht, David C. Muddiman, Jichao Kang, Prachi Bhoskar, Daniele Menard, Andrew Saati, Christine Merle, Steven Mast, Sam Tep, Jennie Truong, Takashi Nishikaze, Sadanori Sekiya, Aaron Shafer, Sohei Funaoka, Masaaki Toyoda, Peter de Vreugd, Cassie Caron, Pralima Pradhan, Niclas Chiang Tan, Yehia Mechref, Sachin Patil, Jeffrey S. Rohrer, Ranjan Chakrabarti, Disha Dadke, Mohammedazam Lahori, Chunxia Zou, Christopher Cairo, Béla Reiz, Randy M. Whittal, Carlito B. Lebrilla, Lauren Wu, Andras Guttman, Marton Szigeti, Benjamin G. Kremkow, Kelvin H. Lee, Carina Sihlbom, Barbara Adamczyk, Chunsheng Jin, Niclas G. Karlsson, Jessica Örnros, Göran Larson, Jonas Nilsson, Bernd Meyer, Alena Wiegandt, Emy Komatsu, Helene Perreault, Edward D. Bodnar, Nassur Said, Yannis-Nicolas Francois, Emmanuelle Leize-Wagner, Sandra Maier, Anne Zeck, Albert J. R. Heck, Yang Yang, Rob Haselberg, Ying Qing Yu, William Alley, Joseph W. Leone, Hua Yuan, Stephen E. Stein

**Affiliations:** 1 Mass Spectrometry Data Center, Biomolecular Measurement Division, Material Measurement Laboratory, National Institute of Standards and Technology, 100 Bureau Drive Gaithersburg, Maryland 20899; 2 Chemical Sciences Division, Material Measurement Laboratory, National Institute of Standards and Technology, 100 Bureau Drive Gaithersburg, Maryland 20899; 3 Analytical Development, Agensys, Inc., 1800 Steward Street Santa Monica, California 90404; 4 Agilent Technologies, Inc., 5301 Stevens Creek Blvd Santa Clara, California 95051; 5 Astellas Pharma, 5–2-3 Tokodai, Tsukiba, Ibaraki, 300–2698, Japan; 6 Analytical Development, Biogen, 14 Cambridge Center Cambridge, Massachusetts 02142; 7 Bioprocessing Technology Institute, 20 Biopolis Way, Level 3 Singapore 138668; 8 Department of Chemistry, University of Natural Resources and Life Science, Vienna (BOKU), Muthgasse 18 1190 Wien, Austria; 9 Center for Biomedical Mass Spectrometry, Boston University School of Medicine, 670 Albany Street Boston, Massachusetts 02118; 10 Bruker Daltonik GmbH, Fahrenheitstr. 4, 28359 Bremen, Germany; 11 Department of Life Sciences & Technology, Beuth Hochschule für Technik Berlin, Seestraβe 64, 13347 Berlin, Germany; 12 Centre d'Immunologie Pierre Fabre, 5 Avenue Napoléon III, BP 60497, 74164 St Julien-en-Genevois, France; 13 Department of Urology, Boston Children's Hospital, 300 Longwood Avenue Boston Massachusetts 02115; 14 Institute of Biophysics, Chinese Academy of Sciences, 15 Da Tun Road, Chaoyang District, Beijing 100101 China; 15 Key Lab of Intelligent Information Processing, Institute of Computing Technology, Chinese Academy of Sciences, 15 Da Tun Road, Chaoyang District, Beijing 100101 China; 16 Graduate School of Analytical Science and Technology, Chungnam National University, Gung-dong 220, Yuseong-Gu, Daejeon 305–764, Korea (South); 17 CICbiomaGUNE, Paseo Miramon 182, 20009 San Sebastian, Spain; 18 Analytical Services, Complex Carbohydrate Research Center, University of Georgia, 315 Riverbend Road Athens, Georgia 30602; 19 BioCMC Solutions (Large Molecules), Covance Laboratories Limited, Otley Road, Harrogate, North Yorks HG3 1PY, United Kingdom; 20 Biochemistry Method Development & Validation, Eurofins Lancaster Laboratories, Inc., 2425 New Holland Pike Lancaster, Pennsylvania 17601; 21 Center for Biologics Evaluation and Research, Food and Drug Administration, 10903 New Hampshire Avenue, Silver Spring, Maryland 20993; 22 Glycoscience Research Laboratory, Genos, Borongajska cesta 83h, 10 000 Zagreb, Croatia; 23 Faculty of Pharmacy and Biochemistry, University of Zagreb, A. Kovačića 1, 10 000 Zagreb, Croatia; 24 Department of Chemistry, Georgia State University, 100 Piedmont Avenue, Atlanta, Georgia 30303; 25 glyXera GmbH, Brenneckestrasse 20 * ZENIT / 39120 Magdeburg, Germany; 26 Health Products and Foods Branch, Health Canada, AL 2201E, 251 Sir Frederick Banting Driveway, Ottawa, Ontario, K1A 0K9 Canada; 27 Graduate School of Advanced Sciences of Matter, Hiroshima University, 1-3-1 Kagamiyama Higashi-Hiroshima 739–8530 Japan; 28 ImmunoGen, 830 Winter Street, Waltham, Massachusetts 02451; 29 Department of Medical Physiology, Jagiellonian University Medical College, ul. Michalowskiego 12, 31–126 Krakow, Poland; 30 Department of Pathology, Johns Hopkins University, 400 N. Broadway Street Baltimore, Maryland 21287; 31 Mass Spec Core Facility, KBI Biopharma, 1101 Hamlin Road Durham, North Carolina 27704; 32 Division of Mass Spectrometry, Korea Basic Science Institute, 162 YeonGuDanji-Ro, Ochang-eup, Cheongwon-gu, Cheongju Chungbuk, 363–883 Korea (South); 33 Advanced Therapy Products Research Division, Korea National Institute of Food and Drug Safety, 187 Osongsaengmyeong 2-ro Osong-eup, Heungdeok-gu, Cheongju-si, Chungcheongbuk-do, 363–700, Korea (South); 34 Center for Proteomics and Metabolomics, Leiden University Medical Center, P.O. Box 9600, 2300 RC Leiden, The Netherlands; 35 Ludger Limited, Culham Science Centre, Abingdon, Oxfordshire, OX14 3EB, United Kingdom; 36 Biomolecular Discovery and Design Research Centre and ARC Centre of Excellence for Nanoscale BioPhotonics (CNBP), Macquarie University, North Ryde, Australia; 37 Proteomics, Central European Institute for Technology, Masaryk University, Kamenice 5, A26, 625 00 BRNO, Czech Republic; 38 Max Planck Institute for Dynamics of Complex Technical Systems, Sandtorstrasse 1, 39106 Magdeburg, Germany; 39 Department of Biomolecular Sciences, Max Planck Institute of Colloids and Interfaces, 14424 Potsdam, Germany; 40 AstraZeneca, Granta Park, Cambridgeshire, CB21 6GH United Kingdom; 41 Merck, 2015 Galloping Hill Rd, Kenilworth, New Jersey 07033; 42 Analytical R&D, MilliporeSigma, 2909 Laclede Ave. St. Louis, Missouri 63103; 43 MS Bioworks, LLC, 3950 Varsity Drive Ann Arbor, Michigan 48108; 44 MSD, Molenstraat 110, 5342 CC Oss, The Netherlands; 45 Exploratory Research Center on Life and Living Systems (ExCELLS), National Institutes of Natural Sciences, 5–1 Higashiyama, Myodaiji, Okazaki 444–8787 Japan; 46 Graduate School of Pharmaceutical Sciences, Nagoya City University, 3–1 Tanabe-dori, Mizuhoku, Nagoya 467–8603 Japan; 47 Medical & Biological Laboratories Co., Ltd, 2–22-8 Chikusa, Chikusa-ku, Nagoya 464–0858 Japan; 48 National Institute for Biological Standards and Control, Blanche Lane, South Mimms, Potters Bar, Hertfordshire EN6 3QG United Kingdom; 49 Division of Biological Chemistry & Biologicals, National Institute of Health Sciences, 1–18-1 Kamiyoga, Setagaya-ku, Tokyo 158–8501 Japan; 50 New England Biolabs, Inc., 240 County Road, Ipswich, Massachusetts 01938; 51 New York University, 100 Washington Square East New York City, New York 10003; 52 Target Discovery Institute, Nuffield Department of Medicine, University of Oxford, Roosevelt Drive, Oxford, OX3 7FZ, United Kingdom; 53 GlycoScience Group, The National Institute for Bioprocessing Research and Training, Fosters Avenue, Mount Merrion, Blackrock, Co. Dublin, Ireland; 54 Department of Chemistry, North Carolina State University, 2620 Yarborough Drive Raleigh, North Carolina 27695; 55 Pantheon, 201 College Road East Princeton, New Jersey 08540; 56 Pfizer Inc., 1 Burtt Road Andover, Massachusetts 01810; 57 Proteodynamics, ZI La Varenne 20–22 rue Henri et Gilberte Goudier 63200 RIOM, France; 58 ProZyme, Inc., 3832 Bay Center Place Hayward, California 94545; 59 Koichi Tanaka Mass Spectrometry Research Laboratory, Shimadzu Corporation, 1 Nishinokyo Kuwabara-cho Nakagyo-ku, Kyoto, 604 8511 Japan; 60 Children's GMP LLC, St. Jude Children's Research Hospital, 262 Danny Thomas Place Memphis, Tennessee 38105; 61 Sumitomo Bakelite Co., Ltd., 1–5 Muromati 1-Chome, Nishiku, Kobe, 651–2241 Japan; 62 Synthon Biopharmaceuticals, Microweg 22 P.O. Box 7071, 6503 GN Nijmegen, The Netherlands; 63 Takeda Pharmaceuticals International Co., 40 Landsdowne Street Cambridge, Massachusetts 02139; 64 Department of Chemistry and Biochemistry, Texas Tech University, 2500 Broadway, Lubbock, Texas 79409; 65 Thermo Fisher Scientific, 1214 Oakmead Parkway Sunnyvale, California 94085; 66 United States Pharmacopeia India Pvt. Ltd. IKP Knowledge Park, Genome Valley, Shamirpet, Turkapally Village, Medchal District, Hyderabad 500 101 Telangana, India; 67 Alberta Glycomics Centre, University of Alberta, Edmonton, Alberta T6G 2G2 Canada; 68 Department of Chemistry, University of Alberta, Edmonton, Alberta T6G 2G2 Canada; 69 Department of Chemistry, University of California, One Shields Ave, Davis, California 95616; 70 Horváth Csaba Memorial Laboratory for Bioseparation Sciences, Research Center for Molecular Medicine, Doctoral School of Molecular Medicine, Faculty of Medicine, University of Debrecen, Debrecen, Egyetem ter 1, Hungary; 71 Translational Glycomics Research Group, Research Institute of Biomolecular and Chemical Engineering, University of Pannonia, Veszprem, Egyetem ut 10, Hungary; 72 Delaware Biotechnology Institute, University of Delaware, 15 Innovation Way Newark, Delaware 19711; 73 Proteomics Core Facility, University of Gothenburg, Medicinaregatan 1G SE 41390 Gothenburg, Sweden; 74 Department of Medical Biochemistry and Cell Biology, University of Gothenburg, Institute of Biomedicine, Sahlgrenska Academy, Medicinaregatan 9A, Box 440, 405 30, Gothenburg, Sweden; 75 Department of Clinical Chemistry and Transfusion Medicine, Sahlgrenska Academy at the University of Gothenburg, Bruna Straket 16, 41345 Gothenburg, Sweden; 76 Department of Chemistry, University of Hamburg, Martin Luther King Pl. 6 20146 Hamburg, Germany; 77 Department of Chemistry, University of Manitoba, 144 Dysart Road, Winnipeg, Manitoba, Canada R3T 2N2; 78 Laboratory of Mass Spectrometry of Interactions and Systems, University of Strasbourg, UMR Unistra-CNRS 7140, France; 79 Natural and Medical Sciences Institute, University of Tübingen, Markwiesenstraβe 55, 72770 Reutlingen, Germany; 80 Bijvoet Center for Biomolecular Research and Utrecht Institute for Pharmaceutical Sciences, Utrecht University, Padualaan 8, 3584 CH Utrecht, The Netherlands; 81 Division of Bioanalytical Chemistry, Amsterdam Institute for Molecules, Medicines and Systems, Vrije Universiteit Amsterdam, de Boelelaan 1085, 1081 HV Amsterdam, The Netherlands; 82 Department of Chemistry, Waters Corporation, 34 Maple Street Milford, Massachusetts 01757; 83 Zoetis, 333 Portage St. Kalamazoo, Michigan 49007

**Keywords:** Glycomics, mass spectrometry, fluorescence, glycosylation, glycoproteins, glycan, glycopeptide, interlaboratory study, NISTmAb, reference antibody

## Abstract

Glycosylation is a topic of intense current interest in the development of biopharmaceuticals because it is related to drug safety and efficacy. This work describes results of an interlaboratory study on the glycosylation of the Primary Sample (PS) of NISTmAb, a monoclonal antibody reference material. Seventy-six laboratories from industry, university, research, government, and hospital sectors in Europe, North America, Asia, and Australia submitted a total of 103 reports on glycan distributions. The principal objective of this study was to report and compare results for the full range of analytical methods presently used in the glycosylation analysis of mAbs. Therefore, participation was unrestricted, with laboratories choosing their own measurement techniques. Protein glycosylation was determined in various ways, including at the level of intact mAb, protein fragments, glycopeptides, or released glycans, using a wide variety of methods for derivatization, separation, identification, and quantification. Consequently, the diversity of results was enormous, with the number of glycan compositions identified by each laboratory ranging from 4 to 48. In total, one hundred sixteen glycan compositions were reported, of which 57 compositions could be assigned consensus abundance values. These consensus medians provide community-derived values for NISTmAb PS. Agreement with the consensus medians did not depend on the specific method or laboratory type. The study provides a view of the current state-of-the-art for biologic glycosylation measurement and suggests a clear need for harmonization of glycosylation analysis methods.

## Abstract (precis)

A broad-based interlaboratory study of glycosylation profiles of a reference and modified IgG antibody involving 103 reports from 76 laboratories.

## Graphical Abstract

**Figure:**
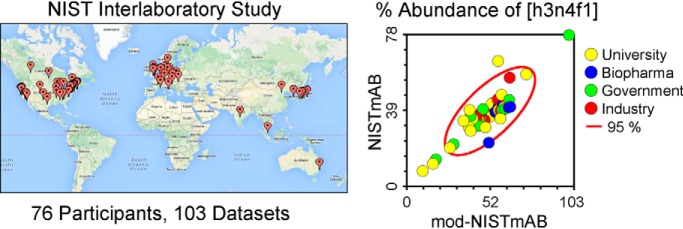

**Highlights**

- A broad-based interlaboratory study of the glycosylation of a reference antibody: NISTmAb.
- 103 reports were received from 76 diverse laboratories worldwide.
- Analysis involved two samples, the NISTmAb and an enzymatically modified sample, enabling within-lab separation of random and systematic errors using the “Youden two-sample” method.
- Consensus values were derived and similar performance across all experimental methods was noted.

Biologics have recently emerged as critically important drugs from health and economic perspectives. Two-thirds of approved biologics are glycoproteins, *i.e.* proteins containing glycans as post-translational modification. Alteration in glycosylation may impact the safety and efficacy of the drug, including its clearance rates, effector functions, folding, immunogenicity, solubility, and biological activity. In addition to glycomic profiling of new drug candidates, analysis of glycoforms is essential for monitoring production batches of established drugs and comparing biosimilars and biobetters to originator drugs.

This report describes results of a broad interlaboratory study designed to determine both the level of variability in current measurement methods as well as to support consensus measurement values for a reference material. Participation was open to all laboratories, regardless of experience or preferred analytical method. Because specific methods selected by participating laboratories varied greatly, as did their degree of expertise, this study was not designed to determine “best” methods, but to provide a “snapshot” of the currently used methods for biologic glycosylation measurement. Unfortunately, this diversity in experience and objective prevented a deeper analysis of the variability of results, with some highly experienced labs using well-developed standard operating procedures, and with others using novel approaches or exploiting their unique capabilities. The study rationale and design are presented in detail in supplementary Discussion S1.

Glycosylation analysis is inherently challenging because, unlike amino acids in proteins which are encoded by the genome, sequential addition of monosaccharide residues is not template-driven. It is rather dictated by competing enzymatic activities, leading to heterogeneity. Even at the same site of glycosylation, diverse glycans with different linkages, number of antenna, and monosaccharide compositions are possible, giving rise to challenges in separation (chromatography) and isomerization (mass spectrometry).

A common glycosylation in mAbs is *N*-glycosylation where the glycans are linked to the nitrogen of the Asn residue of the protein with a consensus sequence Asn-X-Ser/Thr or, more rarely, Asn-X-Cys where X is any amino acid except proline. Moreover, *N*-glycans have a common five-membered trimannosyl chitobiose core, Manα1–6(Manα1–3)Manβ1- 4GlcNAcβ1–4GlcNAcβ1-Asn-X-Ser/Thr. The highly complex nature of *N*-glycosylation analysis has given rise to a proliferation of different methods (1–14). Currently, *N*-glycosylation is examined at the level of intact proteins, protein fragments, peptides, glycans, or monosaccharides. Analytes are then analyzed by mass spectrometry (MS)^1^ (1); liquid chromatography (LC) with fluorescence detection (FD)(2) and/or MS detection; capillary electrophoresis (CE) with MS detection (3); CE-laser-induced fluorescence detection (CE-LIF); high performance anion exchange chromatography with pulsed amperometric detection (HPAEC-PAD); nuclear magnetic resonance (NMR) spectroscopy; or a combination of these techniques (4).

One popular approach is the release of glycans where *N*-glycans are cleaved from proteins using Peptide-N-Glycosidase F (PNGase F), which hydrolyzes the side-chain amide group of the glycosylated asparagine. Before analysis, glycans may be subjected to permethylation, reduction, or fluorophore labeling to increase sensitivity and specificity. Structure elucidation and isomer separation is possible using the glycan-release approach, but it lacks information on the site of glycosylation because analysis is performed after the glycans are cleaved from the protein.

Analysis of glycopeptides can provide glycosylation site information along with glycan compositions. In this approach, mAbs are digested with proteases such as trypsin (and less commonly used enzymes such as chymotrypsin, LysC, LysN, AspN, GluC, or ArgC) to produce peptides and glycopeptides that are then typically analyzed using MALDI-MS and LC-MS(/MS) methods (and less commonly CE-MS(/MS) methods (15)). The peptide attached to the glycoform gives information on the site of glycosylation. Potential disadvantages include challenges in differentiating isomers and suppression of glycopeptide ions because of peptide ions at the precursor (MS1) level. The latter could be alleviated by (two-dimensional) LC or enrichment methods (16).

Middle-down and top-down approaches characterize the glycosylation by analyzing protein fragments and intact proteins, respectively. In the middle-down approach, mAbs are treated with immunoglobulin G-degrading enzyme (IdeS), an endopeptidase that cleaves heavy chains below the hinge region, resulting in antigen-binding (Fab) and crystallizable (Fc) fragments. These large fragments are then usually analyzed by MS. Protein fragments have a lower molecular mass than the intact protein and could be better resolved in MS compared with the analysis of intact mAbs in the top-down approach. Compared with other techniques, the top-down approach provides the advantage that little-to-no sample preparation steps are needed before the analysis. Typically, only desalting of the intact mAb is necessary, which is normally performed with a desalting column (*e.g.* C4, C8) followed by the analysis with MS. However, because top-down and middle-down analyses often result in higher masses, fewer glycan compositions can be distinguished because of lack of resolution compared with other MS-based methods.

The diversity of these methods presents a major challenge in the interpretation of *N*-glycosylation measurements. Unfortunately, only a few multi-laboratory studies have been reported assessing the performance of the different approaches (17–21). In two studies by the Human Proteome Organization (HUPO), relative abundances of *N*-glycans (in transferrin and IgG) (17) and O-glycans (in IgA1) (18) were analyzed by 20 and 15 laboratories, respectively. They observed that MS-based methods are efficient in identifying and quantifying glycans. However, there were no participants from biopharmaceutical companies.

Here we present the design and results of our interlaboratory study of two materials: primary sample (PS) 8670, commonly referred to as NISTmAb (22), and mod-NISTmAb, a material derived from PS 8670 by modification with galactosidase. PS 8670 is the in-house standard for NIST Reference Material 8671 (23). The rationale for the use of these samples is presented in supplementary Discussion S2. This report is based on 103 reports submitted by 76 laboratories worldwide. It builds on the NIST internal report (NISTIR) 8186 (24).

This interlaboratory study had two goals. The first goal was to determine measurement variability in identifying and quantifying *N*-glycosylation in monoclonal antibodies across laboratories in the glycomics and glycoproteomics community, including laboratories form biopharmaceutical companies and universities. The second goal was to aid in determining community-based consensus medians for the glycosylation of the PS. The community's consensus values for NISTmAb PS 8670 glycosylation, robustly estimated as medians, represent an unparalleled diversity of approaches applied to the same material and serve as a seminal baseline for comparing glycoanalytical strategies.

Finally, we note two quite different levels of identification - by composition and by structure. Compositions are determined by high mass accuracy mass spectrometry, whereas confident isomer identification often requires reference materials or chromatographic retention matching.

## EXPERIMENTAL PROCEDURES

### 

#### 

##### Monoclonal Antibody Sample Preparation

Two materials were used in the study, (1 the Primary sample (PS) for NIST Reference Material 8671, NISTmAb, Humanized IgG1κ Monoclonal Antibody produced in NS0 cells, and (2 a material derived from the PS by treatment with galactosidase, termed “mod-NISTmAb.”

NISTmAb was obtained as a bulk substance prepared using mammalian cell culture and downstream processing. It has one *N*-glycosylation site at the Fc region of the antibody. mod-NISTmAb was prepared by subjecting a portion of NISTmAb to β-1,4-galactosidase (New England Biolabs, Ipswich, MA) and then adding the resulting solution back to the original NISTmAb (30:70 by mass).

##### Study Execution

The study was conducted in two stages: Stage 1 involved nine selected laboratories who volunteered to assist in final study design; Stage 2 was widely advertised and open to all laboratories. Two samples were shipped to laboratories on June 2015 and August-September 2015 for Stage 1 and Stage 2, respectively. Laboratories received three vials consisting of two blinded monoclonal antibody samples and one buffer solution in 1.0 ml screw-top tubes (Matrix™ Thermo Fisher Scientific, #3740) as follows:

- Sample A: white label, frozen liquid, 0.4 mg, 100 mg/ml mAb
- Sample B: blue label, frozen liquid, 0.4 mg, 100 mg/ml mAb
- Buffer: yellow label, frozen liquid, 1 ml, 25 mmol/L l-Histidine, pH 6.0

Laboratories were informed that both samples are humanized IgG1k expressed in murine suspension culture and that the samples are “drug-like substances” not for human use. The buffer solution was provided as a diluent.

Participants used their method of choice to determine the glycan content in the two samples. Participants were requested to provide measurement results using NIST-provided data and method reporting templates (24) by July 30, 2015 (Stage 1) and November 6, 2015 (Stage 2). Some laboratories submitted more than one report; each report was assigned a confidential laboratory number (and was treated as a separate laboratory). Participants could enter other glycans or methods in the template; no other post-translational modifications, *e.g.* lysine glycation, could be reported.

Data were analyzed as reported, *i.e.* no normalization, using a variety of robust statistical analysis techniques to assess measurement reproducibility and to characterize glycan distributions. Results were compiled and evaluated for determination of community's consensus medians, within-laboratory precision, and concordance within the laboratories. A technical summary (24) of reported and derived values from all laboratories, a table of all identified glycans, and an individualized graphical analysis of their performance for the exercise were sent to the participating laboratories on June 2, 2017.

##### Shipping

Package shipped to each laboratory consisted of three vials (Sample A, Sample B, and l-Histidine buffer solution) and a welcome packet (24). The three vials were stowed in a rolled, self-sealing bubble wrap bag and placed in an insulated box filled with dry ice. The welcome packet consisted of a cover letter; instructions; packing list/shipment receipt confirmation form; and data, method, and comment reporting sheets. These documents were enclosed in a waterproof sleeve and placed at the top of the shipping box, between the cardboard covering and the foam insulation. A soft copy of the welcome packet was emailed to participants as one spreadsheet workbook with multiple worksheets. Participants were requested to return the filled shipment receipt confirmation form as soon as they received the shipped package.

##### Analysis Methods

Each laboratory was asked to perform glycosylation analysis of the two samples in triplicate using their own method(s), as summarized in Table I. Briefly, glycans were cleaved by incubating mAbs with PNGase F (74 reports), trypsin/PNGase F (1 report), and Pepsin/PNGase A (1 report). Cleaved glycans were derivatized using fluorescent (54 reports) or non-fluorescent (22 reports) methods. Next, glycans were separated with chromatography (CE (5 reports), HILIC (46 reports), IC (1 report), PGC (6 reports), RP (6 reports)) or without chromatography (12 reports), and then identified by various analytical methods.

Glycopeptides were cleaved from mAbs using trypsin (21 reports). Cleaved glycopeptides were left underivatized (18 reports) or subjected to dimethylamidation (1 report), Ludger V-tag (1 report) or reduction (1 report). Glycopeptide separation was performed using RP (17 reports), HILIC (1 report) or CE (1 report) chromatography. MS (20 reports) or FD (1 report) was used for analysis.

To obtain protein fragments, Ides (2 reports) or Endo-S (1 report) enzymes were added to mAbs. No derivatization was performed before chromatography using CE (1 report), RP (1 report), or SEC (1 report). Analysis was performed using LC-MS. Intact mAbs were analyzed with (1 report) or without PNGase F (1 report) and with (1 report) or without RP (1 report) chromatography. Intact mAbs analysis was performed using MS.

Laboratories recorded in a provided template a) methods used and b) percent abundances of glycans. Laboratories were asked to create separate reports for each method of analysis. If a value obtained was below their limit of detection or quantification, participants were asked to indicate this result as “ND” (not detected) or “NQ” (not quantified), respectively.

### Describing Glycans in the Data Reporting Template

#### 

##### Naming Conventions

Currently, there is no standard way of naming *N*-glycans. Common names using the G0F and Oxford naming conventions were used. In cases where, to the authors' knowledge, there is no existing name for a glycan, every attempt was made to derive it from naming conventions, summarized below.

##### Common Name

1. All *N*-glycans have two core GlcNAcs;
2. High mannose glycans are named ManX where X is the number of mannoses after the two core GlcNAcs;
3. F is fucose, number after F indicates number of fucoses. No number indicates the presence of core fucose only;
4. Gx is galactose and x is the number of terminal Gal connected to two GlcNAcs, G0 is a biantennary complex glycan with two terminal GlcNAcs, G1 is a biantennary complex glycan with two GlcNAcs and one terminal Gal, G2 is a biantennary complex glycan with two GlcNAcs and two terminal Gal;
5. Gx-yN means y GlcNAc is missing, *e.g.* G1-N is biantennary complex glycan with one terminal GlcNAc and one terminal Gal;
6. S (NeuAc) or (NeuGc) is sialic acid. (NeuAc) or (NeuGc) indicates type of sialic acid;
7. Number in parenthesis indicates linkage: F(6) or S(6) means a α1–6-linked core fucose or α2,6-linked sialic acid, respectively;
8. Number in square brackets is the location of residue, *e.g.* (3) or (6) indicates that the residue is in the α1,3 or α1,6 mannose arm, respectively; and
9. xaGal is α-1,3-linked galactose, x is the number of residues;

##### Oxford Name

1. All *N*-glycans have two core GlcNAcs;
2. F at the start of the abbreviation indicates a core fucose, (6) after the F indicates that the fucose is α1–6 linked to the inner GlcNAc;
3. Mx, number (x) of mannose on core GlcNAcs;
4. Ax, number of antenna (GlcNAc) on trimannosyl core; A2, biantennary with GlcNAcs as β1–2 linked; A3, triantennary with a GlcNAc linked β1–2 to both mannose and the third GlcNAc linked β1–4 to the α1–3 linked mannose; A3′, triantennary with a GlcNAc linked β1–2 to both mannose and the third GlcNAc linked β1–6 to the α1–6 linked mannose; A4, GlcNAcs linked as A3 with additional GlcNAc β1–6 linked to α1–6 mannose;
5. B, bisecting GlcNAc linked β1–4 to α1–3 mannose;
6. Gx, number (x) of linked galactose on antenna, (4) or (3) after the G indicates that the Gal is β1–4 or β1–3 linked; [3]G1 and [6]G1 indicates that the galactose is on the antenna of the α1–3 or α1–6 mannose;
7. Gax, number (x) of linked alpha galactose on antenna; and
8. Sx, number (x) of sialic acids linked to galactose; the numbers 3 or 6 in parentheses after S indicate whether the sialic acid is in an α2–3 or α2–6 linkage.

##### Monosaccharide Composition

The monosaccharide composition is inside a square bracket. Small letters were used to avoid confusion with elements (hydrogen, nitrogen, fluorine, etc.): h = hexose, *n* = *N*-acetylhexosamine, f = deoxyhexose (*e.g.* fucose), a = NeuAc, g = NeuGc. Number after the letter denotes the number of residues. For example, [h6n4f1a1] has 6 hexoses, 4 *N*-acetylhexosamine, 1 fucose, 1 NeuAc. For sulfonated glycans, S = sulfur.

##### Structure Conventions

Two structure notations were used: the Consortium for Functional Glycomics (CFG) (25) and the Oxford Glycobiology Institute (UOXF) notations. Glycoworkbench 2.1 (Eurocarb) (26) was used to draw the structures. The differences are in the monosaccharide residue representations (for example, NeuAc is purple diamond in CFG but purple star in UOXF notations) and linkage notations (angle represents linkage in UOXF). A revised CFG format has been introduced but it was not used in this reporting template.

##### Calculations for Derived Attributes of NISTmAb

Calculations for the glycan attributes were estimated from the median results and based on an earlier reported method (27):

- Galactosylation = Sum of (% abundance)×(galactosylation factor) for all glycans with terminal galactose where the factor is the fraction of antennae that are galactosylated. For example, the galactosylation factor of G0F is 0, G1F is 0.5, G2F is 1.
  - α Galactosylation = As above but for α galactosylation only
- Sialylation = Sum of (% abundance) × (sialylation factor) for all glycans with NeuAc or NeuGc sialic acid where the factor is the fraction of antanaee that are sialylated.
  - NeuAc sialylation = As above, but for NeuAc only
  - NeuGc sialylation = As above, but for NeuGc only
- Fucosylation = Sum of (% abundance) for all glycans with fucose residues.
  - Core fucosylation only = Sum of (% abundance) for all glycans with 1 fucose. This calculation assumes that the first fucose is always a core fucose.
  - Difucosylation = Sum of (% abundance) for all glycans with 2 fucose residues.
- Bisecting GlcNAc = Sum of (% abundance) for all glycans with bisecting GlcNAc.
- High mannose level = Sum of (% abundance) for all high-mannose glycans.
- Sialic Acid/Galactose ratio = Sialylation/Galactosylation.

## RESULTS

### 

#### 

##### Overview

Because the participating labs selected their own methods of analysis and these methods can differ in many ways, merits and drawbacks of the methods can only be discussed in general terms. There are two principal factors distinguishing output: the number of different glycans reported and how the structures of those glycans were determined. High mass accuracy mass spectrometry is currently capable of determining compositions of glycans over a wide range of abundance and therefore can yield the largest number of different glycans, it is limited in isomer identification because spectra of different isomers are often indistinguishable and do not generally contain sufficient information for full structure determination. For example, glycopeptide fragmentation appears incapable of yielding complete glycan structural information. On the other hand, defined structures may be determined using chromatographic methods, coupled with standard materials and labeling or use of enzymes (exoglycosidases) capable to removing selected outer glycans. However, only a limited number of standard glycans are available and enzymatic methods are of limited use in complex glycan mixtures. In the absence of direct structural information, structures are generally represented based on biological inference, which are, in effect, informed guesses.

Sample preparation methods, ranging from enzymatic glycan release and labeling to protein digestion constitute a major source of variation. The effectiveness of these methods depends as much on laboratory skill than specific method and are, in effect, a hidden source of variation in these studies.

Differences in the objectives of the labs is a major contributor to the diversity of methods and results. Probably the most critical measurements are made by biopharmaceutical companies who rely on glycan determinations for both product quality and government approval. Consequently, they generally use well-established, conservative methods that often involve several targeted, derivatized glycans with precisely defined established chromatographic methods. Further, they may limit their number of analytes to only the major glycans considered. Other groups, such as instrument companies and some academic institutions seek to maximize the number of glycans identified, which generally sacrifices structural information. Other labs made these measurements for various educational and internal quality control purposes whereas others wish to develop or demonstrate new methods of analysis.

##### Demographics of Laboratories

One hundred eighteen laboratories responded to the call for participation. However, because of challenges with timing, personnel, shipping (bad weather, customs delay), legal, technical (instrument, freezer malfunction), and other issues, several laboratories had to drop out of the study. Samples were sent to 90 laboratories; 76 laboratories submitted 103 reports. Supplemental Fig. S1*A* shows a map of the participating laboratories from Europe (42%) and North America (38%), Asia (18%), and Australia (2%). Laboratories were primarily from the industry sector, with almost half of these laboratories from biopharmaceutical companies, as shown in supplemental Fig. S1*B*.

##### Glycosylation Analysis Methods Used by Participating Laboratories

Table I summarizes the glycosylation analysis methods used by laboratories in this study. Out of 103 reports, 74% analyzed released glycans, 20% used glycopeptides, and 6% used intact protein and protein fragments. Fluorescently-labeled glycans were commonly analyzed by LC-FD or LC-FD-MS methods except for APTS-labeled glycans, which were analyzed by CE-LIF exclusively. Reduced and permethylated glycans were analyzed solely by MS (using MALDI, direct infusion- (DI) and LC-electrospray ionization techniques). Hydrophilic interaction liquid chromatography (HILIC) is the commonly used chromatographic method for glycans labeled with 2-AB, glycosylamine, and procainamide fluorophores whereas porous graphitized carbon (PGC) was used for reduced glycans.

**Table I:** Overview of analytical techniques for mAb glycosylation analysis used in this interlaboratory study

Analyte	Derivatization	Analytical method	Chromatography	Identification	Quantification
Glycan (76)	2-AB labeling (20)	LC-FD (15)	HILIC (19)	MS mass (5)	PA (19)
		LC-FD-MS (4)	RP (1)	RT std (8)	MS int (1)
		LC-MS (1)		RT GU (5)	
				RT GU & exo (2)	
	Glycosylamine labeling (18): InstantPC	LC-FD (6)	HILIC (17)	MS mass (8)	PA (16)
	InstantAB	LC-FD-MS (8)	RP (1)	MS/MS (2)	MS int (2)
	Rapifluor	LC-MS (4)		RT std (6)	
				RT GU (1)	
				MS mass & RT GU (1)	
	APTS labeling (6)	CE-LIF (6)	CE (5)	exo (2)	PA (4)
			None (1)	MT std (1)	PH (2)
				MT GU (2)	
				MT std & exo (1)	
	Permethylation (6)	MALDI-MS (4)	None (4)	MS mass (4)	MS int (4)
		DI-MS (1)	RP (2)	MS/MS (1)	PA (2)
		LC-MS (1)		MSn (1)	
	Procainamide (6)	LC-FD-MS (5)	HILIC (6)	MS mass (3)	PA (6)
		LC-FD (1)		RT std (1)	
				RT GU (1)	
				All + MS/MS & exo (1)	
	Reduction (5)	LC-MS (5)	PGC (5)	MS mass (2)	MS int (2)
				MS/MS (2)	PA (3)
				MS mass & MS/MS (1)	
	None (4)	LC-MS (2)	HILIC (1)	MS mass (3)	PA (2)
		MALDI-MS (1)	IC (1)	RI (1)	MS int (1)
		HPAEC-PAD (1)	None (1)		sum isotope pks (1)
			PGC (1)		
	Ethyl esterification (3)	MALDI-MS (3)	None (3)	MS mass (3)	sum isotope pks (2)
					isotopic dil, (1)
	2-AA labeling (2)	LC-FD (1)	HILIC (1)	MS/MS (1)	PA (1)
		MALDI-MS (1)	None (1)	RT std (1)	MS int (1)
	2-AA & permethylation (1)	LC-MS (1)	HILIC (1)	MSn (1)	PA (1)
	2-aminopyridine labeling (1)	LC-FD,MS (1)	RP (1)	MS mass & RT std (1)	PA (1)
	4-AA labeling (1)	LC-FD,MS (1)	HILIC (1)	RT std (1)	PA (1)
	INLIGHT (1)	LC-MS (1)	RP (1)	MS mass & MS/MS (1)	PA (1)
	Phenylhydrazine (1)	MALDI-MS (1)	None (1)	MS/MS (1)	MS int (1)
	p-toluidine (1)	MALDI-MS (1)	None (1)	MS (1)	MS int (1)
Glycopeptide (21)	None (18)	LC-MS (16)	RP (17)	MS/MS (9)	PA (12)
		CE- MS (1)	None (1)	MS (7)	MS int (4)
		MALDI-MS (1)		MS mass & MS/MS (2)	sum isotope pks (2)
	Dimethylamidation (1)	MALDI-MS (1)	None (1)	MS mass (1)	sum isotope pks (1)
	Ludger V-tag (1)	LC-FD (1)	HILIC (1)	RT std (1)	PA (1)
	Reduction (1)	CE-MS (1)	CE (1)	MS/MS (1)	MS int (1)
Protein fragment (3)	None (3)	LC-MS (3)	CE (1)	MS mass (3)	MS int (3)
			RP (1)		
			SEC (1)		
Intact protein (2)	None (2)	LC-MS (1)	RP (1)	MS mass (2)	MS int (2)
		DI-MS (1)	None (1)		
Intact, fragments, glycans (1)	None (1)	LC-MS (1)	PGC (1)	MS mass, MS/MS & exo (1)	MS int (1)

Number in parenthesis indicates number of laboratories.

Additional abbreviations: std = standard; int = intensity; dil = dilution; sum = summation; pks = peaks.

In Table I, glycopeptides were typically analyzed without derivatization (18 out of 21 reports) by reversed-phase LC-MS (16 out of 21 reports). MS techniques (MS mass, MS/MS fragmentation data, or a combination) were frequently used for identification whereas MS peak area, MS intensity, and summation of isotope peaks were used for quantification. Supplemental Fig. S2 shows an example of fragmentation data of glycopeptides using LC-MS/MS analysis. HCD spectra at 40% normalized collision energy (supplemental Fig. S2*A*) shows the peptide backbone (supplemental Fig. S2*B*), oxonium ions (supplemental Figs. S2*C* and S2*J*), and glycan fragmentation (supplemental Fig. S2*D*–S2*I*). Oxonium ions have been used to screen for the presence of glycopeptides and glycan motifs (28,29). The laboratory used oxonium ions from a HexNAc (*m/z* 168.07, *m/z* 186.08, and *m/z* 204.09) to screen for the presence of glycopeptides (supplemental Fig. S2*C*). Presence of *m/z* 512.20 oxonium ion is specific for antenna fucosylation (30) (supplemental Fig. S2*E*); *m/z* 290.09 and *m/z* 308.10 oxonium ions are specific for NeuGc residues (supplemental Fig. S2*F*); and *m/z* 528.19 is indicative of a trisaccharide having 2 hexoses and 1 *N*-acetylhexosamine., *e.g.* Gal-Gal-GlcNAc (supplemental Fig. S2*G* and S2*H*). For high mannose glycans, the absence of GlcNAc antenna could result in hexose oxonium ions *m/z* 127.04, *m/z* 145.05, and *m/z* 163.06 (supplemental Fig. S2*J*). The laboratory screened glycopeptides for high-mannose glycans using *m/z* 163.06.

Intact proteins and protein fragments were analyzed mostly using LC-MS, as shown in Table I. Separation was performed by CE, RP, SEC, or PGC; identification was performed by MS mass or MS/MS fragmentation with exoglycosidases; and quantification by MS intensity.

Supplemental Table S1 lists the analytical approaches by laboratory sector. Here, a disparity could be observed in the choice of methods. Biopharmaceutical company laboratories preferred the well-established method involving fluorophore-labeled glycans for their analysis (19 out of 21 laboratories); whereas two used glycopeptide analyses. University laboratories, however, primarily used either more generic mass spectrometry-based methods of glycopeptide analysis (14 out of 32 laboratories) or non-fluorescent glycan (12 out of 32 laboratories) approaches. Protein fragment (*n* = 3) and intact protein (*n* = 2) techniques were used to demonstrate the technology - they are not listed in supplemental Table S1 to protect laboratory anonymity.

##### Glycan Identification

The data reporting template (24) included 54 glycan compositions for 68 glycan structures, however an additional 62 compositions and 71 other structures were reported by the participating laboratories. A total of 116 compositions and 139 structures were identified. Supplemental Table S2 lists the glycan compositions and isomers identified by laboratories in this study (24). Supplemental Table S3 lists all the quantified and derived values for NISTmAb and mod-NISTmAb for glycan compositions and glycan structures (24). Independent of this study, glycosylation of NISTmAb was previously characterized by three laboratories using HILIC with fluorescence detection of 2-AB-labeled *N*-glycans and collectively found 24 glycan peaks (28, 31). Another work using 1D- and 2D-LC-MS/MS for the analysis of glycopeptides found 60 glycan masses on NISTmAb (16).

**Fig. 1.**
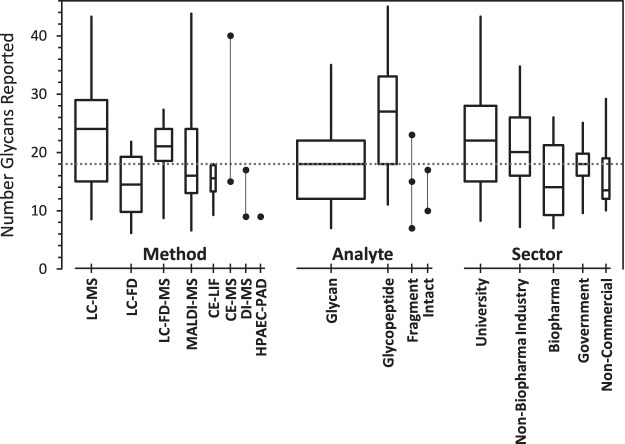
**Number of unique glycan compositions reported, grouped by method, analyte, and sector.** The boxes span the central 50% of reported values, 25% to 75%; the whiskers span the central 90%, 5% to 95%; the central line marks the median, 50%. Box widths are proportional to the number of reports. Groups within each category are presented in order of decreasing number of reports. Solid circles represent individual results within categories of fewer than six reports. The dotted line marks the median number of compositions reported in the 103 reports provided by 76 laboratories.

The number of glycan compositions reported by each laboratory ranged from 4 to 48. Most reports listed about the same number of glycan compositions for each of the two samples. Fig. 1 summarizes the number of unique glycan compositions reported for NISTmAb and/or mod-NISTmAb samples as a function of the laboratory's analytical method, analyte, and organizational type. On average, more compositions were reported by laboratories (1 using MS-based methods, (2 analyzing glycopeptides, and (3 that were university-based. However, the wide range in the number of compositions reported within most of the groups suggests that the technology is not the major determinant.

**Fig. 2.**
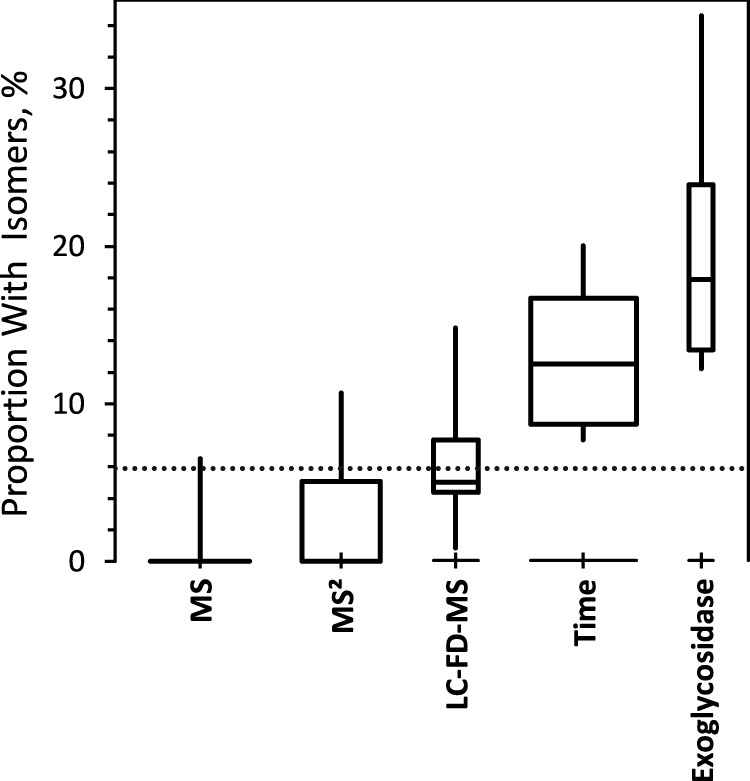
**Proportion of glycan compositions reported as isomers.** The boxes span the central 50% of reported values, 25% to 75%; the whiskers span the central 90%, 5% to 95%; the central line marks the median, 50%. Box widths are proportional to the number of reports. Categories are presented in order of increasing median proportion. The dotted line marks the median proportion of compositions reported as isomers.

Fig. 2 summarizes the proportion of compositions that were reported with isomeric information as a function of identification method. On the average, laboratories that used exoglycosidases reported the greatest number of isomeric information, followed by retention times, then by FD, then by MS/MS. Surprisingly, about one-fourth of the data sets that nominally used accurate mass (MS) identification identified some isomers.

##### Glycan Quantification

Laboratories were asked to report quantitative values for each glycoform as proportions relative to the sum of all glycoforms detected. Table II lists the consensus median abundances of glycan compositions and glycan structures on NISTmAb that were reported at least six times. The three compositions [h3n4f1], [h4n4f1] and [h5n4f1] are the most commonly reported and the most abundant compositions; together, they account for more than 85% of the total signal intensity. Although the normalization factors (the sum of signals) for the different data sets are not based on the same compositions nor the same number of compositions, the dominance of [h3n4f1] and [h4n4f1] ensures that the reported proportions are comparable across data sets. However, the differences among the normalization factors are a source of variability. Other approaches such as normalizing to the most abundant few glycoforms could be pursued in future studies.

**Table II:**
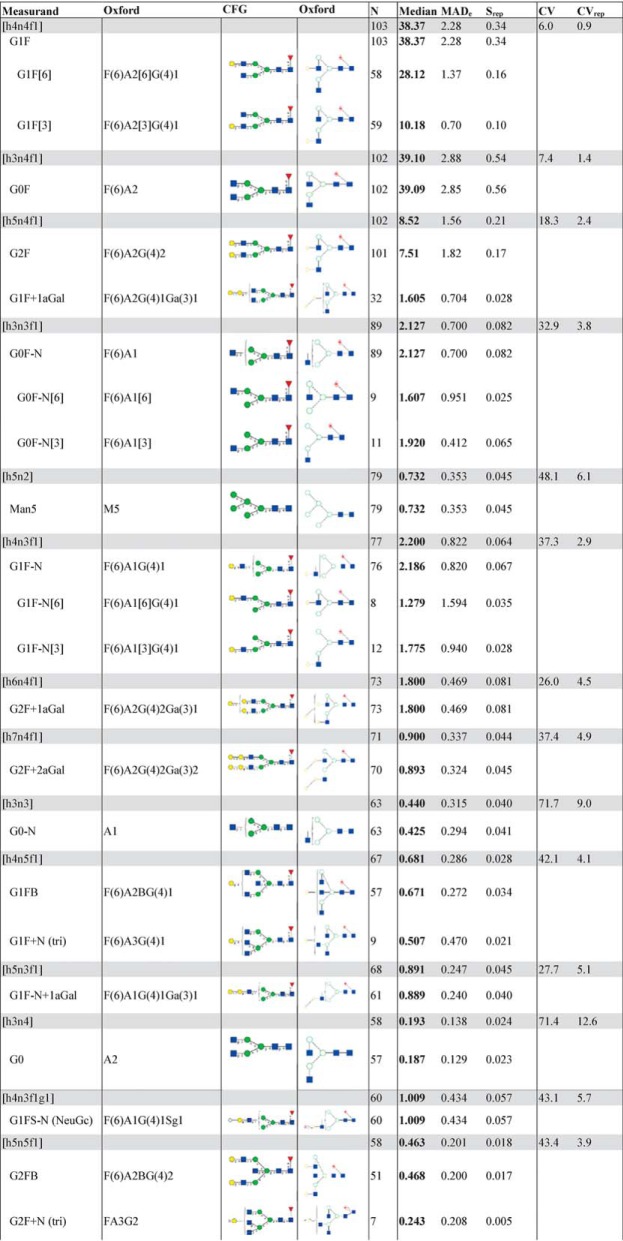

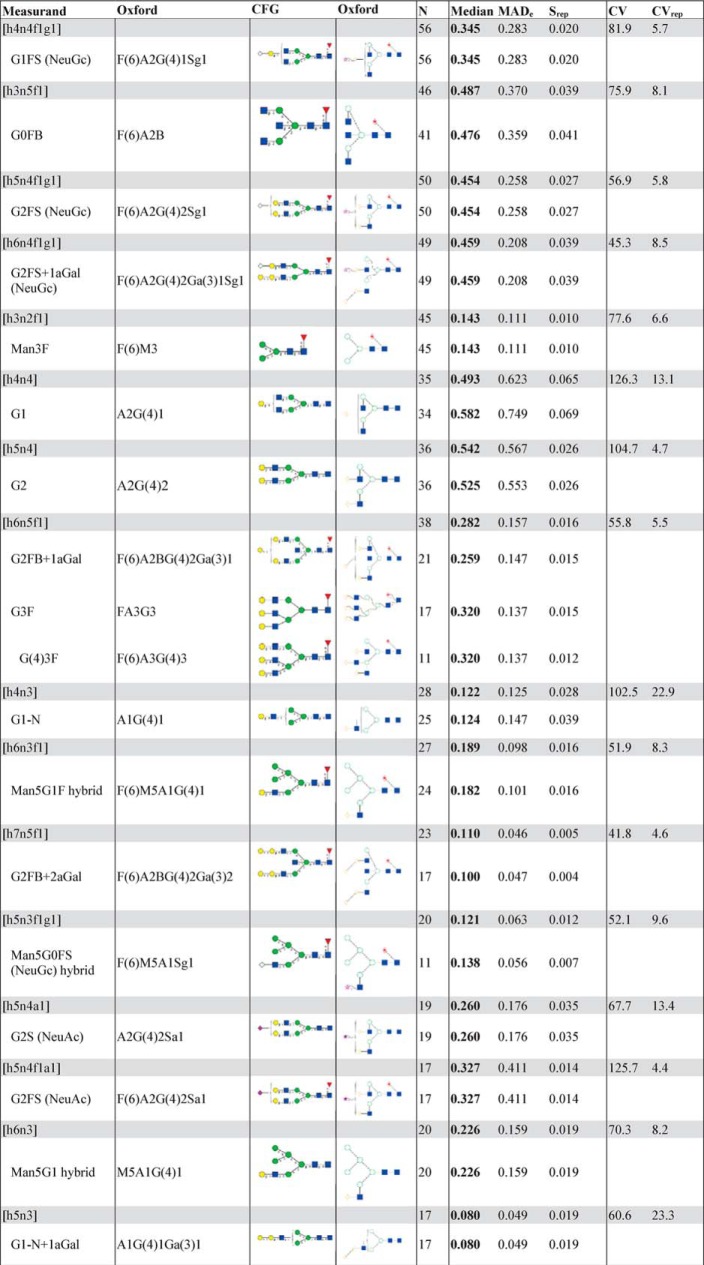

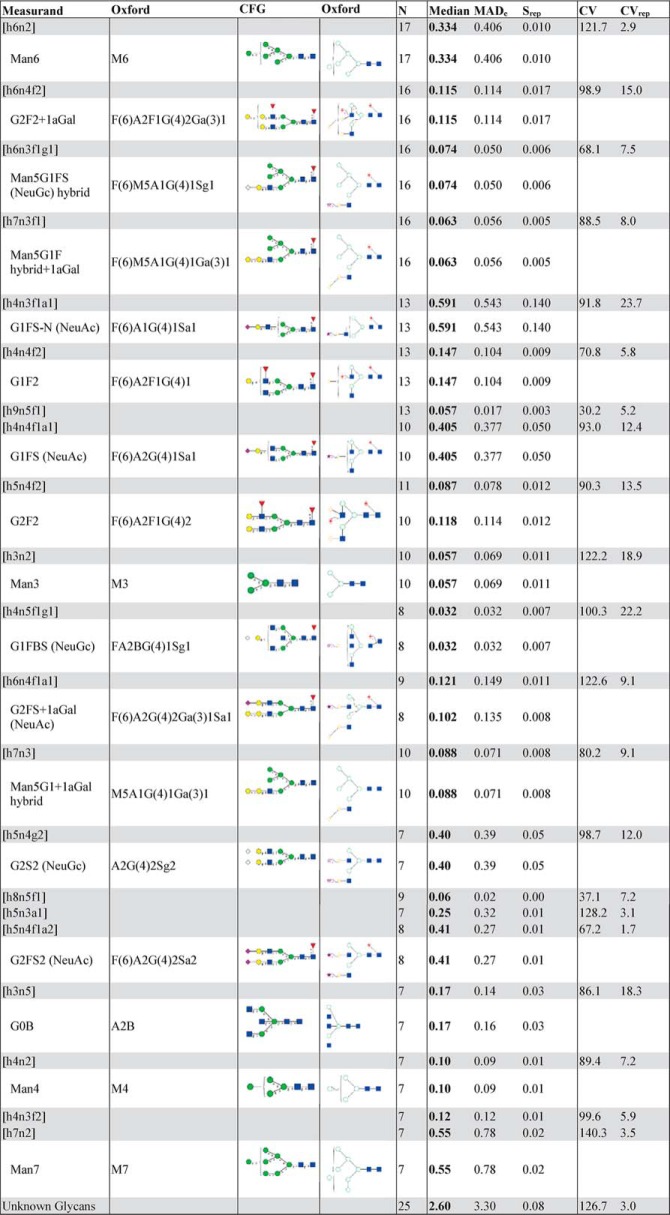
Community's consensus abundances of glycans in NISTmAb PS 8670 reported by laboratories at least six times. Glycan compositions are arranged by decreasing number of values (N). Supplementary Table S2 lists all glycan structures and names. Supplementary Table S3 lists all the community's consensus values

*n* = number of values; consensus median = consensus median (50th percentile or 2nd quartile, expressed as percent of total composition) of the distribution of the reported results; MAD_E_ = median absolute deviation; S_rep_ = a robust estimate of the expected repeatability, the median SD for glycan compositions with at least six results; CV = robust coefficient of variation (MAD_E_/median); CV_rep_ = (S_rep_/median).

Laboratories were asked to report their results as percent abundances normalized such that they summed to 100% per sample. Supplemental Fig. S3 shows a histogram of the sum of the unique glycan composition values for NISTmAb and mod-NISTmAb as reported by the laboratories (*n* = 206, two samples per laboratory). Although results in most data sets summed to 100%, the sums ranged from 88% to 122%. Some laboratories assigned percent abundances to unidentified glycans; the sum of these values was reported as one entry called “Unknown Glycans” and added to the abundance sum for that sample.

The number of replicate values per reported composition or structure per sample ranged from one to nine. The nature of these values ranged from purely technical replicates (multiple measurements of the same preparation) to process replicates (single measurements of multiple independent preparations). In all cases where two or more replicate values were reported, the values were summarized as their mean and standard deviation (SD).

Some data sets reported replicate values equal to zero, not detected (ND), and not quantified (NQ). These values can be ignored when there are no quantitative values in a set of replicates but cause numerical instability when there is at least one quantitative value in the set. Various options were explored for handling these situations in a uniform manner including: treating non-numerical results as zero, replacing zeros and non-numerical values with the data set's minimum reported value (MRV, the smallest reported numerical value of a data set), or replacing them with the data set's limit of reporting (LoR, the extrapolated smallest value of a data set). Replacement with the LoR provided slightly smaller SDs than replacement by the MRV. Supplemental Fig. S4 shows the LoR for one set of results. Gray lines are traces of the unique non-zero values reported in each set of results, where the numbers are ordered by decreasing value. If the true amounts of the minor glycans are randomly distributed and all results reflect the same level of analytical effort, a best-fit line to the right-tail of the trace estimates the LoR for that set. As shown in supplemental Fig. S5, most of the LoRs agree well for MRVs above about 0.05%. Below this value, many of the sets contain a few values many-fold smaller than their LoR. This may reflect special interest in selected glycan components rather than reporting issues with the less-abundant glycans. The LoR values in the data set may be more representative of the analytical sensitivity of a measurement system than is the MRV.

##### Derived Attribute Quantities in NISTmAb

Table III shows the degree of galactosylation, sialylation, fucosylation; levels of bisecting GlcNAc and high-mannose; and the sialic acid/galactose ratio in NISTmAb. These values are estimated from the consensus median values of the glycan compositions. Calculations are based on previous works by Wuhrer (27) and are designed to reflect biosynthetic pathways (32) and, to some extent, enzyme activity. In addition, these glycosylation traits relate to differences in effector functions of monoclonal antibodies and circulation half-time for other therapeutic glycoproteins.

**Table III:** Derived attribute quantities for NISTmAb PS 8670, estimated from the consensus median values of the glycan compositions

Features	Number of Labs	25%	Median	75%
Galactosylation	32	31.78	36.21	43.30
alpha-Galactosylation	13	3.00	3.77	4.97
Sialylation	18	2.26	3.48	6.98
NeuAc sialylation	6	0.71	1.25	2.83
NeuGc sialylation	12	1.55	2.23	4.16
Core fucosylation only	37	92.36	103.95	118.23
Antenna fucosylation	3	0.25	0.38	0.73
Bisecting GlcNAc	7	1.49	2.17	3.83
High mannose	6	1.04	1.92	3.42
Sialic Acid/Galactose Ratio		0.07	0.10	0.16

All antennae were assumed to be available for galactosylation by most galactosyltransferases. Antennae galactosylation may be a reasonable proxy for enzyme activity and may therefore reflect regulation of galactosylation process in a biological system. Thus, galactosylation levels were expressed by calculating the number of galactosylated (occupied) antennae divided by the total number of antennae of the specific glycan. Only glycans identified with galactose residues were included in the calculations. For biantennary glycans, the galactosylation levels are 0.0, 0.5, and 1.0 for 0, 1, and 2 galactoses, respectively. For triantennary glycans, the galactosylation levels are 0.0, 0.33, 0.67, and 1.0, reflecting the presence of 0, 1, 2, or 3 galactosylated antennae. For NISTmAb, the median degree of galactosylation is 36.2% with alpha-galactosylation at 3.8%, as shown in Table III.

For sialylation levels, the same principle is applied, *i.e.* the sialylation per antenna was calculated. For NISTmAb, NeuAc and NeuGc sialylation were observed at a medium value of 1.3% and 2.2%, respectively. The ratio of sialic acid per galactose was calculated as 0.1%. This value reflects the sialylation activity, *i.e.* whether available acceptor positions have been sialylated.

Monofucosylation was interpreted as core fucosylation. NISTmAb had very high levels of core fucosylation (median of 104%; values exceeding 100% are artifacts of the variable normalization factors). The antenna fucosylation (manifested as difucosylation) at 0.38% was calculated separately because the interaction between core and antenna fucosylation is assumed to be minimal.

##### Issues with Glycosylation Analysis Methods

Laboratories reported challenges in identifying and quantifying glycans. Some laboratories reported their analysis at the composition level only and did not differentiate isomeric species; some laboratories analyzed at the glycan isomer level and had challenges in identifying co eluting or same mass species. These issues are usually method dependent, as shown in supplemental Table 4. Some glycan structures were supported by MS/MS and other structures were inferred from similar structures, *e.g.* triantennary structures. Consequently, some abundance values were assigned to triantennary structures instead of bisecting glycans. The same laboratory observed a discrepancy for glycan G1FS N (NeuGc). Compared with quantitative data from subunit analysis, the glycopeptide abundance was higher than subunit abundance. Overall, the laboratory observed that glycopeptide abundances were in good accordance with the subunit data with slightly lower values for G0F and G1F in the glycopeptide analysis.

One laboratory analyzed protein fragments by LC MS that had masses up to 25 kDa. One or two nominal mass differences were challenging to distinguish using their technique.

Another laboratory analyzed 2-AB glycans using HILIC LC FD with comparison to retention time of standards. It was difficult to distinguish glycans that coelute, *e.g.* G2 and Man6. Moreover, the laboratory was unable to identify glycan peaks present in the samples but absent in their lab-designated standard sample.

Supplemental Table S4 lists the advantages of certain methods as described by laboratories. Sialic acid specific derivatization of 2-AB or ethyl esterified glycans analyzed by LC FD or MALDI MS could confirm presence of terminal α2 6 linked NeuGc in glycans.

Intact protein analysis could give the G0F/G1F, G1F/G2F, G0/G0F, G0F N/G0F, G2F+1aGal/G2F glycoforms present in the monoclonal antibodies. Due to the cleavage of glycopeptides or glycans from the protein, analysis using these two analytes could not provide this specific information.

2-AB glycans analyzed by LC FD using glucose units and APTS labeled glycans analyzed by multiplexed capillary gel electrophoresis (xCGE) using migration time of standards and exoglycosidases could distinguish between isomers.

### Additional Information on NISTmAb

Some laboratories performed unique analyses, resulting in additional information on the glycosylation of NISTmAb

#### 

##### Absolute Glycan Amounts

One laboratory determined absolute glycan amounts in the samples by employing isotopic dilution methods, using ^13^C-labeled *N*-glycans as internal standards followed by MALDI-TOF MS analysis. For example, the absolute amounts of three glycans in NISTmAb were reported to be:

G0F^a^: (626.7 ± 7.5) pmol per 100 μg NISTmAb
G2F: (110.8 ± 5.9) pmol per 100 μg NISTmAb
G2: (19.0 ± 3.8) pmol per 100 μg NISTmAb

##### Glycoforms in Intact Samples

One laboratory analyzed intact mAb samples using LC-MS and identified glycoforms on the two Fc portions that were analyzed. Example glycoforms are G0/G0F, G0F/G1F, G1F/G2F, G0F-N/G0F, and G2F+1aGal/G2F. Abundance values for these glycoforms are shown in supplemental Table S3, bottom rows.

##### Unknown Modifications

One laboratory found an unknown delta mass of + 1856 Da at 0.40% abundance in NISTmAb by LC-MS. Another laboratory detected + 54 Da unidentified protein modification using ^1^H-NMR and MS. The latter found a glycan present in <3% with no branching at the central β-Mannose, *i.e.* there is only one arm present with a terminal NeuGc and a proximal Fuc.

##### Unglycosylated Forms

One laboratory used protein fragment analysis by C4-LC-MS and observed the unglycosylated form of the samples at 0.60% abundance in NISTmAb. Another participant used glycopeptide analysis by C18-LC-MS and detected the unglycosylated form at 0.91% abundance in NISTmAb, as confirmed by high mass accuracy MS (< 3 parts per million (ppm^b^) mass deviation).

##### Glycan Motifs

One laboratory employed lectin microarrays on intact proteins (33). The participant found that NISTmAb has more core fucosylation whereas mod-NISTmAb has slightly more terminal fucosylation. Both samples hint at the presence of α2,6 sialylation and show similar patterns for hybrid/lower order mannose *N*-glycans, indicating no strong presence of complex glycans.

## DISCUSSION

### 

#### 

##### Sample Ratios Demonstrate Comparability

**Fig. 3.**
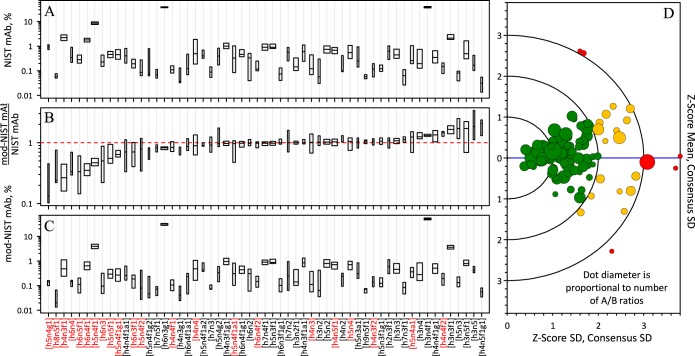
**Summary results for the 57 most frequently reported unique glycan compositions.** Box plots for *A*) mod-NISTmAb, *B*) mod-NISTmAb/NISTmAb ratio, and *C*) NISTmAb PS 8670. Glycan compositions in red have terminal β1,4-gal as their dominant structure. Each box represents the distribution of the central 50% of the mean of the reported replicate values for one glycan. The horizontal middle line in each box represents the consensus median. The width of each box is proportional to the square root of the number of values defining the distribution. The dashed red line in the display of the Fig. 3*B* denotes the expected ratio, 1.0, when a glycan result is the same in mod-NISTmAb as it is in NISTmAb. Glycans are sorted in order of increasing mod-NISTmAb/NISTmAb ratio. *D*) Targetplot summary of mod-NISTmAb/NISTmAb ratios relative to the consensus medians. Each dot represents one set of results. Dot diameter is proportional to number of mod-NISTmAb/NISTmAb ratios reported. The dots are color-coded by distance from the (0, 0) origin: dots within two comparability units are colored green, between two and three units are colored yellow, and greater than three units are colored red. The “Z-score Mean” axis displays the average bias estimated as the mean of the “Z-score” values of the ratios. The “Z-Score SD.” axis displays the variability of individual bias estimates, estimated as the standard deviation of the Z-scores.

To examine the between-data set differences in the measurements of the two samples, mod-NISTmAb/NISTmAb ratios were calculated for the 57 glycan compositions that were reported at least six times for either NISTmAb or mod-NISTmAb. Since the number and identity of the reported glycan compositions in the two samples were nearly the same within each data set, these ratios are insensitive to the normalization factors. Figs. 3*A* and 3*C* display the NISTmAb and mod-NISTmAb measurement distributions of these compositions as boxplots. Fig. 3*B* displays the mod-NISTmAb/NISTmAb ratios. Supplemental Table S3 lists the values of these ratios.

Every box in Fig. 3 spans the central 50% of the reported or calculated values, with the horizontal middle line denoting the consensus median. The compositions are sorted in order of increasing mod-NISTmAb/NISTmAb median. The width of each box is proportional to the square root of the number of values defining the distribution, so the wider the box, the more laboratories reported that glycan. For example, 102 of the 103 data sets identified [h3n4f1] and [h4n4f1], which have the widest boxes. The dashed red line in Fig. 3*B* denotes the expected ratio, 1.0, when a glycan result is the same in mod-NISTmAb as it is in NISTmAb. Ideally, glycan structures with terminal β1,4-gal should fall below this red line because β1,4-galactosidase, an enzyme that specifically cleaves terminal β1,4-gal, was added to a portion of the mod-NISTmAb. Glycan compositions colored red in the *x* axis have terminal β1,4-gal as their dominant structure. As expected, most of these glycans fall below the red line, *i.e.* they have lower abundance in mod-NISTmAb than in NISTmAb.

Fig. 3*D* displays the average variability and bias of the mod-NISTmAb/NISTmAb ratios relative to the consensus medians in a form sometimes called a “targetplot” (34). Each dot marks the summary score for the unique glycan compositions in one set of results. The vertical axis displays the mean bias or “concordance” of the ratios: *z*_*i*_ = (∑_*j*_(*x*_*ij*_ − *x̄*_*j*_)/*s*_*j*_)/*n*_*j*_, where *x_ij_* is the ratio for the *j*^th^ composition reported in the *i*^th^ data set, *x̄*_*j*_ is the consensus location of the *j*^th^ composition, *s_j_* is the consensus dispersion for that composition, and *n_j_* is the number of data sets that report values for that composition in both samples. Because the distributions of the ratios for most compositions are heavily-tailed, the consensus location and dispersion are estimated using robust estimators: the median for location and the scale-adjusted median absolute deviation from the median (MAD_E_) for dispersion. The horizontal axis displays the variability of bias estimates, estimated as the SD. or “apparent precision” of the biases:

$$s\left(z_{i}\right)=\sqrt{\sum_{j}{\left(\left(x_{ij}-{\bar{x}}_{j}\right)/s_{j}\right)}^{2}/\left(n_{j}-1\right)}$$

The semicircles mark one, two, and three “comparability” distances from the ideal (*z_i_*, *s*(*z_i_*)) value of (0, 0):

$$d_{i}=\sqrt{z_{i}^{2}+s^{2}\left(z_{i}\right)}$$

The targetplot dots are color-coded by distance from the (0, 0) origin: dots within two comparability units are colored green, between two and three units are colored yellow, and greater than three units are colored red. These codes roughly indicate “Good”, “Moderate”, and “Questionable” agreement with the consensus mod-NISTmAb/NISTmAb ratio estimates.

**Fig. 4.**
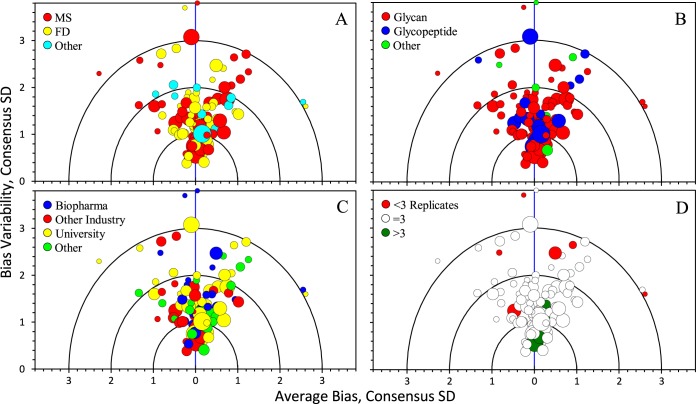
**Targetplot summary of mod-NISTmAb/NISTmAb ratios relative to the consensus medians.** Each dot represents one set of results. Dot diameter is proportional to number of ratios reported. The “Average Bias” axis displays values estimated as the mean of the “Z-scores” of the ratios. The “Bias Variability axis displays values estimated as the standard deviation of the Z-scores. The subplots are colored by: A) analytical technique, B) analyte, C) laboratory type, and D) number of replicates.

Fig. 4 displays the same targetplot colored by analyte, analytical technique, organizational type, and number of replicates. No systematic trend was apparent in any of the parameters.

##### Youden Two-Sample Plots of Glycan Compositions

**Fig. 5.**
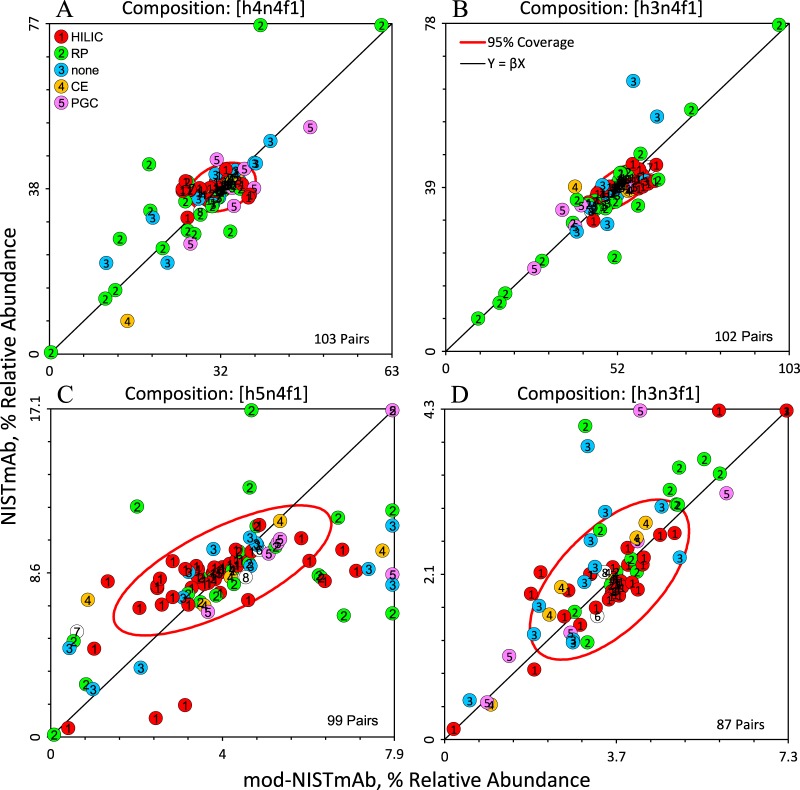
**Youden two-sample plots for the four most abundant glycan compositions in NISTmAb.** Each panel displays the bivariate distribution for one composition. *A*) [h4n4f1], *B*) [h3n4f1], *C*) [h5n4f1], and *D*) [h3n3f1] (see Materials and Methods for key). The panels are centered on the univariate medians and scaled to display all values from 0 to twice the median. Values that are greater than twice the median are assigned a value of twice the median. The ellipse includes about 68% of the pairs (≈ 1 SD). The diagonal line represents the expected relationship when measurement systems have the same bias for both samples. Each symbol represents the (mod-NISTmAb, NISTmAb) pair for one data set. Symbols are coded and labeled by separation technique.

Fig. 5 shows Youden two-sample plots for the four most abundant glycan compositions, [h3n4f1], [h4n4f1], [h5n4f1] and [h3n3f1]. Supplemental Fig. S6 presents similar plots for all compositions with quantitative results for both samples in at least six data sets. Each dot in these panels represents one (mod-NISTmAb, NISTmAb) pair from one data set. The median of each sample is used as the univariate estimate of distribution location because it is not as vulnerable to extreme values as is the mean. The center of each panel (for [h3n4f1], the values (52, 39)) represents the consensus location of the resulting bivariate distribution. The ellipse in each panel is constructed to enclose data pairs that are consistent with the consensus medians at an approximate 68% (one sigma) level of confidence. These ellipses are defined by (1 the univariate medians, (2 the MAD_E_ robust estimates of the univariate SDs, (3 the bivariate correlation between the two distributions, and (4 a factor that provides a stated coverage probability.

When the two samples present similar measurement challenges, Youden two-sample plots graphically separate random within-data set measurement imprecision from systematic between-data set bias (35, 36). Pairs of measurements that reflect the same proportional bias will fall along the 45° line. This often indicates a calibration issue. Pairs of measurements that lie well away from the line indicate sample-specific interferences or measurement systems that are not in adequate statistical control.

The dots in Fig. 5 are colored by separation method. Essentially all laboratories that used HILIC separation are within the ellipse for [h3n4f1] and [h4n4f1], are within the ellipse or lie along the diagonal for [h3n3f1] but show significant scatter for [h5n4f1]. The pattern of off-diagonal results for [h5n4f1] suggests that for this composition the two samples presented several measurement systems with different measurement challenges.

##### Measurement Repeatability Better for More Abundant Glycans

**Fig. 6.**
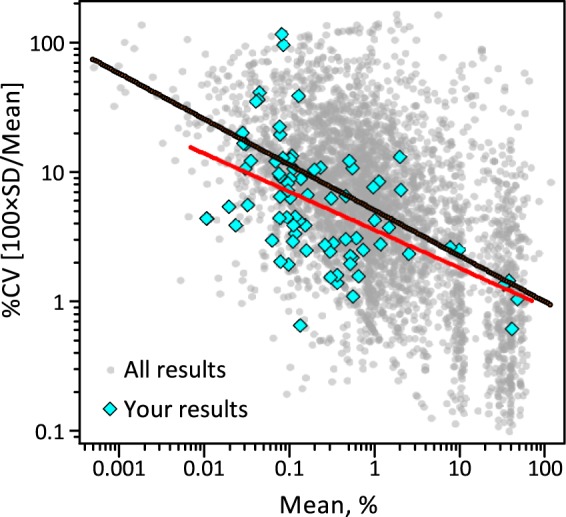
**Scatterplot of the relationship between measurement repeatability, estimated as the CV, and glycan amount, estimated as the mean of the replicates.** The black line represents a consensus power curve fit to all available (mean of replicates, relative standard deviation) pairs, denoted by the light gray dots: CV = 5.0 × Mean^−0.35^ (or SD = 0.050 × Mean^0.65^). The red line is the power curve fit to the pairs, denoted by the blue diamonds, reported in one data set. The measurement repeatability or this data set is somewhat better than average.

Metrological repeatability is defined as the variation in measurements taken by a single person or instrument on the same sample, under the same conditions, and in a short period of time. Fig. 6 shows a scatterplot of the relationship between measurement repeatability, estimated as the coefficient of variation expressed as percentage (CV), and glycan amount, estimated as the mean of the replicates, for one exemplar laboratory. The black line represents a simple consensus power-law: CV = 5.0 × Mean^−0.35^ (or, expressed as SD = 0.050 × Mean^0.65^). Note that CV is not constant for all glycan amounts but rather generally increases with decreasing amount. This trend is closely related to Horwitz's observation that the interlaboratory study CV generally increases with decreasing analyte concentration regardless of the analytical method or number of laboratories (37). It has been speculated that this empirical trend arises more from cost-benefit considerations than intrinsic analytical limitations (38).

##### Repeatable Measurements are Closer to Community's Consensus Values

**Fig. 7.**
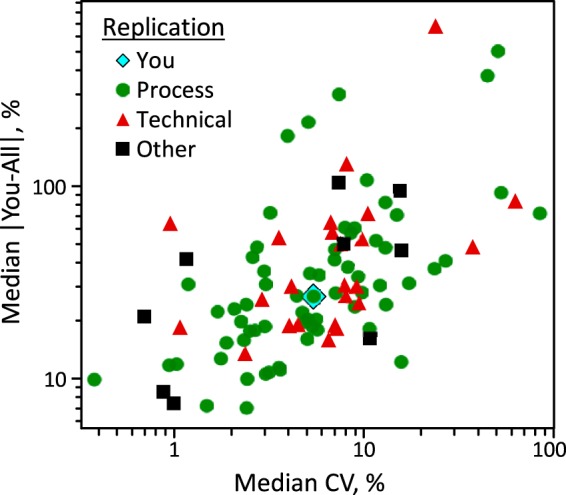
**Scatterplot of the closeness to consensus of the reported values as a function of measurement repeatability estimated as CV.** The symbols are coded by the user-stated nature of the reported replicates. The plot shows the data point, in blue diamond, of one data set.

The extent of agreement between a given laboratory's reported values and the study's consensus median values is a strong function of the laboratory's measurement repeatability. Thus, establishing within-laboratory repeatability is critical to the harmonization of glycosylation analysis methods between-laboratories. Fig. 7 shows a scatterplot of the closeness to consensus of the reported medians as a function of measurement repeatability. “Closeness” is estimated as the relative absolute difference between a given result mean and the median of the means provided in all 103 reports: 100× Mean-Consensus Median /Mean. The symbols are coded by the user-stated nature of the reported replicates. Because of the great variability in the results for the various glycans, the over-all repeatability for each laboratory is estimated as the median of the repeatabilities of the reported unique glycan compositions.

##### Trueness of the Consensus Estimates

**Fig. 8.**
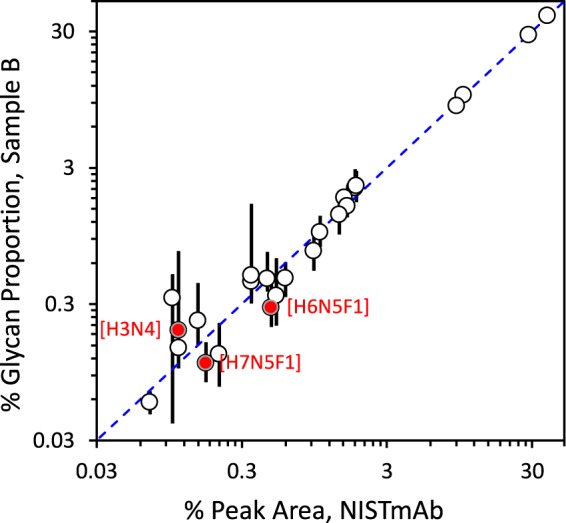
**Comparison Consensus Medians to Published Peak Areas^22^.** Each symbol represents this interlaboratory study's consensus median % proportion as a function of the published peak areas for one composition or defined group of compositions. The bars span the central 50% of the distribution of reported values. The solid red circles denote compositions where the central 50% of the values does not include the published peak area. The dashed line represents equality between the two estimates.

Metrological trueness is the closeness of a result to the best available approximation to its (unknowable) true value. Area-based estimates for 27 resolved NISTmAb peaks of defined composition have been published (22). Unique correspondences can be established between glycan compositions and most of the peak assignments, Fig. 8 demonstrates that the study's consensus medians agree well with published values for composition levels of 1% or more. While agreement diverges with decreasing abundance, only three of the published values are not contained within the study's central 50% distribution.

In broad terms, a large proportion of methods used chromatography for separation followed by identification either by mass spectrometry or chromatographic retention times. Some laboratories combined both for identification. Laboratories that used MS only reported more distinct glycan compositions. Laboratories that used MS with exoglycosidases, RT, FD, and/or MS/MS reported isomers. However, the range in the number of reported compositions within each category is quite large.

## CONCLUSION

Community consensus medians for 57 glycan compositions in NISTmAb were obtained from 103 reports of 76 laboratories. Levels of sialylation (NeuAc and NeuGc), galactosylation (including alpha-galactosylation), and fucosylation (core and antenna) were calculated from consensus medians. These values could be useful in comparing analytical methods for determining glycosylation of a publicly-available material. A unique advantage of using NISTmAb is that because it was produced in NS0 cells, a rich array of glycan compositions with low-abundant variants including NeuGc and alpha-Gal-containing glycans is observed.

More importantly, this study provides an overview of the current state of glycosylation determination in the glycomics community:

1. Most biopharmaceutical company laboratories analyzed glycosylation using fluorescently-labeled glycans whereas most academic laboratories prefer glycopeptide analysis and non-fluorescently-labeled glycan analysis. On average, biopharmaceutical company laboratories reported lower number of glycan compositions than university laboratories.
2. Glycan compositions identified using different methods for determining glycosylation ranged from 4 to 48 compositions. Laboratories that used glycopeptides as analyte, on the average, reported the greatest number of glycan compositions.
3. Agreement to the community's consensus medians did not depend on a specific method or laboratory type but on the measurement repeatability. The better a laboratory's measurement precision the more likely that the laboratory's mean values will agree with the community consensus. Thus, establishing within-laboratory repeatability is critical to the harmonization of glycosylation analysis methods between-laboratories.
4. Most methods used in the different laboratories could be corrected by calibration methods when and if a standard becomes available.
5. Measurement repeatability was better for more abundant glycans. The CVs increased with decreasing glycan abundances. This work is the first attempt to analyze data from the interlaboratory study. Further data mining studies on this large data set could be valuable to the glycomics communty to uncover underlying systematic trends. Examples of potential studies include comparisons of identification methods, quantification methods, or normalization methods. Other areas to explore include assigning degrees of confidence in identification methods (*e.g.* identification by MS1 alone *versus* identification by MS1 with one, two, or three orthogonal values).

The study shows a clear need for harmonization of glycosylation analysis methods. Further understanding of causes of deviations would be useful in developing a harmonized method for glycosylation analyses of mAbs.

Finally, we note that the generally unsatisfactory state of confident identification of less abundant glycan structures. While the increasing sensitivity of mass spectrometry-based methods have revealed an increasingly large number of glycan compositions, the ability to elucidate their structures have not kept pace. In many cases, glycan structures are routinely inferred through biological reasoning. The use of exoglycosidases is helpful in excluding candidates, but of limited value for minor glycans or complex mixtures. Perhaps the emerging field of ion mobility mass spectrometry can aid the distinction of isomers.

## DATA AVAILABILITY

Summary results as provided by the participants, expressed as percentage total glycan, are provided in the Supplemental Information.

## Supplementary Material

Supplemental Data

## Footnotes

^a^ Definition of naming convention of glycans is in the Experimental Procedures section.

^b^ ppm is a unit of mass measurement error (Δm/z)/(m/z) * 1000000, expressed as unified atomic mass units (Daltons) divided by charge.

^1^ The abbreviations used are:

MS: generic mass spectrometry or first stage mass spectrometry
AA: aminobenzoic acid
AB: aminobenzamide
APTS: 9-aminopyrene-1,4–6-trisulfonate
C4: C4 (butyl) desalting column
C8: C8 (octyl) desalting column
CE: capillary electrophoresis
CFG: Consortium for Functional Glycomics
DI: direct infusion
Exo: exoglycosidase
Fab: antigen-binding fragment of a monoclonal antibody
Fc: crystallizable fragment of a monoclonal antibody
FD: fluorescence detection
GU: glucose units
HILIC: hydrophilic interaction liquid chromatography
HPAEC-PAD: high performance anion exchange chromatography with pulsed amperometric detection
IC: ion chromatography
IdeS: immunoglobulin G-degrading enzyme
LC: liquid chromatography
LIF: laser-induced fluorescence detection
LoR: limit of reporting
mAb: monoclonal antibody
MALDI: matrix-assisted laser desorption/ionization
MRV: minimum reported value
MS/MS: tandem mass spectrometry
MSn: nth stage MS
MT: migration time
ND: not detected
NISTIR: NIST internal report
NMR: nuclear magnetic resonance
NQ: not quantified
PA: peak area/integration
PGC: porous graphitized carbon
PH: peak height
PNGase F: Peptide-N-Glycosidase F
PS: primary sample
RP: reversed-phase
RT: retention time
SEC: size-exclusion chromatography
UOXF: Oxford Glycobiology Institute
xCGE: multiplexed capillary gel electrophoresis.

## References

[1] Yang S., Li Y., Shah P., and Zhang H. (2013) Glycomic analysis using glycoprotein immobilization for glycan extraction. Anal. Chem. 85, 5555–5561 23688297 10.1021/ac400761e PMC3696186

[2] Vreeker G. C. M., and Wuhrer M. (2017) Reversed-phase separation methods for glycan analysis. Anal. Bioanal. Chem. 409, 359–378 27888305 10.1007/s00216-016-0073-0 PMC5203856

[3] Ruhaak L. R., Zauner G., Huhn C., Bruggink C., Deelder A. M., and Wuhrer M. (2010) Glycan labeling strategies and their use in identification and quantification. Anal. Bioanal. Chem. 397, 3457–3481 20225063 10.1007/s00216-010-3532-z PMC2911528

[4] Dotz V., Haselberg R., Shubhakar A., Kozak R. P., Falck D., Rombouts Y., Reusch D., Somsen G. W., Fernandes D. L., and Wuhrer M. (2015) Mass spectrometry for glycosylation analysis of biopharmaceuticals. Trac-Trend Anal. Chem. 73, 1–9

[5] O'Flaherty R., Muniyappa M., Walsh I., Stockmann H., Hutson R., Saldova R., and Rudd P. M. (2016) High-throughput sequential glycoprofiling of six abundant glycoproteins IgG, IgA, IgM, transferrin, haptoglobin and alpha-1-antitrypsin in ovarian cancer. Glycobiology 26, 1430–1431

[6] Beck A., Wagner-Rousset E., Ayoub D., Van Dorsselaer A., and Sanglier-Cianferani S. (2013) Characterization of therapeutic antibodies and related products. Anal. Chem. 85, 715–736 23134362 10.1021/ac3032355

[7] Beck A., Wagner-Rousset E., Bussat M. C., Lokteff M., Klinguer-Hamour C., Haeuw J. F., Goetsch L., Wurch T., Van Dorsselaer A., and Corvaia N. (2008) Trends in glycosylation, glycoanalysis and glycoengineering of therapeutic antibodies and Fc-fusion proteins. Curr. Pharm. Biotechno. 9, 482–501 10.2174/138920108786786411 19075687

[8] Hecht E. S., McCord J. P., and Muddiman D. C. (2015) Definitive screening design optimization of mass spectrometry parameters for sensitive comparison of filter and solid phase extraction purified, INLIGHT plasma N-glycans. Anal. Chem. 87, 7305–7312 26086806 10.1021/acs.analchem.5b01609 PMC4664066

[9] Walker S. H., Taylor A. D., and Muddiman D. C. (2013) Individuality normalization when labeling with isotopic glycan hydrazide tags (INLIGHT): a novel glycan-relative quantification strategy. J. Am. Soc. Mass Spectr. 24, 1376–1384 10.1007/s13361-013-0681-2 PMC3769964 23860851

[10] Hu Y. L., Shihab T., Zhou S. Y., Wooding K., and Mechref Y. (2016) LC-MS/MS of permethylated N-glycans derived from model and human blood serum glycoproteins. Electrophoresis 37, 1498–1505 26959726 10.1002/elps.201500560 PMC4964794

[11] Mechref Y., and Muddiman D. C. (2017) Recent advances in glycomics, glycoproteomics and allied topics. Anal. Bioanal. Chem. 409, 355–357 27933360 10.1007/s00216-016-0093-9

[12] Zhou S. Y., Hu Y. L., Veillon L., Snovida S. I., Rogers J. C., Saba J., and Mechref Y. (2016) Quantitative LC-MS/MS glycomic analysis of biological samples using aminoxyTMT. Anal. Chem. 88, 7515–7522 27377957 10.1021/acs.analchem.6b00465 PMC5759044

[13] Yang N., Goonatilleke E., Park D., Song T., Fan G. R., and Lebrilla C. B. (2016) Quantitation of site-specific glycosylation in manufactured recombinant monoclonal antibody drugs. Anal. Chem. 88, 7091–7100 27311011 10.1021/acs.analchem.6b00963 PMC4955800

[14] Hong Q. T., Ruhaak L. R., Stroble C., Parker E., Huang J. C., Maverakis E., and Lebrilla C. B. (2015) A method for comprehensive glycosite-mapping and direct quantitation of serum glycoproteins. J. Proteome Res. 14, 5179–5192 26510530 10.1021/acs.jproteome.5b00756 PMC4670571

[15] Giorgetti J., D'Atri V., Canonge J., Lechner A., Guillarme D., Colas O., Wagner-Rousset E., Beck A., Leize-Wagner E., and Francois Y. N. (2018) Monoclonal antibody N-glycosylation profiling using capillary electrophoresis - Mass spectrometry: Assessment and method validation. Talanta 178, 530–537 29136858 10.1016/j.talanta.2017.09.083

[16] Dong Q., Yan X., Liang Y., and Stein S. E. (2016) In-depth characterization and spectral library building of glycopeptides in the tryptic digest of a monoclonal antibody using 1D and 2D LC-MS/MS. J. Proteome Res. 15, 1472–1486 26990841 10.1021/acs.jproteome.5b01046

[17] Wada Y., Azadi P., Costello C. E., Dell A., Dwek R. A., Geyer H., Geyer R., Kakehi K., Karlsson N. G., Kato K., Kawasaki N., Khoo K. H., Kim S., Kondo A., Lattova E., Mechref Y., Miyoshi E., Nakamura K., Narimatsu H., Novotny M. V., Packer N. H., Perreault H., Peter-Katalinic J., Pohlentz G., Reinhold V. N., Rudd P. M., Suzuki A., and Taniguchi N. (2007) Comparison of the methods for profiling glycoprotein glycans–HUPO Human Disease Glycomics/Proteome Initiative multi-institutional study. Glycobiology 17, 411–422 17223647 10.1093/glycob/cwl086

[18] Wada Y., Dell A., Haslam S. M., Tissot B., Canis K., Azadi P., Backstrom M., Costello C. E., Hansson G. C., Hiki Y., Ishihara M., Ito H., Kakehi K., Karlsson N., Hayes C. E., Kato K., Kawasaki N., Khoo K. H., Kobayashi K., Kolarich D., Kondo A., Lebrilla C., Nakano M., Narimatsu H., Novak J., Novotny M. V., Ohno E., Packer N. H., Palaima E., Renfrow M. B., Tajiri M., Thomsson K. A., Yagi H., Yu S. Y., and Taniguchi N. (2010) Comparison of methods for profiling O-glycosylation: Human Proteome Organisation Human Disease Glycomics/Proteome Initiative multi-institutional study of IgA1. Mol. Cell. Proteomics 9, 719–727 20038609 10.1074/mcp.M900450-MCP200 PMC2860227

[19] Thobhani S., Yuen C. T., Bailey M. J., and Jones C. (2009) Identification and quantification of N-linked oligosaccharides released from glycoproteins: an inter-laboratory study. Glycobiology 19, 201–211 18849584 10.1093/glycob/cwn099

[20] Leymarie N., Griffin P. J., Jonscher K., Kolarich D., Orlando R., McComb M., Zaia J., Aguilan J., Alley W. R., Altmann F., Ball L. E., Basumallick L., Bazemore-Walker C. R., Behnken H., Blank M. A., Brown K. J., Bunz S. C., Cairo C. W., Cipollo J. F., Daneshfar R., Desaire H., Drake R. R., Go E. P., Goldman R., Gruber C., Halim A., Hathout Y., Hensbergen P. J., Horn D. M., Hurum D., Jabs W., Larson G., Ly M., Mann B. F., Marx K., Mechref Y., Meyer B., Möginger U., Neusüâ C., Nilsson J., Novotny M. V., Nyalwidhe J. O., Packer N. H., Pompach P., Reiz B., Resemann A., Rohrer J. S., Ruthenbeck A., Sanda M., Schulz J. M., Schweiger-Hufnagel U., Sihlbom C., Song E., Staples G. O., Suckau D., Tang H., Thaysen-Andersen M., Viner R. I., An Y., Valmu L., Wada Y., Watson M., Windwarder M., Whittal R., Wuhrer M., Zhu Y., and Zou C. (2013) Interlaboratory study on differential analysis of protein glycosylation by mass spectrometry: The ABRF Glycoprotein Research Multi-Institutional Study 2012. Mol. Cell. Proteomics 12, 2935–2951 23764502 10.1074/mcp.M113.030643 PMC3790302

[21] Reusch D., Haberger M., Maier B., Maier M., Kloseck R., Zimmermann B., Hook M., Szabo Z., Tep S., Wegstein J., Alt N., Bulau P., and Wuhrer M. (2015) Comparison of methods for the analysis of therapeutic immunoglobulin G Fc-glycosylation profiles-Part 1: Separation-based methods. Mabs 7, 167–179 25524468 10.4161/19420862.2014.986000 PMC4623496

[22] Formolo T., Ly M., Levy M., Kilpatrick L., Lute S., Phinney K., Marzilli L., Brorson K., Boyne M., Davis D., and Schiel J. (2015) Determination of the NISTmAb Primary Structure. In State-of-the-Art and Emerging Technologies for Therapeutic Monoclonal Antibody Characterization Volume 2. Biopharmaceutical Characterization: The NISTmAb Case Study (Schiel John E., Davis Darryl L., Borisov Oleg V., eds.) **Chapter 1**, pp. 1–62, American Chemical Society

[23] Schiel J. E., and Turner A. (2018) The NISTmAb Reference Material 8671 lifecycle management and quality plan. Anal. Bioanal. Chem. 410, 2067–2078 29430600 10.1007/s00216-017-0844-2 PMC5830479

[24] De Leoz M. L. A., Duewer D. L., and Stein S. E. (2017) NIST Interlaboratory Study on the Glycosylation of NISTmAb, a Monoclonal Antibody Reference Material. In NIST Internal Report (NISTIR), 8186

[25] Varki A., Cummings R. D., Esko J. D., Freeze H. H., Stanley P., Marth J. D., Bertozzi C. R., Hart G. W., and Etzler M. E. (2009) Symbol nomenclature for glycan representation. Proteomics 9, 5398–5399 19902428 10.1002/pmic.200900708 PMC2882983

[26] Ceroni A., Maass K., Geyer H., Geyer R., Dell A., and Haslam S. M. (2008) GlycoWorkbench: a tool for the computer-assisted annotation of mass spectra of glycans. J. Proteome Res. 7, 1650–1659 18311910 10.1021/pr7008252

[27] de Jong S. E., Selman M. H., Adegnika A. A., Amoah A. S., van Riet E., Kruize Y. C., Raynes J. G., Rodriguez A., Boakye D., von Mutius E., Knulst A. C., Genuneit J., Cooper P. J., Hokke C. H., Wuhrer M., and Yazdanbakhsh M. (2016) IgG1 Fc N-glycan galactosylation as a biomarker for immune activation. Sci. Rep. 6, 28207 27306703 10.1038/srep28207 PMC4910062

[28] Halim A., Westerlind U., Pett C., Schorlemer M., Ruetschi U., Brinkmalm G., Sihlbom C., Lengqvist J., Larson G., and Nilsson J. (2014) Assignment of saccharide identities through analysis of oxonium ion fragmentation profiles in LC-MS/MS of glycopeptides. J. Proteome Res. 13, 6024–6032 25358049 10.1021/pr500898r

[29] Yu J., Schorlemer M., Gomez Toledo A., Pett C., Sihlbom C., Larson G., Westerlind U., Nilsson J., and Distinctive M. S. (2016) /MS Fragmentation Pathways of Glycopeptide-Generated Oxonium Ions Provide Evidence of the Glycan Structure. Chemistry 22, 1114–1124 26663535 10.1002/chem.201503659

[30] Pompach P., Ashline D. J., Brnakova Z., Benicky J., Sanda M., and Goldman R. (2014) Protein and Site Specificity of Fucosylation in Liver-Secreted Glycoproteins. J. Proteome Res. 13, 5561–5569 25265424 10.1021/pr5005482 PMC4261953

[31] Prien J. M., Stöckmann H., Albrecht S., Martin S. M., Varatta M., Furtado M., Hosselet S., Wang M., Formolo T., Rudd P. M., and Schiel J. E. (2015) Orthogonal Technologies for NISTmAb N-Glycan Structure Elucidation and Quantitation. In State-of-the-Art and Emerging Technologies for Therapeutic Monoclonal Antibody Characterization Volume 2. Biopharmaceutical Characterization: The NISTmAb Case Study. Schiel John E., Davis Darryl L., Borisov Oleg V., eds.) **Chapter 4**, pp. 185–235, American Chemical Society

[32] Benedetti E., Pucic-Bakovic M., Keser T., Wahl A., Hassinen A., Yang J. Y., Liu L., Trbojevic-Akmacic I., Razdorov G., Stambuk J., Klariæ L., Ugrina I., Selman M. H. J., Wuhrer M., Rudan I., Polasek O., Hayward C., Grallert H., Strauch K., Peters A., Meitinger T., Gieger C., Vilaj M., Boons G. J., Moremen K. W., Ovchinnikova T., Bovin N., Kellokumpu S., Theis F. J., Lauc G., and Krumsiek J. (2017) Network inference from glycoproteomics data reveals new reactions in the IgG glycosylation pathway. Nat. Commun. 8, 1483 29133956 10.1038/s41467-017-01525-0 PMC5684356

[33] Pilobello K. T., Krishnamoorthy L., Slawek D., and Mahal L. K. (2005) Development of a lectin microarray for the rapid analysis of protein glycopatterns. ChemBioChem 6, 985–989 15798991 10.1002/cbic.200400403

[34] Duewer D. L., Kline M. C., Sharpless K. E., Thomas J. B., Gary K. T., and Sowell A. L. (1999) Micronutrients measurement quality assurance program: helping participants use interlaboratory comparison exercise results to improve their long-term measurement performance. Anal. Chem. 71, 1870–1878 10330911 10.1021/ac981074k

[35] Youden W. J. (1959) Graphical diagnosis of interlaboratory test results. Industrial Quality Control XV, 29–33

[36] Shirono K., Iwase K., Okazaki H., Yamazawa M., Shikakume K., Fukumoto N., Murakami M., Yanagisawa M., and Tsugoshi T. (2013) A study on the utilization of the Youden plot to evaluate proficiency test results. Accredit. Qual. Assur. 18, 161–174

[37] Horwitz W. (1982) Evaluation of analytical methods used for regulation of foods and drugs. Anal. Chem. 54, 67–76

[38] Thompson M., and Lowthian P. J. (1997) The Horwitz function revisited. J. AOAC. Int. 80, 676–679

