# Supplementary material for: NIST Interlaboratory Study on Glycosylation Analysis of Monoclonal Antibodies: Comparison of Results from Diverse Analytical Methods
Source: Mol Cell Proteomics. 2019 Oct 7;19(1):11–30. doi: 10.1074/mcp.RA119.001677 (PMC6944243; doi:10.1074/mcp.RA119.001677)
Supplement: Supplemental Data [file 154326_1_supp_406513_pyvm51.pdf]

# Supplementary Information

## NIST Interlaboratory Study on Glycosylation Analysis of Monoclonal Antibodies: Comparison of Results from Diverse Analytical Methods

Maria Lorna A. De Leoz[a1,a2]*, David L. Duewer[b], Adam Fung[c], Lily Liu[c], Hoi Kei Yau[c], Oscar Potter[d], Gregory O. Staples[d], Kenichiro Furuki[e], Ruth Frenkel[f], Yunli Hu[f], Zoran Sosic[f], Peiqing Zhang[g], Friedrich Altmann[h], Clemens Grùnwald-Gruber[h], Chun Shao[i], Joseph Zaia[i], Waltraud Evers[j1], Stuart Pengelley[j1], Detlev Suckau[j1], Anja Wiechmann[j1], Anja Resemann[j1], Wolfgang Jabs[j1,j2], Alain Beck[k], John W. Froehlich[l], Chuncui Huang[m], Yan Li[m], Yaming Liu[m], Shiwei Sun[n], Yaojun Wang[n], Youngsuk Seo[o], Hyun Joo An[o], Niels-Christian Reichardt[p1], Juan Echevarria Ruiz[p1,p2], Stephanie Archer-Hartmann[q], Parastoo Azadi[q], Len Bell[r], Zsuzsanna Lakos[s], Yanming An[t], John F. Cipollo[t], Maja Pucic-Bakovic[u1], Jerko Štambuk[u1], Gordan Lauc[u1,u2], Xu Li[v], Peng George Wang[v], Andreas Bock[w], René Hennig[w], Erdmann Rapp[w,aj], Marybeth Creskey[x], Terry D. Cyr[x], Miyako Nakano[y], Taiki Sugiyama[y], Pui-King Amy Leung[z], Paweł Link-Lenczowski[aa], Jolanta Jaworek[aa], Shuang Yang[ab], Hui Zhang[ab], Tim Kelly[ac1], Song Klapoetke[ac1], Rui Cao[ac1,ac2], Jin Young Kim[ad], Hyun Kyoung Lee[ad], Ju Yeon Lee[ad], Jong Shin Yoo[ad], Sa-Rang Kim[ae], Soo-Kyung Suh[ae], Noortje de Haan[af], David Falck[af], Guinevere S. M. Lageveen-Kammeijer[af], Manfred Wuhrer[af], Robert J. Emery[ag], Radoslaw P. Kozak[ag], Li Phing Liew[ag], Louise Royle[ag], Paulina A. Urbanowicz[ag], Nicolle H. Packer[ah1], Xiaomin Song[ah1], Arun Everest-Dass[ah1,ah2], Erika Lattová[ai], Samanta Cajic[aj], Kathirvel Alagesan[ak], Daniel Kolarich[ak], Toyin Kasali[al], Viv Lindo[al], Yuetian Chen[am], Kudrat Goswami[am], Brian Gau[an], Ravi Amunugama[ao], Richard Jones[ao], Corné J.M. Stroop[ap], Koichi Kato[aq,ar], Hirokazu Yagi[ar], Sachiko Kondo[ar,as], CT Yuen[at], Akira Harazono[au], Xiaofeng Shi[av], Paula E. Magnelli[av], Brian T. Kasper[aw1], Lara Mahal[aw1,aw2], David J. Harvey[ax], Roisin O'Flaherty[ay], Pauline M. Rudd[ay], Radka Saldova[ay], Elizabeth S. Hecht[az], David C. Muddiman[az], Jichao Kang[ba], Prachi Bhoskar[bb], Daniele Menard[bb], Andrew Saati[bb], Christine Merle[bc], Steven Mast[bd], Sam Tep[bd], Jennie Truong[bd], Takashi Nishikaze[be], Sadanori Sekiya[be], Aaron Shafer[bf], Sohei Funaoka[bg], Masaaki Toyoda[bg], Peter de Vreugd[bh], Cassie Caron[bi], Pralima Pradhan[bi], Niclas Chiang Tan[bi], Yehia Mechref[bj], Sachin Patil[bk], Jeffrey S. Rohrer[bk], Ranjan Chakrabarti[bl1], Disha Dadke[bl1,bl2], Mohammedazam Lahori[bl1,bl3], Chunxia Zou[bm1,bm2], Christopher Cairo[bm1,bm2], Béla Reiz[bm2], Randy M. Whittal[bm2], Carlito B. Lebrilla[bn], Lauren Wu[bn], Andras Guttman[bo1], Marton Szigeti[bo1,bo2], Benjamin G. Kremkow[bp], Kelvin H. Lee[bp], Carina Sihlbom[bq], Barbara Adamczyk[br], Chunsheng Jin[br], Niclas G. Karlsson[br], Jessica Örnros[br], Göran Larson[bs], Jonas Nilsson[bs], Bernd Meyer[bt], Alena Wiegandt[bt], Emy Komatsu[bu], Helene Perreault[bu], Edward D. Bodnar[bu,d], Nassur Said[bv], Yannis-Nicolas Francois[bv], Emmanuelle Leize-Wagner[bv], Sandra Maier[bw], Anne Zeck[bw], Albert J. R. Heck[bx1], Yang Yang[bx1,bx2], Rob Haselberg[by], Ying Qing Yu[bz1], William Alley[bz1,bz2], Joseph W Leone[ca], Hua Yuan[ca], Stephen E. Stein[a1]

* Corresponding Author: Maria Lorna A. De Leoz, Tel: +1 (240) 630-2726, lornadeleoz@gmail.com, http://orcid.org/0000-0001-9846-5524

[a1] *Mass Spectrometry Data Center, Biomolecular Measurement Division, Material Measurement Laboratory, National Institute of Standards and Technology, 100 Bureau Drive Gaithersburg, MD 20899 USA*

[a2] *Current Address: Agilent Technologies, Inc., 2500 Regency Parkway, Cary, NC 27518 USA*

[b] *Chemical Sciences Division, Material Measurement Laboratory, National Institute of Standards and Technology, 100 Bureau Drive Gaithersburg, MD 20899 USA*

[c] *Analytical Development, Agensys, Inc., 1800 Steward Street Santa Monica, CA 90404 USA*

[d] *Agilent Technologies, Inc., 5301 Stevens Creek Blvd Santa Clara, CA 95051 USA*

[e] *Astellas Pharma, 5-2-3 Tokodai, Tsukiba, Ibaraki, 300-2698, JAPAN*

[f] *Analytical Development, Biogen, 14 Cambridge Center Cambridge, MA 02142 USA*

[g] *Bioprocessing Technology Institute, 20 Biopolis Way, Level 3 Singapore 138668*

[h] *Department of Chemistry, University of Natural Resources and Life Science, Vienna (BOKU), Muthgasse 18 1190 Wien, Austria*
[i] *Center for Biomedical Mass Spectrometry, Boston University School of Medicine, 670 Albany Street Boston, MA 02118 USA*
[j1] *Bruker Daltonik GmbH, Fahrenheitstr. 4, 28359 Bremen, Germany*
[j2] *Department of Life Sciences & Technology, Beuth Hochschule für Technik Berlin, Seestraße 64, 13347 Berlin, Germany*
[k] *Centre d'Immunologie Pierre Fabre, 5 Avenue Napoléon III, BP 60497, 74164 St Julien-en-Genevois, France*
[l] *Department of Urology, Boston Children's Hospital, 300 Longwood Avenue Boston MA 02115 USA*
[m] *Institute of Biophysics, Chinese Academy of Sciences, 15 Da Tun Road, Chaoyang District, Beijing 100101 China*
[n] *Key Lab of Intelligent Information Processing, Institute of Computing Technology, Chinese Academy of Sciences, 15 Da Tun Road, Chaoyang District, Beijing 100101 China*
[o] *Graduate School of Analytical Science and Technology, Chungnam National University, Gung-dong 220, Yuseong-Gu, Daejeon 305-764, Korea (South)*
[p1] *CICbiomaGUNE, Paseo Miramon 182, 20009 San Sebastian, Spain*
[p2] *Current address: Asparia Glycomics, Paseo de Mikeletegi, 8320009 San Sebastián, Spain*
[q] *Analytical Services, Complex Carbohydrate Research Center, University of Georgia, 315 Riverbend Road Athens, GA 30602 USA*
[r] *BioCMC Solutions (Large Molecules), Covance Laboratories Limited, Otley Road, Harrogate, North Yorks HG3 1PY, United Kingdom*
[s] *Biochemistry Method Development & Validation, Eurofins Lancaster Laboratories, Inc., 2425 New Holland Pike Lancaster, PA 17601 USA*
[t] *Center for Biologics Evaluation and Research, Food and Drug Administration, 10903 New Hampshire Avenue, Silver Spring, MD 20993 USA*
[u1] *Glycoscience Research Laboratory, Genos, Borongajska cesta 83h, 10 000 Zagreb, Croatia*
[u2] *Faculty of Pharmacy and Biochemistry, University of Zagreb, A. Kovačića 1, 10 000 Zagreb, Croatia*
[v] *Department of Chemistry, Georgia State University, 100 Piedmont Avenue, Atlanta, GA 30303 USA*
[w] *glyXera GmbH, Brenneckestrasse 20 * ZENIT / 39120 Magdeburg, Germany*
[x] *Health Products and Foods Branch, Health Canada, AL 2201E, 251 Sir Frederick Banting Driveway, Ottawa, Ontario, K1A 0K9 Canada*
[y] *Graduate School of Advanced Sciences of Matter, Hiroshima University, 1-3-1 Kagamiyama Higashi-Hiroshima 739-8530 Japan*
[z] *ImmunoGen, 830 Winter Street, Waltham, MA 02451 USA*
[aa] *Department of Medical Physiology, Jagiellonian University Medical College, ul. Michalowskiego 12, 31-126 Krakow, Poland*
[ab] *Department of Pathology, Johns Hopkins University, 400 N. Broadway Street Baltimore, MD 21287 USA*
[ac1] *Mass Spec Core Facility, KBI Biopharma, 1101 Hamlin Road Durham, NC 27704 USA*
[ac2] *Current address: Analytical Development-Protein Characterization, Janssen R & D, LLC, 260 Great Valley Parkway, Malvern, PA, 19355, USA*
[ad] *Division of Mass Spectrometry, Korea Basic Science Institute, 162 YeonGuDanji-Ro, Ochang-eup, Cheongwon-gu, Cheongju Chungbuk, 363-883 Korea (South)*
[ae] *Advanced Therapy Products Research Division, Korea National Institute of Food and Drug Safety, 187 Osongsaengmyeong 2-ro Osong-eup, Heungdeok-gu, Cheongju-si, Chungcheongbuk-do, 363-700, Korea (South)*
[af] *Center for Proteomics and Metabolomics, Leiden University Medical Center, P.O. Box 9600, 2300 RC Leiden, The Netherlands*
[ag] *Ludger Limited, Culham Science Centre, Abingdon, Oxfordshire, OX14 3EB, United Kingdom*
[ai1] *Biomolecular Discovery and Design Research Centre and ARC Centre of Excellence for Nanoscale BioPhotonics (CNBP), Macquarie University, North Ryde, Australia*
[ai2] *Current address: Institute for Glycomics, Griffith University, Southport, Australia*
[ai] *Proteomics, Central European Institute for Technology, Masaryk University, Kamenice 5, A26, 625 00 BRNO, Czech Republic*
[aj] *Max Planck Institute for Dynamics of Complex Technical Systems, Sandtorstrasse 1, 39106 Magdeburg, Germany*

[ak] *Department of Biomolecular Sciences, Max Planck Institute of Colloids and Interfaces, 14424 Potsdam, Germany; Current address: Institute for Glycomics, Griffith University, Gold Coast Campus, Queensland 4222, Australia*
[al] *AstraZeneca, Granta Park, Cambridgeshire, CB21 6GH United Kingdom*
[am] *Merck, 2015 Galloping Hill Rd, Kenilworth, NJ 07033 USA*
[an] *Analytical R&D, MilliporeSigma, 2909 Laclede Ave. St. Louis, MO 63103 USA; Current address: Pfizer 700 Chesterfield Pkwy W, St. Louis, MO 63198 US*
[ao] *MS Bioworks, LLC, 3950 Varsity Drive Ann Arbor, MI 48108 USA*
[ap] *MSD, Molenstraat 110, 5342 CC Oss, The Netherlands*
[aq] *Exploratory Research Center on Life and Living Systems (ExCELLS), National Institutes of Natural Sciences, 5 -1 Higashiyama, Myodaiji, Okazaki 444-8787 Japan*
[ar] *Graduate School of Pharmaceutical Sciences, Nagoya City University, 3-1 Tanabe-dori, Mizuhoku, Nagoya 467-8603 Japan*
[as] *Medical & Biological Laboratories Co., Ltd, 2-22-8 Chikusa, Chikusa-ku, Nagoya 464-0858 Japan*
[at] *National Institute for Biological Standards and Control, Blanche Lane, South Mimms, Potters Bar, Hertfordshire EN6 3QG United Kingdom*
[au] *Division of Biological Chemistry & Biologicals, National Institute of Health Sciences, 1-18-1 Kamiyoga, Setagaya-ku, Tokyo 158-8501 Japan*
[av] *New England Biolabs, Inc., 240 County Road, Ipswich, MA 01938 USA*
[aw1] *New York University, 100 Washington Square East New York City, NY 10003 USA*
[aw2] *Current Address: Department of Chemistry, University of Alberta, 11227 Saskatchewan Drive, Edmonton, Alberta, Canada T6G 2G2*
[ax] *Target Discovery Institute, Nuffield Department of Medicine, University of Oxford, Roosevelt Drive, Oxford, OX3 7FZ, UK*
[ay] *GlycoScience Group, The National Institute for Bioprocessing Research and Training, Fosters Avenue, Mount Merrion, Blackrock, Co. Dublin, Ireland*
[az] *Department of Chemistry, North Carolina State University, 2620 Yarborough Drive Raleigh, NC 27695 USA*
[ba] *Pantheon, 201 College Road East Princeton, NJ 08540 USA*
[bb] *Pfizer Inc., 1 Burtt Road Andover, MA 01810, USA*
[bc] *Proteodynamics, ZI La Varenne 20-22 rue Henri et Gilberte Goudier 63200 RIOM, France*
[bd] *ProZyme, Inc., 3832 Bay Center Place Hayward, CA 94545 USA*
[be] *Koichi Tanaka Mass Spectrometry Research Laboratory, Shimadzu Corporation, 1 Nishinokyo Kuwabara-cho Nakagyo-ku, Kyoto, 604 8511 Japan*
[bf] *Children's GMP LLC, St. Jude Children's Research Hospital, 262 Danny Thomas Place Memphis, TN 38105 USA*
[bg] *Sumitomo Bakelite Co., Ltd., 1-5 Muromati 1-Chome, Nishiku, Kobe, 651-2241 Japan*
[bh] *Synthon Biopharmaceuticals, Microweg 22 P.O. Box 7071, 6503 GN Nijmegen, The Netherlands*
[bi] *Takeda Pharmaceuticals International Co., 40 Landsdowne Street Cambridge, MA 02139 USA*
[bj] *Department of Chemistry and Biochemistry, Texas Tech University, 2500 Broadway, Lubbock, TX 79409 USA*
[bk] *Thermo Fisher Scientific, 1214 Oakmead Parkway Sunnyvale, CA 94085 USA*
[bl1] *United States Pharmacopeia India Pvt. Ltd. IKP Knowledge Park, Genome Valley, Shamirpet, Turkapally Village, Medchal District, Hyderabad 500 101 Telangana, India*
[bl2] *Current Address: Analytical, Regulatory, and Clinical Sciences, Aurobindo Biologics ( A Division of Aurobindo Pharma Ltd.), Indrakaran Village, Sangareddy District - 502329, Telangana, India*
[bl3] *Current Address: Translational Science, Intas Pharmaceuticals Ltd., Plot No. 423/P/A, Sarkhej-Bavla Highway, Moraiya, Ta; Sanand, Ahmedabad 382213 Gujrat India*
[bm1] *Alberta Glycomics Centre, University of Alberta, Edmonton, Alberta T6G 2G2 Canada*
[bm2] *Department of Chemistry, University of Alberta, Edmonton, Alberta T6G 2G2 Canada*
[bn] *Department of Chemistry, University of California, One Shields Ave, Davis, CA 95616 USA*
[bo1] *Horváth Csaba Memorial Laboratory for Bioseparation Sciences, Research Center for Molecular Medicine, Doctoral School of Molecular Medicine, Faculty of Medicine, University of Debrecen, Debrecen, Egyetem ter 1, Hungary*
[bo2] *Translational Glycomics Research Group, Research Institute of Biomolecular and Chemical Engineering, University of Pannonia, Veszprem, Egyetem ut 10, Hungary*

[bp] *Delaware Biotechnology Institute, University of Delaware, 15 Innovation Way Newark, DE 19711 USA*
[bq] *Proteomics Core Facility, University of Gothenburg, Medicinaregatan 1G SE 41390 Gothenburg, Sweden*
[br] *Department of Medical Biochemistry and Cell Biology, University of Gothenburg, Institute of Biomedicine, Sahlgrenska Academy, Medicinaregatan 9A, Box 440, 405 30, Gothenburg, Sweden*
[bs] *Department of Clinical Chemistry and Transfusion Medicine, Sahlgrenska Academy at the University of Gothenburg, Bruna Straket 16, 41345 Gothenburg, Sweden*
[bt] *Department of Chemistry, University of Hamburg, Martin Luther King Pl. 6 20146 Hamburg, Germany*
[bu] *Department of Chemistry, University of Manitoba, 144 Dysart Road, Winnipeg, Manitoba, Canada R3T 2N2*
[bv] *Laboratory of Mass Spectrometry of Interactions and Systems, University of Strasbourg, UMR Unistra-CNRS 7140, France*
[bw] *Natural and Medical Sciences Institute, University of Tübingen, Markwiesenstraße 55, 72770 Reutlingen, Germany*
[bx1] *Bijvoet Center for Biomolecular Research and Utrecht Institute for Pharmaceutical Sciences, Utrecht University, Padualaan 8, 3584 CH Utrecht, The Netherlands*
[bx2] *Current Address: Novartis Biologics Center, Novartis Institutes for BioMedical Research, Inc. Novartis Pharma, 4002 Basel, Switzerland*
[by] *Division of Bioanalytical Chemistry, Amsterdam Institute for Molecules, Medicines and Systems, Vrije Universiteit Amsterdam, de Boelelaan 1085, 1081 HV Amsterdam, The Netherlands*
[bz1] *Department of Chemistry, Waters Corporation, 34 Maple Street Milford, MA 01757 USA*
[bz2] *Current Address: Department of Science and Mathematics, Texas A&M University-San Antonio, One University Way, San Antonio, Texas 78224*
[ca] *Zoetis, 333 Portage St. Kalamazoo, MI 49007 USA*

# Table of Contents

**Supplementary Discussion 1.** Study Rationale and Design ........ 6

**Supplementary Discussion 2.** Rationale for the Samples ........ 7

**Supplementary Figure 1.** Participating laboratories. ........ 8

**Supplementary Figure 2.** Reversed-phase (C18) LC-MS/MS spectra of glycopeptides. ........ 9

**Supplementary Figure 3.** Number of samples reported Vs sum of relative abundances per sample. ........ 10

**Supplementary Figure 4.** Limit of reporting for one set of results. ........ 11

**Supplementary Figure 5.** Limit of reporting versus minimum reported value for one set of results. ........ 12

**Supplementary Figure 6.** Youden two-sample plots, Compositions 1 to 12. ........ 13

**Supplementary Figure 6, Continued.** Youden two-sample plots, Compositions 13 to 24. ........ 14

**Supplementary Figure 6, Continued.** Youden two-sample plots, Compositions 25 to 36. ........ 15

**Supplementary Figure 6, Continued.** Youden two-sample plots, Compositions 37 to 48. ........ 16

**Supplementary Figure 6, Continued.** Youden two-sample plots, Compositions 49 to 59. ........ 17

**Supplementary Table 1.** Analytical approaches used by laboratories in this study. ........ 18

**Supplementary Table 2.** Identified glycans. ........ 19

**Supplementary Table 3.** Summary of Reported and Derived Values ........ 36

**Legend for Supplementary Table 3.** ........ 47

**Supplementary Table 4.** Reported advantages and limitations of select methods as described by the participants. ........ 48

**Supplementary Discussion 1.** Study Rationale and Design

Prior to the full-scale study, several laboratories with broad experience in glycosylation analysis performed a preliminary "Stage 1" series of analyses. This step enabled the refinement of reports, verified that no problems existed in sample preparation and shipping, and generated helpful suggestions on how to improve the study. Data and method reporting templates[24] were modified based on feedback from these experienced laboratories. For example, sixteen glycan compositions were initially listed in the data reporting template but after Stage 1, 68 *N*-glycan entries consisting of 54 unique compositions were listed. Also, both Consortium for Functional Glycomics (CFG) and Oxford (UOXF) names and structures were listed in the final template.

Because of differences in method details, several reporting options were allowed. To accommodate laboratories that could only identify compositions and those that could separate isomers, glycan compositions were listed in the data reporting template as main glycans then isomers under them.

There is no current standard to denote monosaccharide compositions in *N*-glycans. As described in Methods, a new notation was devised. This notation has been adapted for use in the NIST glycan tandem MS library (www.chemdata.nist.gov/glycan).

Participants were asked to use their method of choice to determine the relative glycan content in the two samples. Some participants provided multiple reports describing results of different methods. Common methods were pre-filled in drop down menus of the method reporting template. Participants could write comments or add other methods. Laboratories were requested to create separate reports for each method of analysis.

Data were analyzed using a variety of robust data analysis techniques to assess measurement reproducibility and to characterize glycan distributions. Results were compiled and evaluated for the community's consensus medians, within-laboratory precision, and concordance within the laboratories. A technical summary[24] of reported and derived values from all laboratories, a table of all identified glycans, and an individualized graphical analysis of their performance for the exercise were sent to participants. Appropriate modifications were made as necessary.

**Supplementary Discussion 2.** Rationale for the Samples

Samples sent to participants were intact mAbs rather than cleaved glycans to permit all varieties of glycosylation determination, including protein digestion and intact protein analysis. Our goal was to assess net measurement variability starting with a given protein sample, as done when comparing innovator and biosimilar drugs.

As outlined in Materials and Methods, two samples were sent to laboratories, namely NISTmAb and a modified-NISTmAb. Measurements on two related samples helped to assess within-lab and between-lab errors using Youden two-sample plots.[25, 26] This scheme is a simple technique that plots, for a given glycan, measurement results for one sample against those of the second. The spread among the left-to-right diagonal relates to systematic between-participant bias; the off-axis distance from this diagonal relates to within-participant variability.

NISTmAb was chosen as the primary sample because it is a well-characterized reference material[31] readily available and now widely distributed to researchers. Around 1500 vials of NISTmAb were sold as of November 2019. Choosing a second sample was a challenge. An ideal second sample would be a monoclonal antibody that is not greatly different than the NISTmAb. Techniques such as chemoenzymatic glycosylation remodeling and modification by sialyltransferases (α2-6), galactosyltransferases, fucosidases (α1-2,4,6), neuraminidases, and galactosidases (β1-4, β1-4,6, and α1-3,6) were explored to produce a second sample. Some of the techniques produced complex mixtures that were difficult to purify or produced too little of the product.

Use of β1,4-galactosidase partly addressed the challenge. This enzyme breaks the glycosidic bond of the terminal β1,4-galactose residue in a glycan. However, a complete reaction using this exoglycosidase entirely eliminates certain glycans such as G1F and G2F, leading to drastic differences from the NISTmAb. To circumvent this issue, β1,4-galactosidase-treated NISTmAb was added back to the original NISTmAb sample in a 30:70 ratio. The resulting solution was used as the second sample for the study, referred to as mod-NISTmAb.

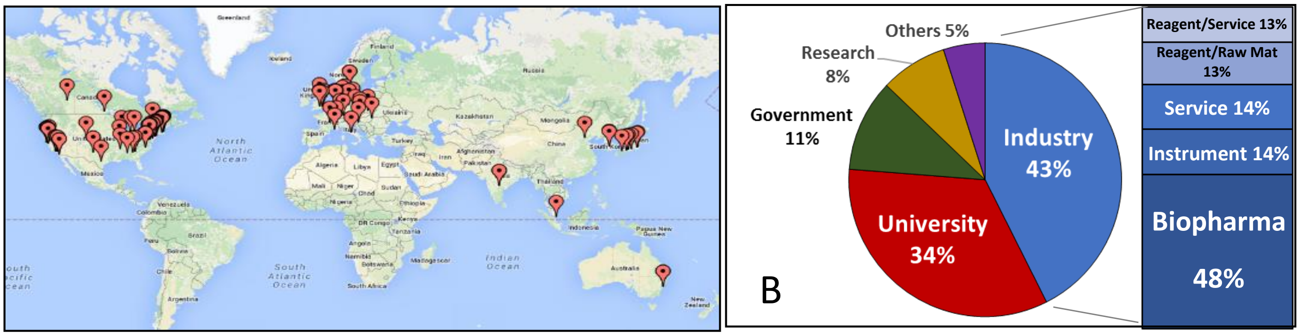


**Supplementary Figure 1.** Participating laboratories.

Laboratories are identified by A) location and B) laboratory type, with breakdown of industry laboratories.

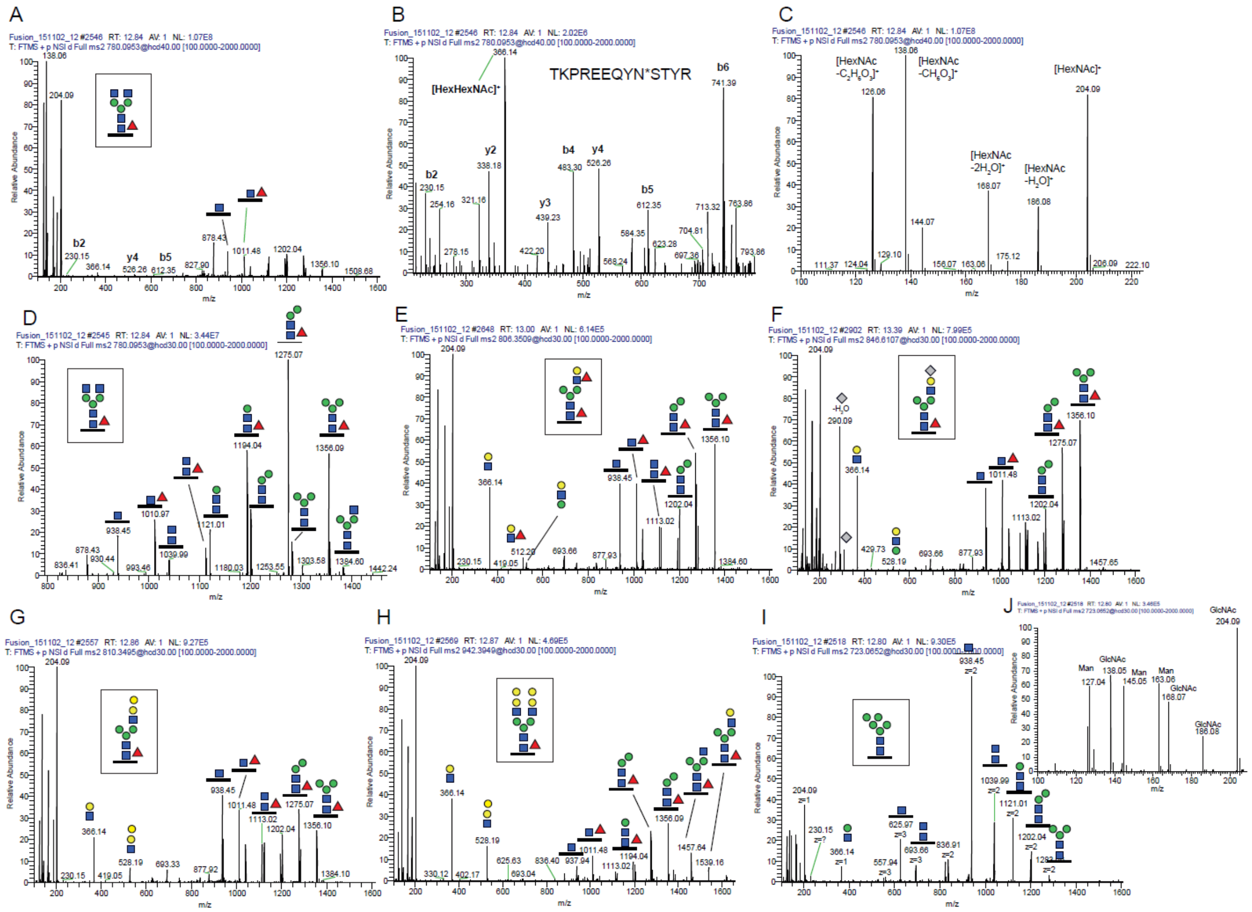


**Supplementary Figure 2.** Reversed-phase (C18) LC-MS/MS spectra of glycopeptides.

*N*-Glycopeptides were identified by MS mass and MS/MS fragmentation data and quantified by peak area. A) Full HCD spectrum of G0F at 40% normalized collision energy. The precursor ion is shown boxed. A magnification of Spectrum A at B) *m/z* 200 to *m/z* 800; C) *m/z* 100 to *m/z* 225; and D) *m/z* 800 to *m/z* 1400. MS/MS spectra of example glycan motifs: E) antenna fucosylation; F) NeuGc; G) H) 2Hex1GlcNAc, e.g. GalGalGlcNAc; and I), J) high mannose. Data courtesy of a participant.

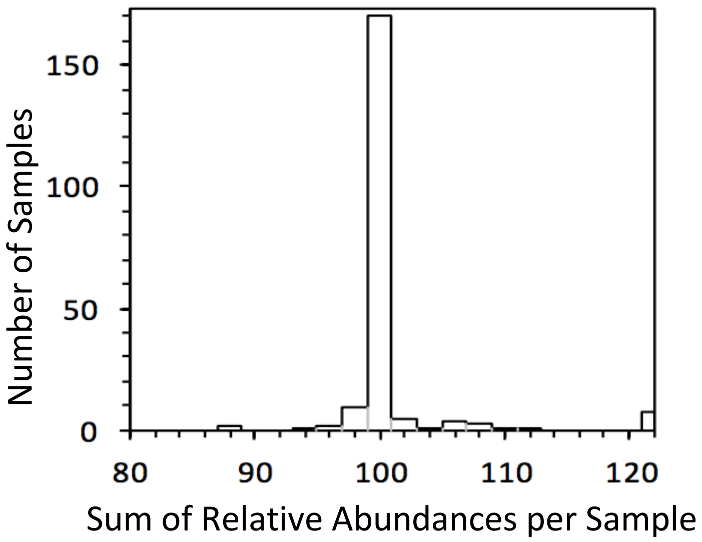


**Supplementary Figure 3.** Number of samples reported Vs sum of relative abundances per sample.

Participants were requested to normalize their NISTmAb and mod-NISTmAb results to the sum of all glycans reported in each sample. Of the 206 samples, 170 were fully normalized.

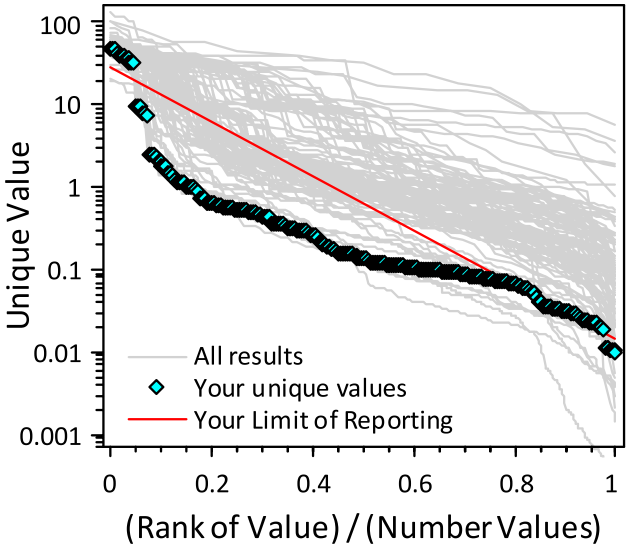


**Supplementary Figure 4.** Limit of reporting for one set of results.

Gray lines are traces of the unique non-zero values reported in each set of results, where the values are ordered by decreasing value. The graph shows values, in blue diamond, of one participant.

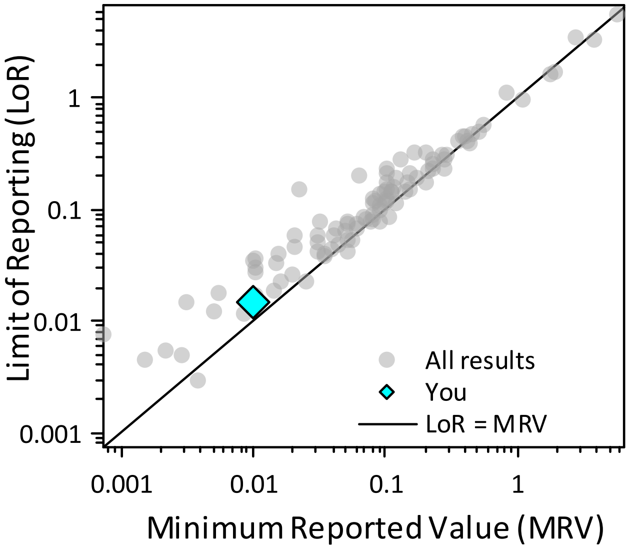


**Supplementary Figure 5.** Limit of reporting versus minimum reported value for one set of results. The graph shows the data point, in blue diamond, of one participant.

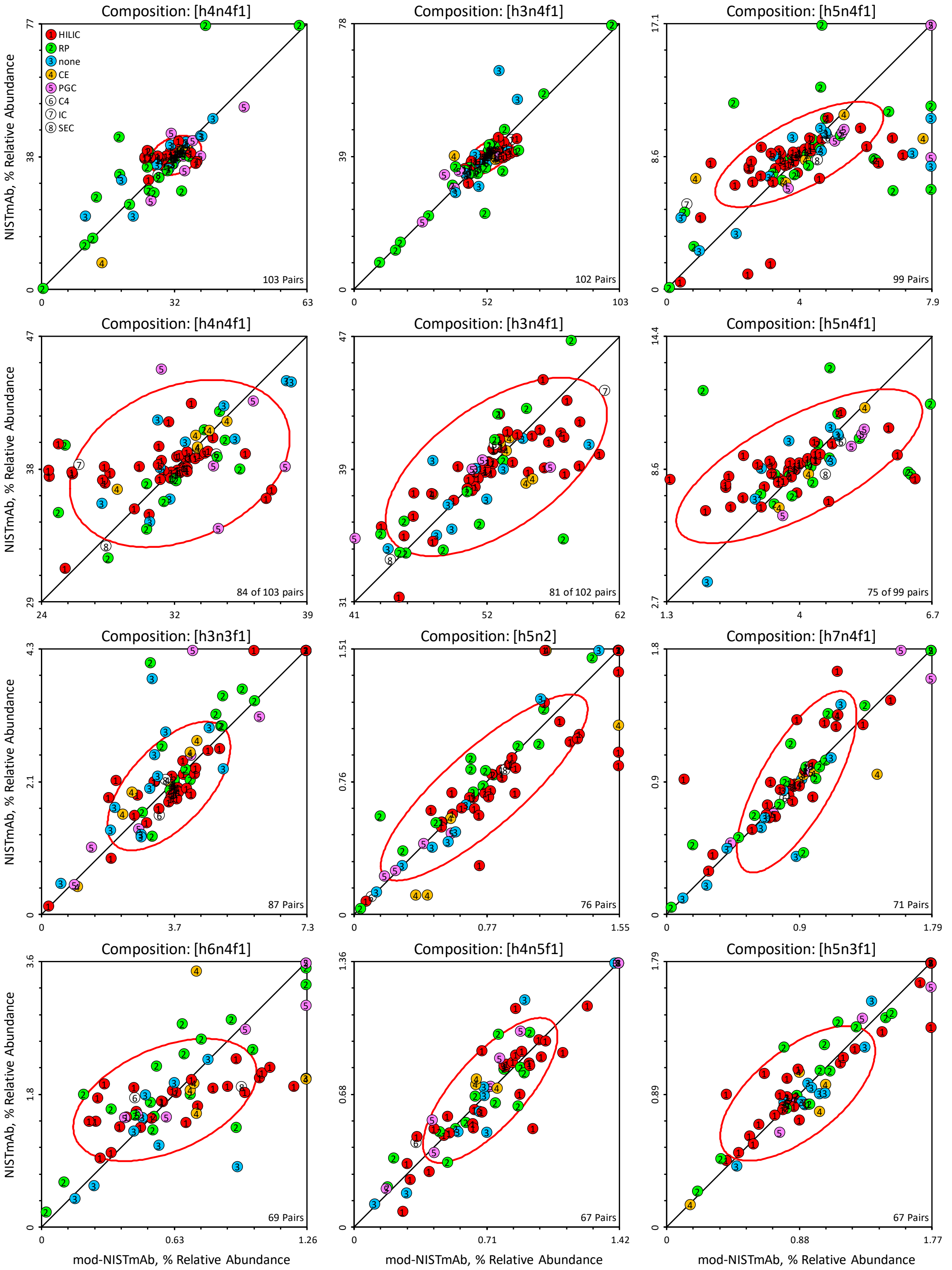


**Supplementary Figure 6.** Youden two-sample plots, Compositions 1 to 12.

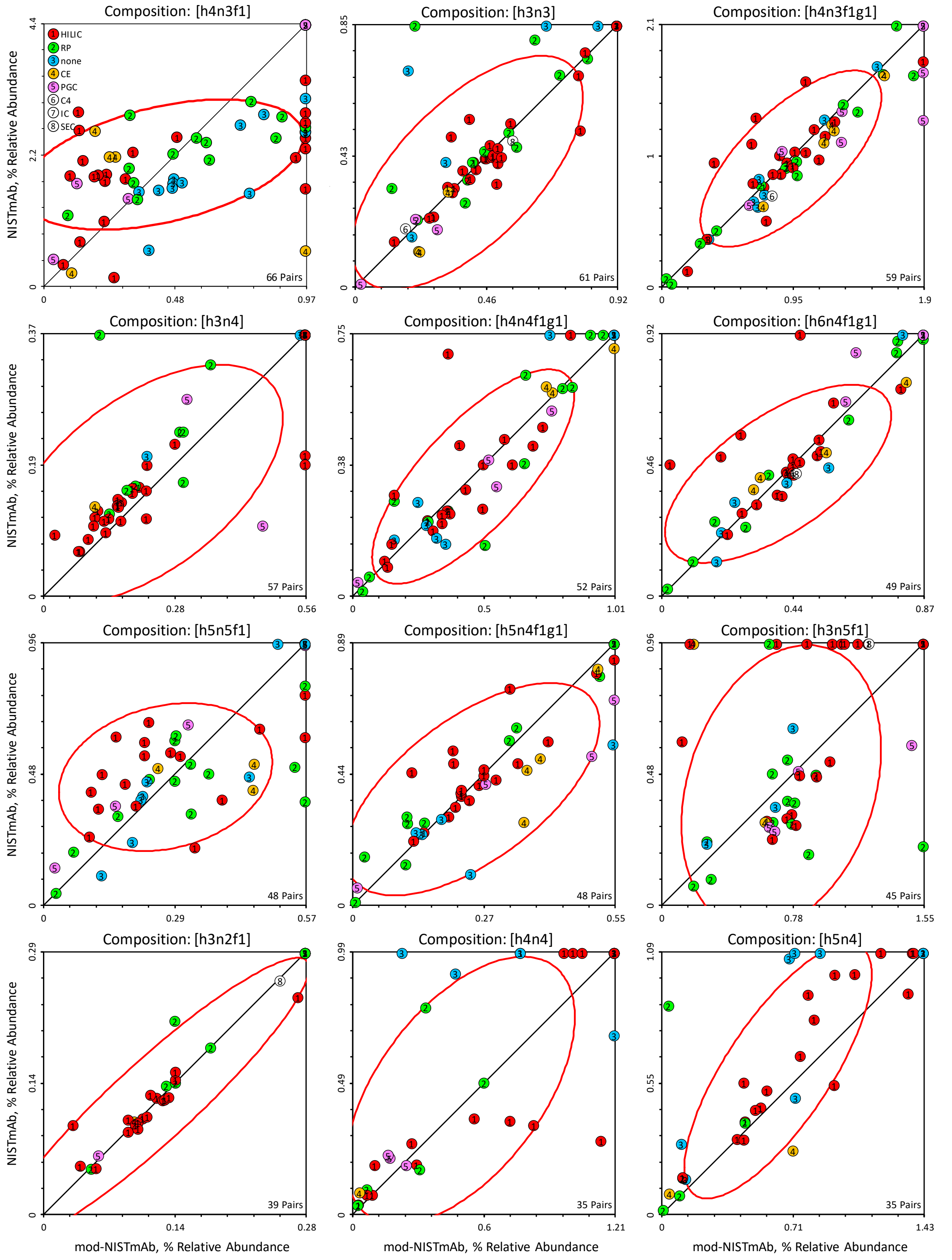


**Supplementary Figure 6, Continued.** Youden two-sample plots, Compositions 13 to 24.

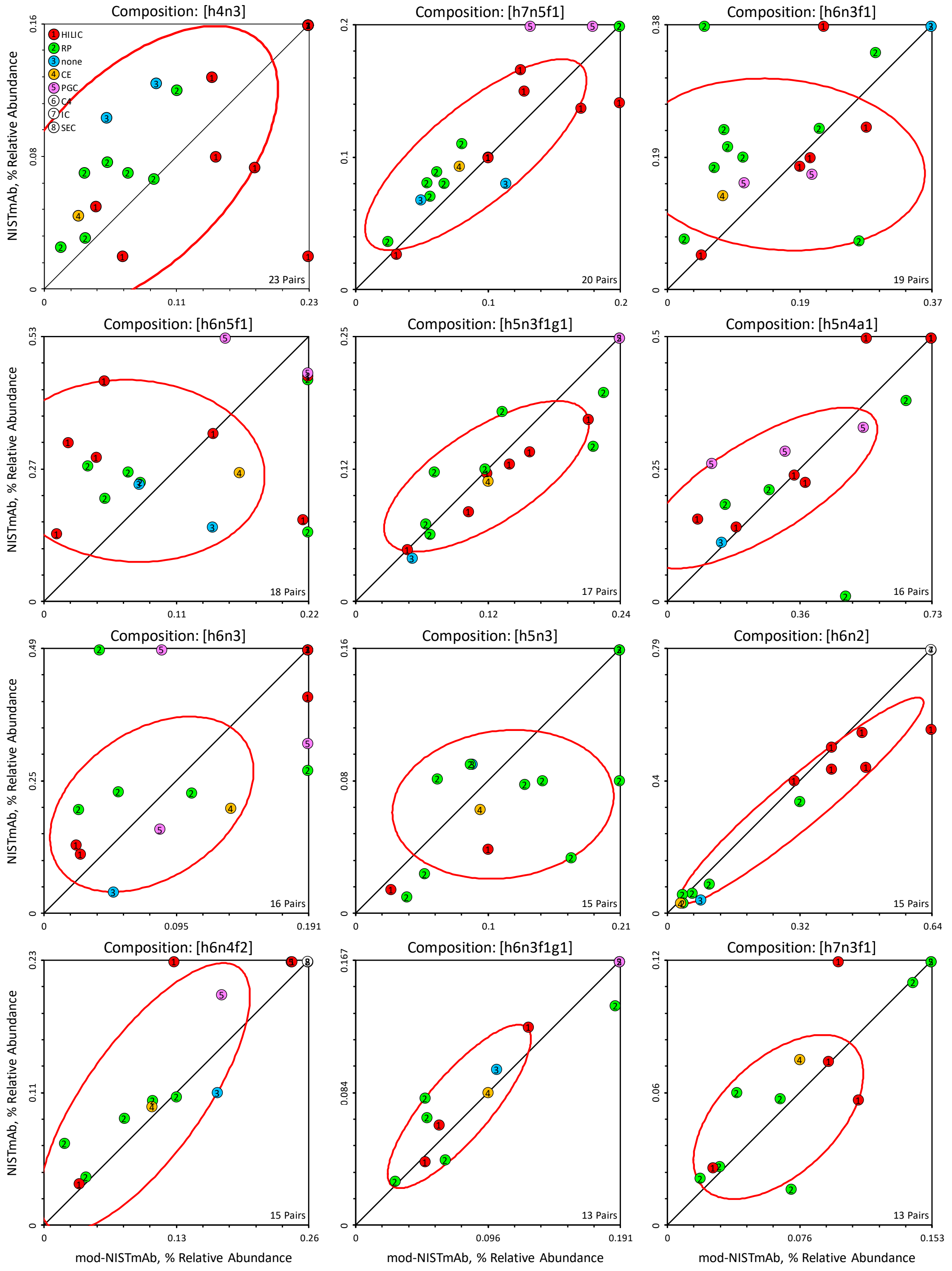


**Supplementary Figure 6, Continued.** Youden two-sample plots, Compositions 25 to 36.

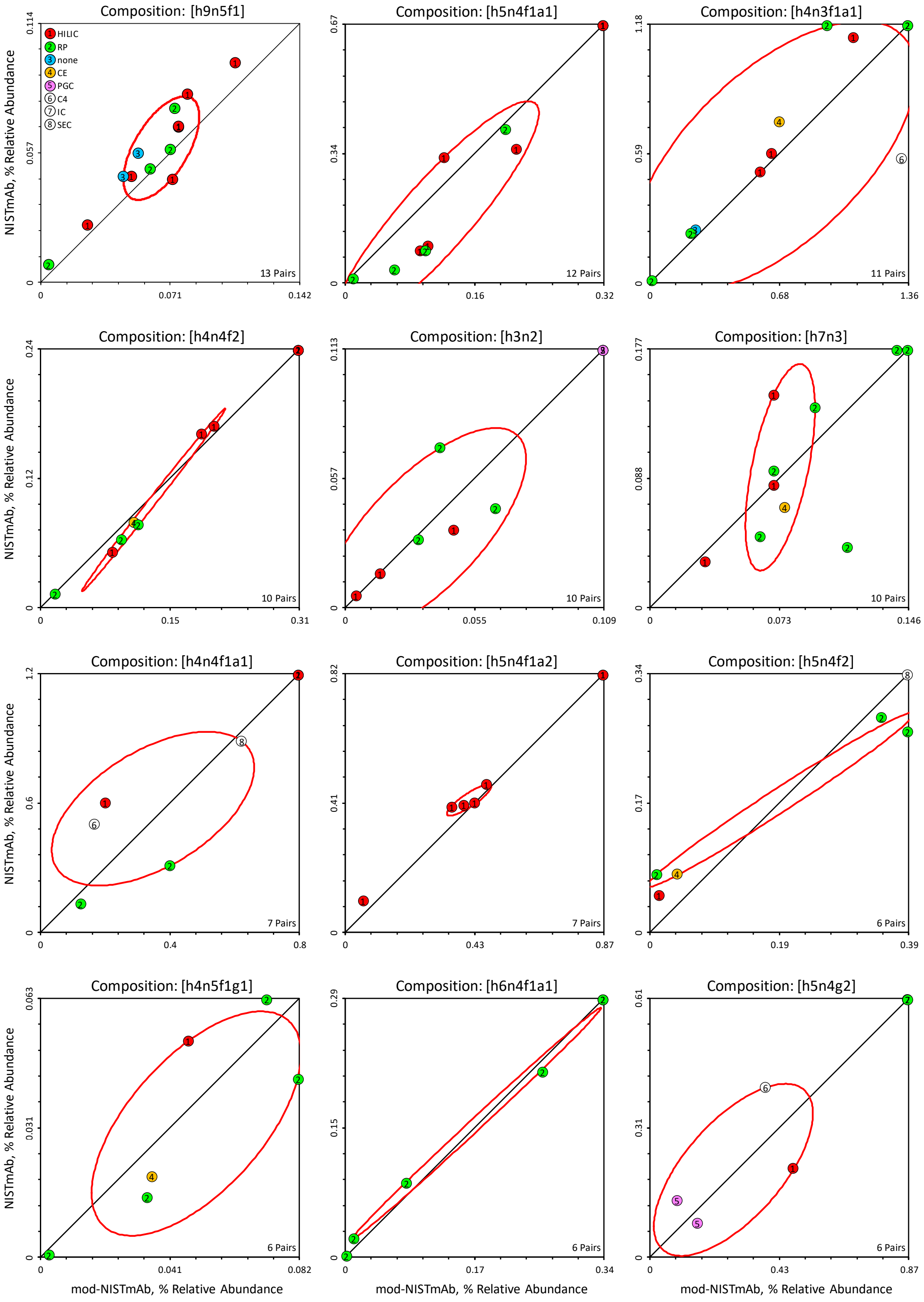


**Supplementary Figure 6, Continued.** Youden two-sample plots, Compositions 37 to 48.

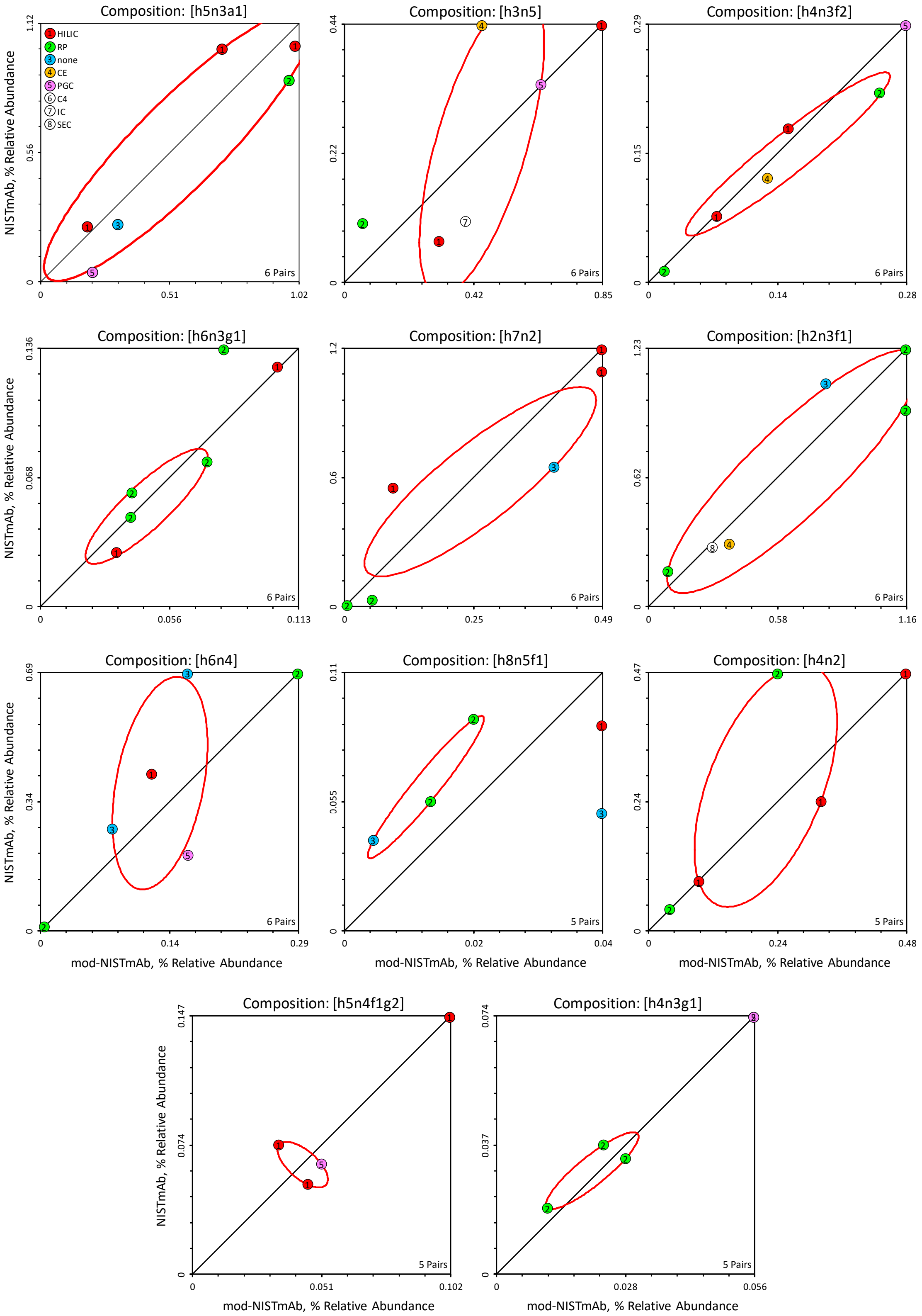


**Supplementary Figure 6, Continued.** Youden two-sample plots, Compositions 49 to 59.

**Supplementary Table 1.** Analytical approaches used by laboratories in this study.

Numbers indicate number of participants.

| Analyte / Lab Sector[a] | Industry | | University | Other Sector[c] |
|---|---|---|---|---|
| | Biopharma | Other Industry[b] | | |
| Glycan | **19** | **20** | **18** | **11** |
| Fluorescent Labelling | **19** | **16** | **6** | **7** |
| 2-AB | 7 | 3 | 3 | 5 |
| Glycosylamine | 9 | 6 | 0 | 0 |
| Other fluorescent[d] | 3 | 7 | 3 | 2 |
| Non-fluorescent[e] | **0** | **4** | **12** | **4** |
| Glycopeptide | **2** | **3** | **14** | **1** |

a: not enough data for other analytes (protein fragment and intact protein)

b: government, research, hospital, standard setting organization laboratories

c: instrument, service, reagent/raw mat, reagent/service laboratories

d: APTS, procainamide, 2-AA, 2-aminopyridine, 4-AA labeling

e: permethylation, reduction, none, ethyl esterification, INLIGHT, phenylhydrazine, p-toluidine derivatization

**Supplementary Table 2.** Identified glycans.

Glycans reported by participants arranged in increasing monosaccharide composition[e]. Entries highlighted in orange are unique glycan compositions; glycans having the same monosaccharide compositions are shown beneath the highlighted entry. Structures were drawn as reported; some linkages are incomplete, and some structures may not be feasible.

| Index[a] | Code[b] | Measurand[c] | Oxford[d] | Composition[e] | CFG[f] | Oxford[g] |
|---|---|---|---|---|---|---|
| 42 | | [h2n3f1] | | [h2n3f1] | | |
| 42.1 | | Fragment Man2F+N | FM2A1 | [h2n3f1] | 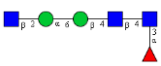 | 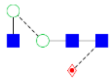 |
| 37 | | [h3n2] | | [h3n2] | | |
| 37.1 | 53 | Man3 | M3 | [h3n2] | 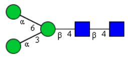 | 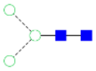 |
| 19 | | [h3n2f1] | | [h3n2f1] | | |
| 19.1 | 54 | Man3F | F(6)M3 | [h3n2f1] | 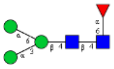 | 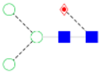 |
| 11 | | [h3n3] | | [h3n3] | | |
| 11.1 | 21 | G0-N | A1 | [h3n3] | 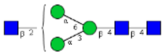 | 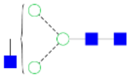 |
| 11.11 | 21a | G0-N[6] | A1[6] | [h3n3] | 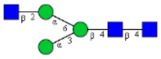 | 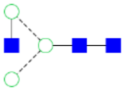 |
| 11.12 | 21b | G0-N[3] | A1[3] | [h3n3] | 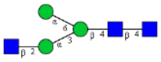 | 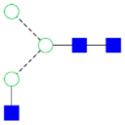 |
| 11.2 | | Man3B | M3B | [h3n3] | 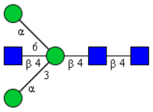 | 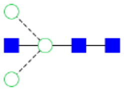 |
| 4 | | [h3n3f1] | | [h3n3f1] | | |
| 4.1 | 04 | G0F-N | F(6)A1 | [h3n3f1] | 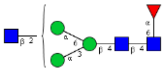 | 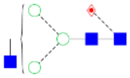 |
| 4.11 | 04a | G0F-N[6] | F(6)A1[6] | [h3n3f1] | 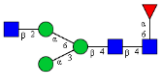 | 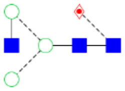 |
| 4.12 | 04b | G0F-N[3] | F(6)A1[3] | [h3n3f1] | 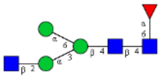 | 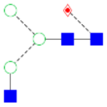 |
| 109 | | [h3n3f2] | | [h3n3f2] | | |

| Index[a] | Code[b] | Measurand[c] | Oxford[d] | Composition[e] | CFG[f] | Oxford[g] |
|---|---|---|---|---|---|---|
| 109.1 | | G0F2-N[3] | F(6)A1[3]F(3) | [h3n3f2] | 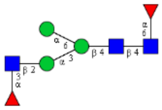 | 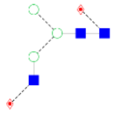 |
| 78 | | [h3n3g1] | | [h3n3g1] | | |
| 13 | | [h3n4] | | [h3n4] | | |
| 13.1 | 18 | G0 | A2 | [h3n4] | 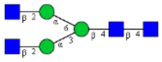 | 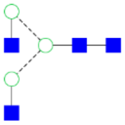 |
| 13.2 | | G0B-N | A1B | [h3n4] | 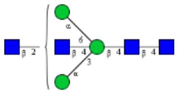 | 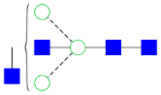 |
| 13.21 | | G0B-N[3] | A1[3]B | [h3n4] | 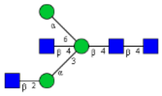 | 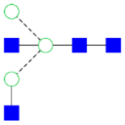 |
| 13.22 | | G0B-N[6] | A1[6]B | [h3n4] | 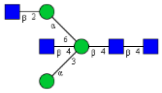 | 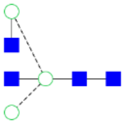 |
| 2 | | [h3n4f1] | | [h3n4f1] | | |
| 2.1 | 01 | G0F | F(6)A2 | [h3n4f1] | 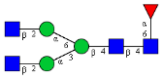 | 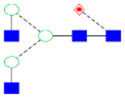 |
| 2.2 | | G0FB-N | F(6)A1B | [h3n4f1] | 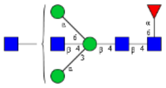 | 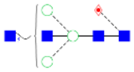 |
| 2.3 | | Man3F+2N | F(6)M3A2 | [h3n4f1] | 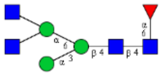 | 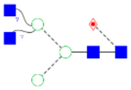 |
| 79 | | [h3n4f1a1] | | [h3n4f1a1] | | |
| 80 | | [h3n4f1S] | | [h3n4f1S] | | |
| 80.1 | | G0F-N+GalNAc(4-Sul) | FA1GalNAc(4-SO3)(4)1 | [h3n4f1S] | 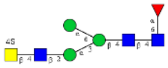 | 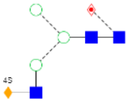 |
| 81 | | [h3n4f2] | | [h3n4f2] | | |
| 81.1 | | G0F2-N[3]+GalNAc | F(6)A1[3]F(3)GalNAc | [h3n4f2] | 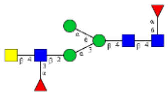 | 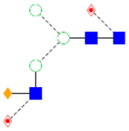 |
| 43 | | [h3n5] | | [h3n5] | | |

| Index[a] | Code[b] | Measurand[c] | Oxford[d] | Composition[e] | CFG[f] | Oxford[g] |
|---|---|---|---|---|---|---|
| 43.1 | 24 | G0B | A2B | [h3n5] | 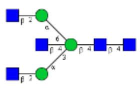 | 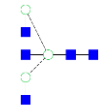 |
| 43.2 | | G0+N (tri) | A3 | [h3n5] | 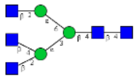 | 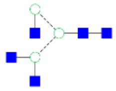 |
| 18 | | [h3n5f1] | | [h3n5f1] | | |
| 18.1 | 08 | G0FB | F(6)A2B | [h3n5f1] | 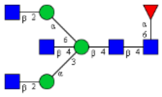 | 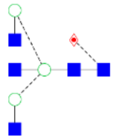 |
| 18.2 | | G0F+N (tri) | F(6)A3 | [h3n5f1] | 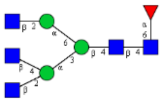 | 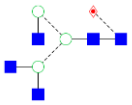 |
| 18.3 | | ManF+3N | F(6)A3 or F(6)A2B | [h3n5f1] | 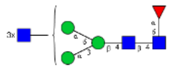 | 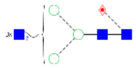 |
| 18.31 | | G0F+N | F(6)A2HexNAc1 | [h3n5f1] | 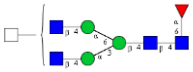 | 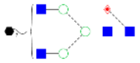 |
| 82 | | [h3n5f2] | | [h3n5f2] | | |
| 83 | | [h3n7f1] | | [h3n7f1] | | |
| 83.1 | | G0FB+2N (quad) | F(6)A4B | [h3n7f1] | 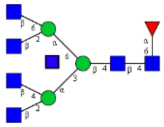 | 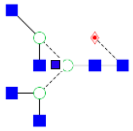 |
| 53 | | [h4n2] | | [h4n2] | | |
| 53.1 | | Man4 | M4 | [h4n2] | 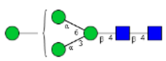 | 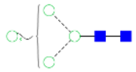 |
| 53.11 | | Man4D2 | M4D2 | [h4n2] | 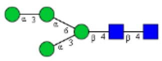 | 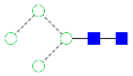 |
| 64 | | [h4n2f1] | | [h4n2f1] | | |
| 64.1 | | Man4F | F(6)M4 | [h4n2f1] | 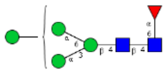 | 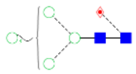 |
| 64.11 | | Man4D2F | F(6)M4D2 | [h4n2f1] | 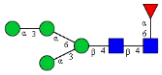 | 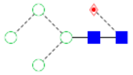 |
| 22 | | [h4n3] | | [h4n3] | | |
| 22.1 | 22 | G1-N | A1G(4)1 | [h4n3] | 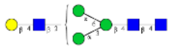 | 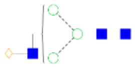 |

| Index[a] | Code[b] | Measurand[c] | Oxford[d] | Composition[e] | CFG[f] | Oxford[g] |
|---|---|---|---|---|---|---|
| 22.2[h] | | Man4+N | M4A1 | [h4n3] | 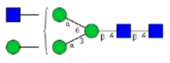 | 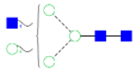 |
| 22.21[h] | | Man4+N[3] | M4[3]A1 | [h4n3] | 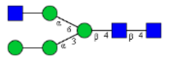 | 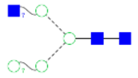 |
| 84 | | [h4n3a1] | | [h4n3a1] | | |
| 84.1 | | G1S-N (NeuAc) | A1G1Sa1 | [h4n3a1] | 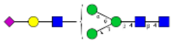 | 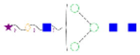 |
| 10 | | [h4n3f1] | | [h4n3f1] | | |
| 10.1 | 05 | G1F-N | F(6)A1G(4)1 | [h4n3f1] | 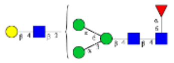 | 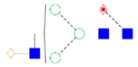 |
| 10.11 | 05a | G1F-N[6] | F(6)A1[6]G(4)1 | [h4n3f1] | 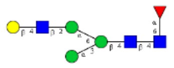 | 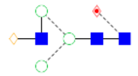 |
| 10.12 | 05b | G1F-N[3] | F(6)A1[3]G(4)1 | [h4n3f1] | 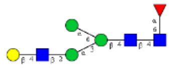 | 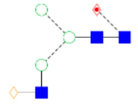 |
| 10.2[h] | | Man4F+N | F(6)M4A1 | [h4n3f1] | 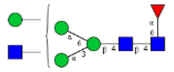 | 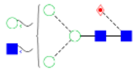 |
| 36 | | [h4n3f1a1] | | [h4n3f1a1] | | |
| 36.1 | 29 | G1FS-N (NeuAc) | F(6)A1G(4)1Sa1 | [h4n3f1a1] | 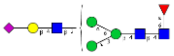 | 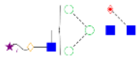 |
| 12 | | [h4n3f1g1] | | [h4n3f1g1] | | |
| 12.1 | 35 | G1FS-N (NeuGc) | F(6)A1G(4)1Sg1 | [h4n3f1g1] | 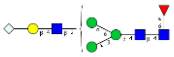 | 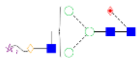 |
| 44 | | [h4n3f2] | | [h4n3f2] | | |
| 44.1 | | G1F2-N | FA1F1G1 | [h4n3f2] | 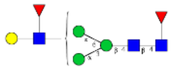 | 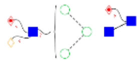 |
| 54 | | [h4n3g1] | | [h4n3g1] | | |
| 54.1 | | G1S-N (NeuGc) | A1G(4)1Sg1 | [h4n3g1] | 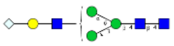 | 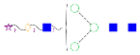 |
| 20 | | [h4n4] | | [h4n4] | | |
| 20.1 | 19 | G1 | A2G(4)1 | [h4n4] | 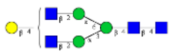 | 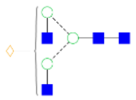 |
| 20.11 | 19a | G1[6] | A2[6]G(4)1 | [h4n4] | 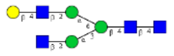 | 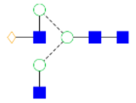 |
| 20.12 | 19b | G1[3] | A2[3]G(4)1 | [h4n4] | 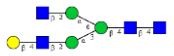 | 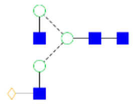 |

| Index[a] | Code[b] | Measurand[c] | Oxford[d] | Composition[e] | CFG[f] | Oxford[g] |
|---|---|---|---|---|---|---|
| 20.2 | | G1B-N | A1BG1 | [h4n4] | 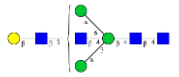 | 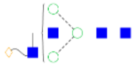 |
| 65 | | [h4n4a1] | | [h4n4a1] | | |
| 65.1 | | G1S (NeuAc) | A2G1Sa1 | [h4n4a1] | 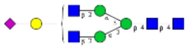 | 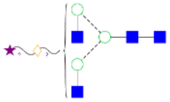 |
| 1 | | [h4n4f1] | | [h4n4f1] | | |
| 1.1 | 02 | G1F | F(6)A2G(4)1 | [h4n4f1] | 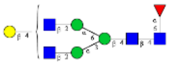 | 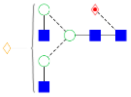 |
| 1.11 | 02a | G1F[6] | F(6)A2[6]G(4)1 | [h4n4f1] | 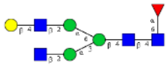 | 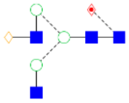 |
| 1.12 | 02b | G1F[3] | F(6)A2[3]G(4)1 | [h4n4f1] | 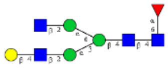 | 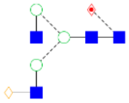 |
| 1.2 | | G1FB-N | F(6)A1BG(4)1 | [h4n4f1] | 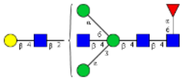 | 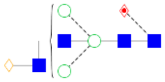 |
| 1.21 | | G1FB-N[3] | F(6)A1[3]BG(4)1 | [h4n4f1] | 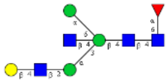 | 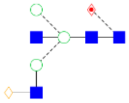 |
| 1.22 | | G1FB-N[6] | F(6)A1[6]BG(4)1 | [h4n4f1] | 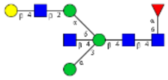 | 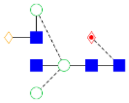 |
| 40 | | [h4n4f1a1] | | [h4n4f1a1] | | |
| 40.1 | 30 | G1FS (NeuAc) | F(6)A2G(4)1Sa1 | [h4n4f1a1] | 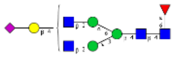 | 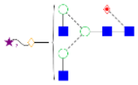 |
| 14 | | [h4n4f1g1] | | [h4n4f1g1] | | |
| 14.1 | 36 | G1FS (NeuGc) | F(6)A2G(4)1Sg1 | [h4n4f1g1] | 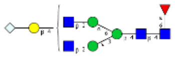 | 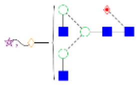 |
| 14.11 | | G1FS[6] (NeuGc) | F(6)A2[6]G(4)1Sg(6)1 | [h4n4f1g1] | 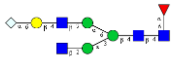 | 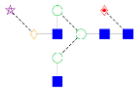 |
| 14.12 | | G1FS[3] (NeuGc) | F(6)A2[3]G(4)1Sg(6)1 | [h4n4f1g1] | 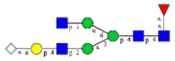 | 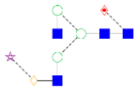 |
| 85 | | [h4n4f1g2] | | [h4n4f1g2] | | |

| Index[a] | Code[b] | Measurand[c] | Oxford[d] | Composition[e] | CFG[f] | Oxford[g] |
|---|---|---|---|---|---|---|
| 85.1 | | G1FS2 (NeuGc) | F(6)A2G1Sg2 | [h4n4f1g2] | 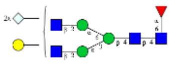 | 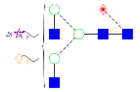 |
| 86 | | [h4n4f1S] | | [h4n4f1S] | | |
| 86.1 | | Man4F+N+GalNAc (4Sul) hybrid | F(6)M4A1GalNAc(4-SO3)(4)1 | [h4n4f1S] | 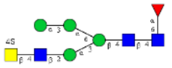 | 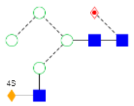 |
| 38 | | [h4n4f2] | | [h4n4f2] | | |
| 38.1 | 11 | G1F2 | F(6)A2F1G(4)1 | [h4n4f2] | 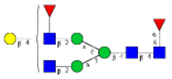 | 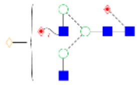 |
| 57 | | [h4n5] | | [h4n5] | | |
| 57.1 | 25 | G1B | A2BG(4)1 | [h4n5] | 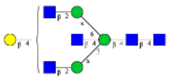 | 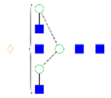 |
| 57.11 | 25a | G1B[6] | A2[6]BG(4)1 | [h4n5] | 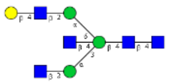 | 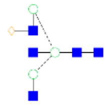 |
| 57.12 | 25b | G1B[3] | A2[3]BG(4)1 | [h4n5] | 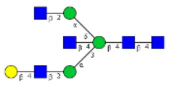 | 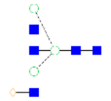 |
| 87 | | [h4n5a1] | | [h4n5a1] | | |
| 87.1 | | G1S+N (NeuAc) (tri) | A3G1Sa1 | [h4n5a1] | 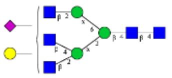 | 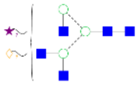 |
| 8 | | [h4n5f1] | | [h4n5f1] | | |
| 8.1 | 09 | G1FB | F(6)A2BG(4)1 | [h4n5f1] | 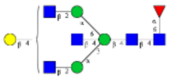 | 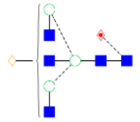 |
| 8.2 | | G1F+N (tri) | F(6)A3G(4)1 | [h4n5f1] | 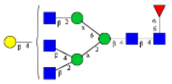 | 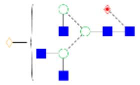 |
| 8.3 | | G0F+Hex+HexNAc | F(6)A2Hex1HexNAc1 | [h4n5f1] | 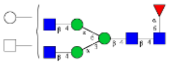 | 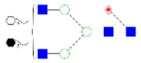 |
| 8.4 | | G1F+N | FA2HexNAc1G1 | [h4n5f1] | 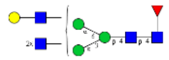 | 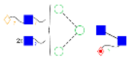 |
| 70 | | [h4n5f1a1] | | [h4n5f1a1] | | |
| 70.1 | 31 | G1FBS | FA2BG(4)1Sa1 | [h4n5f1a1] | 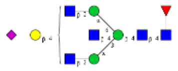 | 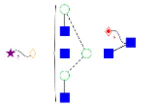 |
| 45 | | [h4n5f1g1] | | [h4n5f1g1] | | |

| Index[a] | Code[b] | Measurand[c] | Oxford[d] | Composition[e] | CFG[f] | Oxford[g] |
|---|---|---|---|---|---|---|
| 45.1 | 37 | G1FBS (NeuGc) | FA2BG(4)1Sg1 | [h4n5f1g1] | 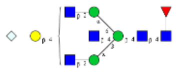 | 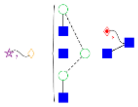 |
| 5 | | [h5n2] | | [h5n2] | | |
| 5.1 | 45 | Man5 | M5 | [h5n2] | 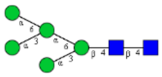 | 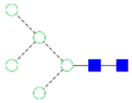 |
| 88 | | [h5n2f1] | | [h5n2f1] | | |
| 88.1 | | Man5F | F(6)M5 | [h5n2f1] | 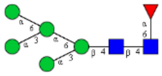 | 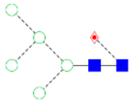 |
| 29 | | [h5n3] | | [h5n3] | | |
| 29.1 | 23 | G1-N+1aGal | A1G(4)1Ga(3)1 | [h5n3] | 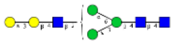 | 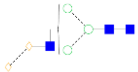 |
| 46 | | [h5n3a1] | | [h5n3a1] | | |
| 46.1 | | Man4G1S hybrid | M4A1G(4)1Sa(6)1 | [h5n3a1] | 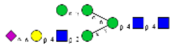 | 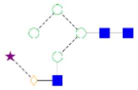 |
| 46.2 | | Fragment G2S-CoreN (NeuAc) | A2G2Sa1 without core GlcNAc | [h5n3a1] | 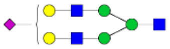 | 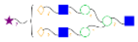 |
| 9 | | [h5n3f1] | | [h5n3f1] | | |
| 9.1 | 06 | G1F-N+1aGal | F(6)A1G(4)1Ga(3)1 | [h5n3f1] | 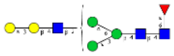 | 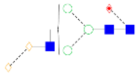 |
| 9.2 | | Man5G0F hybrid | F(6)M5A1[3] | [h5n3f1] | 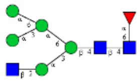 | 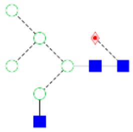 |
| 9.3[h] | | Man4G1F | F(6)M4A1G(4)1 | [h5n3f1] | 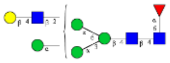 | 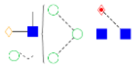 |
| 9.31 | | Man4G1[3]F hybrid | F(6)M4A1[3]G(4)1 | [h5n3f1] | 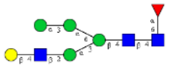 | 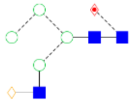 |
| 71 | | [h5n3f1a1] | | [h5n3f1a1] | | |
| 71.1 | | Man4G1[3]FS (NeuAc) hybrid | F(6)M4A1G(4)1Sa1 | [h5n3f1a1] | 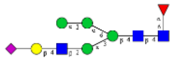 | 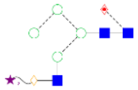 |
| 71.2 | | G1FS-N+1aGal (NeuAc) | FA1G1Ga1Sa1 | [h5n3f1a1] | 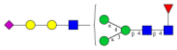 | 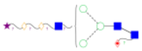 |
| 26 | | [h5n3f1g1] | | [h5n3f1g1] | | |

| Index[a] | Code[b] | Measurand[c] | Oxford[d] | Composition[e] | CFG[f] | Oxford[g] |
|---|---|---|---|---|---|---|
| 26.1 | 47 | Man5G0FS (NeuGc) hybrid | F(6)M5A1Sg1 | [h5n3f1g1] | 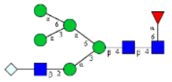 | 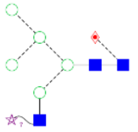 |
| 26.2[h] | | Man4G1FS (NeuGc) | F(6)M4A1G(4)1Sg(6)1 | [h5n3f1g1] | 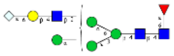 | 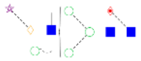 |
| 58 | | [h5n3f2] | | [h5n3f2] | | |
| 58.1 | 07 | G1F2-N+1aGal | F(6)A1F1G1Ga(3)1 | [h5n3f2] | 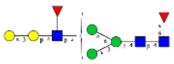 | 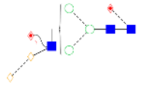 |
| 66 | | [h5n3g1] | | [h5n3g1] | | |
| 66.1[h] | | Man4G1S1 (NeuGc) | M4A1G1Sg1 | [h5n3g1] | 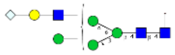 | 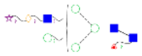 |
| 21 | | [h5n4] | | [h5n4] | | |
| 21.1 | 20 | G2 | A2G(4)2 | [h5n4] | 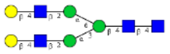 | 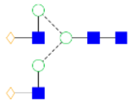 |
| 21.2 | | G1+1aGal | A2G(4)1Ga(3)1 | [h5n4] | 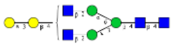 | 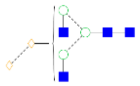 |
| 27 | | [h5n4a1] | | [h5n4a1] | | |
| 27.1 | 41 | G2S (NeuAc) | A2G(4)2Sa1 | [h5n4a1] | 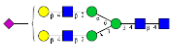 | 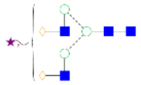 |
| 59 | | [h5n4a2] | | [h5n4a2] | | |
| 59.1 | 42 | G2S2 (NeuAc) | A2G(4)2Sa2 | [h5n4a2] | 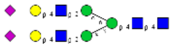 | 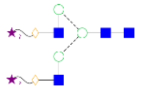 |
| 3 | | [h5n4f1] | | [h5n4f1] | | |
| 3.1 | 03a | G2F | F(6)A2G(4)2 | [h5n4f1] | 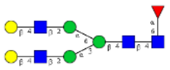 | 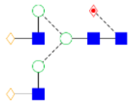 |
| 3.2 | 03b | G1F+1aGal | F(6)A2G(4)1Ga(3)1 | [h5n4f1] | 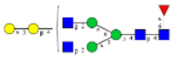 | 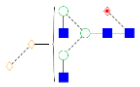 |
| 3.21 | | G1F[3]+1aGal | F(6)A2[3]G(4)1Ga(3)1 | [h5n4f1] | 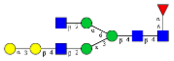 | 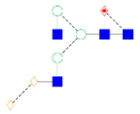 |
| 3.22 | | G1F[6]+1aGal | F(6)A2[6]G(4)1Ga(3)1 | [h5n4f1] | 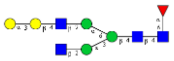 | 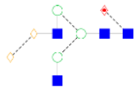 |

| Index[a] | Code[b] | Measurand[c] | Oxford[d] | Composition[e] | CFG[f] | Oxford[g] |
|---|---|---|---|---|---|---|
| 3.3 | | G1FB-N+1aGal | F(6)A1BG(4)1Ga(3)1 | [h5n4f1] | 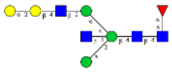 | 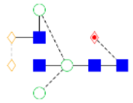 |
| 35 | | [h5n4f1a1] | | [h5n4f1a1] | | |
| 35.1 | 32 | G2FS (NeuAc) | F(6)A2G(4)2Sa1 | [h5n4f1a1] | 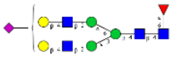 | 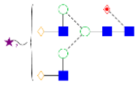 |
| 41 | | [h5n4f1a2] | | [h5n4f1a2] | | |
| 41.1 | 33 | G2FS2 (NeuAc) | F(6)A2G(4)2Sa2 | [h5n4f1a2] | 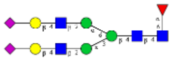 | 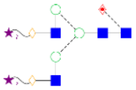 |
| 16 | | [h5n4f1g1] | | [h5n4f1g1] | | |
| 16.1 | 38 | G2FS (NeuGc) | F(6)A2G(4)2Sg1 | [h5n4f1g1] | 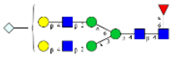 | 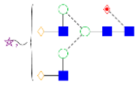 |
| 55 | | [h5n4f1g2] | | [h5n4f1g2] | | |
| 55.1 | 39 | G2FS2 (NeuGc) | F(6)A2G(4)2Sg2 | [h5n4f1g2] | 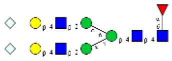 | 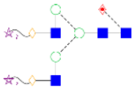 |
| 47 | | [h5n4f2] | | [h5n4f2] | | |
| 47.1 | 12 | G2F2 | F(6)A2F1G(4)2 | [h5n4f2] | 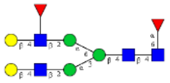 | 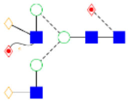 |
| 47.2 | | Man4G1F2B hybrid | F(6)M4A1BF(3)1G(4)1 | [h5n4f2] | 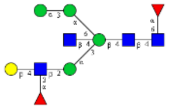 | 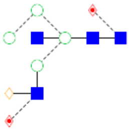 |
| 60 | | [h5n4g1] | | [h5n4g1] | | |
| 60.1 | 43 | G2S (NeuGc) | A2G(4)2Sg1 | [h5n4g1] | 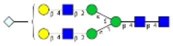 | 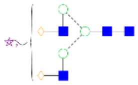 |
| 48 | | [h5n4g2] | | [h5n4g2] | | |
| 48.1 | 44 | G2S2 (NeuGc) | A2G(4)2Sg2 | [h5n4g2] | 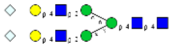 | 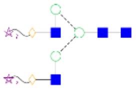 |
| 67 | | [h5n5] | | [h5n5] | | |
| 67.1 | 26 | G2B | A2BG(4)2 | [h5n5] | 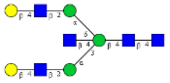 | 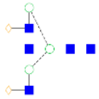 |
| 67.2 | | G1+N+1aGal (tri) | A3G(4)1Ga(3)1 | [h5n5] | 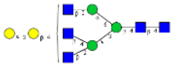 | 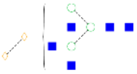 |
| 17 | | [h5n5f1] | | [h5n5f1] | | |

| Index[a] | Code[b] | Measurand[c] | Oxford[d] | Composition[e] | CFG[f] | Oxford[g] |
|---|---|---|---|---|---|---|
| 17.1 | 10 | G2FB | F(6)A2BG(4)2 | [h5n5f1] | 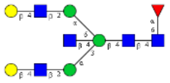 | 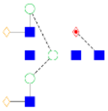 |
| 17.2 | | G1FB+1aGal | F(6)A2BG(4)Ga(2)1 | [h5n5f1] | 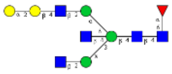 | 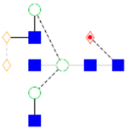 |
| 17.3 | | G2F+N (tri) | FA3G2 | [h5n5f1] | 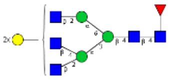 | 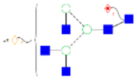 |
| 17.31 | | G(4)1F+N+1aGal | F(6)A3G(4)1Ga(3)1 | [h5n5f1] | 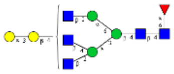 | 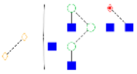 |
| 17.32 | | G(4)2F+N (tri) | F(6)A3G(4)2 | [h5n5f1] | 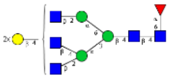 | 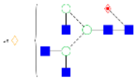 |
| 17.4 | | G0F+2Hex+HexNAc | F(6)A2Hex2HexNAc1 | [h5n5f1] | 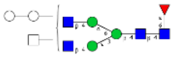 | 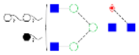 |
| 89 | | [h5n5f1a1] | | [h5n5f1a1] | | |
| 89.1 | | G2FS+N (NeuAc) (tri) | FA3G2S1 | [h5n5f1a1] | 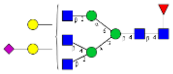 | 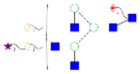 |
| 90 | | [h5n5f1a2] | | [h5n5f1a2] | | |
| 90.1 | | G2FS2+N (NeuAc) (tri) | FA3G2Sa2 | [h5n5f1a2] | 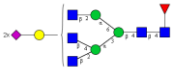 | 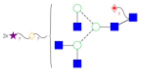 |
| 91 | | [h5n5f1g1] | | [h5n5f1g1] | | |
| 61 | | [h5n5f2] | | [h5n5f2] | | |
| 61.1 | | G2F2B | FA2BG2F | [h5n5f2] | 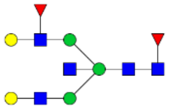 | 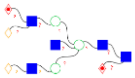 |
| 61.2 | | G2F2+N (tri) | FA3F1G2 | [h5n5f2] | 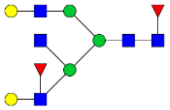 | 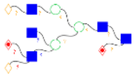 |
| 30 | | [h6n2] | | [h6n2] | | |
| 30.1 | 46 | Man6 | M6 | [h6n2] | 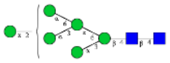 | 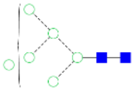 |
| 92 | | [h6n2f1] | | [h6n2f1] | | |
| 92.1 | | Man6F | F(6)M6 D3 | [h6n2f1] | 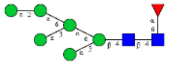 | 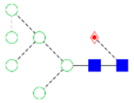 |
| 110 | | [h6n2g1] | | [h6n2g1] | | |

| Index[a] | Code[b] | Measurand[c] | Oxford[d] | Composition[e] | CFG[f] | Oxford[g] |
|---|---|---|---|---|---|---|
| 110.1 | | Man5+Gal+S (NeuGc) hybrid | M5G1Sg1 | [h6n2g1] | 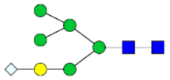 | 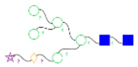 |
| 28 | | [h6n3] | | [h6n3] | | |
| 28.1 | 48 | Man5G1 hybrid | M5A1G(4)1 | [h6n3] | 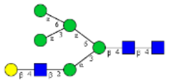 | 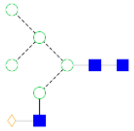 |
| 72 | | [h6n3a1] | | [h6n3a1] | | |
| 24 | | [h6n3f1] | | [h6n3f1] | | |
| 24.1 | 49 | Man5G1F hybrid | F(6)M5A1G(4)1 | [h6n3f1] | 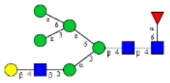 | 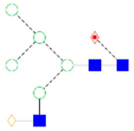 |
| 24.2 | | Man4G1F+1aGal hybrid | F(6)M4A1G(4)1Ga(3)1 | [h6n3f1] | 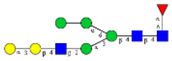 | 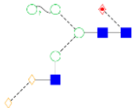 |
| 111 | | [h6n3f1a1] | | [h6n3f1a1] | | |
| 111.1 | | Man5G1FS (NeuAc) hybrid | F(6)M5A1G(4)1Sa(3)1 | [h6n3f1a1] | 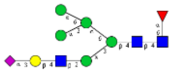 | 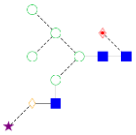 |
| 32 | | [h6n3f1g1] | | [h6n3f1g1] | | |
| 32.1 | 50 | Man5G1FS (NeuAc) hybrid | F(6)M5A1G(4)1Sg1 | [h6n3f1g1] | 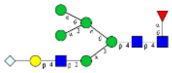 | 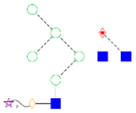 |
| 93 | | [h6n3f2] | | [h6n3f2] | | |
| 112 | | [h6n3f2a1] | | [h6n3f2a1] | | |
| 112.1 | | Man5G1F2S (NeuAc) hybrid | F(6)M5A1F(3)1G(4)1S a1 | [h6n3f2a1] | 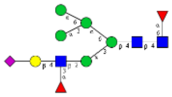 | 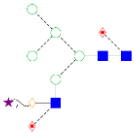 |
| 49 | | [h6n3g1] | | [h6n3g1] | | |
| 49.1 | | Man5G1S (NeuGc) hybrid | M5A1G1Sg1 | [h6n3g1] | 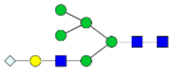 | 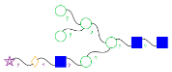 |
| 50 | | [h6n4] | | [h6n4] | | |
| 50.1 | 27 | G2+1aGal | A2G(4)2Ga(3)1 | [h6n4] | 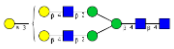 | 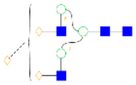 |

| Index[a] | Code[b] | Measurand[c] | Oxford[d] | Composition[e] | CFG[f] | Oxford[g] |
|---|---|---|---|---|---|---|
| 50.2 | | Fragment Man5+2N+Hex-CoreN hybrid | M5A2Hex1 without core GlcNAc | [h6n4] | 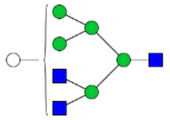 | 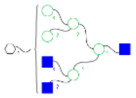 |
| 73 | | [h6n4a1] | | [h6n4a1] | | |
| 73.1 | | G2S+1aGal (NeuAc) | A2G2Ga1Sa1 | [h6n4a1] | 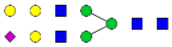 | 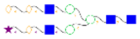 |
| 7 | | [h6n4f1] | | [h6n4f1] | | |
| 7.1 | 13 | G2F+1aGal | F(6)A2G(4)2Ga(3)1 | [h6n4f1] | 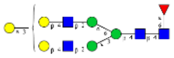 | 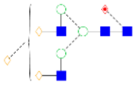 |
| 7.11 | | G2F+1aGal[6] | F(6)A2[6]G(4)2Ga(3)1 | [h6n4f1] | 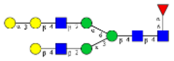 | 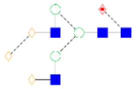 |
| 7.12 | | G2F+1aGal[3] | F(6)A2[3]G(4)2Ga(3)1 | [h6n4f1] | 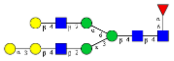 | 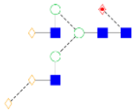 |
| 51 | | [h6n4f1a1] | | [h6n4f1a1] | | |
| 51.1 | 34 | G2FS+1aGal (NeuAc) | F(6)A2G(4)2Ga(3)1Sa1 | [h6n4f1a1] | 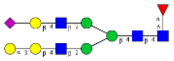 | 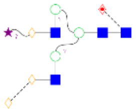 |
| 51.2 | | Man5G1FBS (NeuAc) hybrid | F(6)M5A1BG(4)1Sa1 | [h6n4f1a1] | 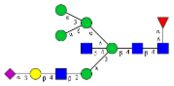 | 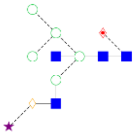 |
| 15 | | [h6n4f1g1] | | [h6n4f1g1] | | |
| 15.1 | 40 | G2FS+1aGal (NeuGc) | F(6)A2G(4)2Ga(3)1Sg1 | [h6n4f1g1] | 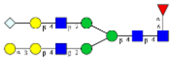 | 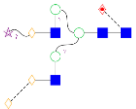 |
| 31 | | [h6n4f2] | | [h6n4f2] | | |
| 31.1 | 14 | G2F2+1aGal | F(6)A2F1G(4)2Ga(3)1 | [h6n4f2] | 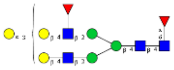 | 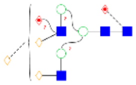 |
| 31.2 | | Man5G1F2B hybrid | F(6)M5A1BF(3)1G(4)1 | [h6n4f2] | 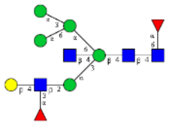 | 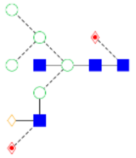 |
| 94 | | [h6n4g1] | | [h6n4g1] | | |
| 74 | | [h6n5] | | [h6n5] | | |
| 74.1 | | G3 | A3G3 | [h6n5] | 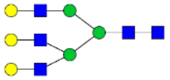 | 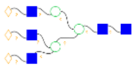 |

| Index[a] | Code[b] | Measurand[c] | Oxford[d] | Composition[e] | CFG[f] | Oxford[g] |
|---|---|---|---|---|---|---|
| 74.11 | | G(4)3 | A3G(4)3 | [h6n5] | 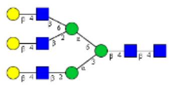 | 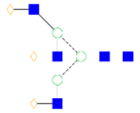 |
| 75 | | [h6n5a1] | | [h6n5a1] | | |
| 25 | | [h6n5f1] | | [h6n5f1] | | |
| 25.1 | 15 | G2FB+1aGal | F(6)A2BG(4)2Ga(3)1 | [h6n5f1] | 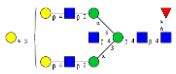 | 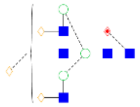 |
| 25.2 | | G3F | FA3G3 | [h6n5f1] | 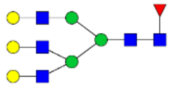 | 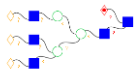 |
| 25.21 | | G(4)3F | F(6)A3G(4)3 | [h6n5f1] | 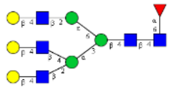 | 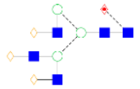 |
| 68 | | [h6n5f1g1] | | [h6n5f1g1] | | |
| 68.1 | | G2FBS+1aGal (NeuGc) | FA2BG2Ga1Sg1 | [h6n5f1g1] | 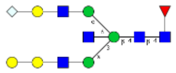 | 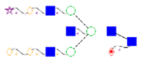 |
| 68.2 | | G3FS (NeuGc) | F(6)A3G(4)3Sg(6)1 | [h6n5f1g1] | 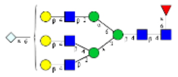 | 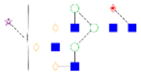 |
| 95 | | [h6n5f1g2] | | [h6n5f1g2] | | |
| 95.1 | | G3FS2(NeuGc) | FA3G3Sg2 | [h6n5f1g2] | 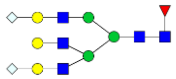 | 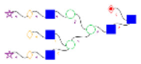 |
| 96 | | [h6n7f4a3] | | [h6n7f4a3] | | |
| 97 | | [h6n7f5a2] | | [h6n7f5a2] | | |
| 52 | | [h7n2] | | [h7n2] | | |
| 52.1 | | Man7 | M7 | [h7n2] | 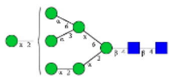 | 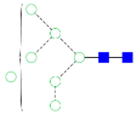 |
| 39 | | [h7n3] | | [h7n3] | | |
| 39.1 | 51 | Man5G1+1aGal hybrid | M5A1G(4)1Ga(3)1 | [h7n3] | 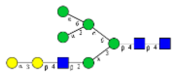 | 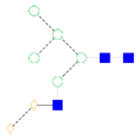 |
| 33 | | [h7n3f1] | | [h7n3f1] | | |
| 33.1 | 52 | Man5G1F+1aGal hybrid | F(6)M5A1G(4)1Ga(3)1 | [h7n3f1] | 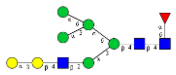 | 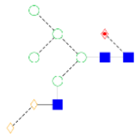 |
| 98 | | [h7n3f2] | | [h7n3f2] | | |
| 99 | | [h7n4] | | [h7n4] | | |

| Index[a] | Code[b] | Measurand[c] | Oxford[d] | Composition[e] | CFG[f] | Oxford[g] |
|---|---|---|---|---|---|---|
| 99.1 | 28 | G2+2aGal | A2G2Ga2 | [h7n4] | 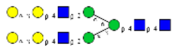 | 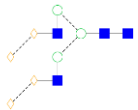 |
| 62 | | [h7n4a1] | | [h7n4a1] | | |
| 62.1 | | G2S+2aGal (NeuAc) | A2G2Ga2Sa1 | [h7n4a1] | 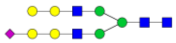 | 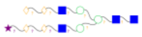 |
| 62.2 | | Man5G2S (NeuAc) hybrid | M5A2G2Sa1 | [h7n4a1] | 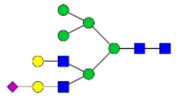 | 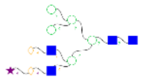 |
| 62.3 | | G2S(6)+1aGal+Gal (NeuAc) | A2G(4)2Ga(3)1Sa1 | [h7n4a1] | 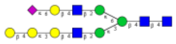 | 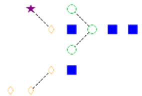 |
| 6 | | [h7n4f1] | | [h7n4f1] | | |
| 6.1 | 16 | G2F+2aGal | F(6)A2G(4)2Ga(3)2 | [h7n4f1] | 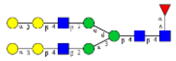 | 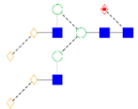 |
| 6.2 | | Man5G2F hybrid | FM5A2G2 | [h7n4f1] | 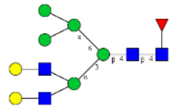 | 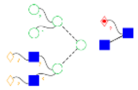 |
| 23 | | [h7n5f1] | | [h7n5f1] | | |
| 23.1 | 17 | G2FB+2aGal | F(6)A2BG(4)2Ga(3)2 | [h7n5f1] | 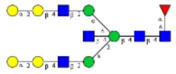 | 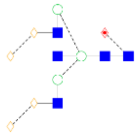 |
| 23.2 | | G3F+1aGal | FA3G3Ga(3)1 | [h7n5f1] | 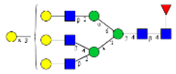 | 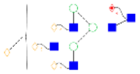 |
| 23.21 | | G(4)3F+1aGal | F(6)A3G(4)3Ga(3)1<br>F(6)A3G(4)3Ga1 | [h7n5f1] | 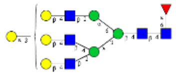 | 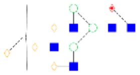 |
| 23.3 | | G2F+N+2aGal (tri) | F(6)A3G(4)2Ga(3)2 | [h7n5f1] | 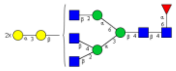 | 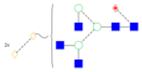 |
| 23.4[h] | | Man5F+3N+2Gal | F(6)M5GlcNAc3G2 | [h7n5f1] | 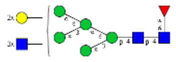 | 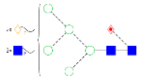 |
| 113 | | [h7n5f1g1] | | [h7n5f1g1] | | |
| 113.1 | | G2FBGS+1aGal (NeuGc) | FA2BG3Ga1Sg1 | [h7n5f1g1] | 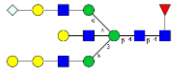 | 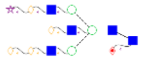 |
| 100 | | [h7n5f1g2] | | [h7n5f1g2] | | |
| 100.1 | | G2FBGS2+1aGal (NeuGc) | FA2BG3Ga1Sg2 | [h7n5f1g2] | 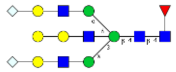 | 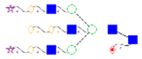 |
| 101 | | [h7n5f2] | | [h7n5f2] | | |

| Index[a] Code[b] | Measurand[c] | Oxford[d] | Composition[e] | CFG[f] | Oxford[g] |
|---|---|---|---|---|---|
| 114 | [h7n6f1a1g3] | | [h7n6f1a1g3] | | |
| 114.1 | G4FS4 (3NeuGc) (1NeuAc) | FA4G4Sa1Sg3 | [h7n6f1a1g3] | 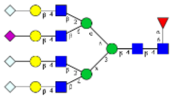 | 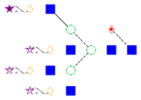 |
| 102 | [h7n6f2a1] | | [h7n6f2a1] | | |
| 102.1 | G4F2S (NeuAc) | FA4FG4Sa1 | [h7n6f2a1] | 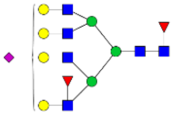 | 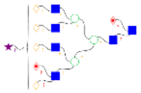 |
| 103 | [h7n8f3a4] | | [h7n8f3a4] | | |
| 115 | [h7n8f5a3] | | [h7n8f5a3] | | |
| 116 | [h7n8f6a3] | | [h7n8f6a3] | | |
| 104 | [h7n9f1a4] | | [h7n9f1a4] | | |
| 105 | [h8n10f5a3] | | [h8n10f5a3] | | |
| 63 | [h8n2] | | [h8n2] | | |
| 63.1 | Man8 | M8 | [h8n2] | 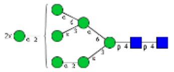 | 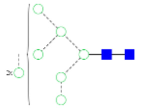 |
| 56 | [h8n5f1] | | [h8n5f1] | | |
| 56.1 | G3F+2aGal | F(6)A3G(4)3Ga2 | [h8n5f1] | 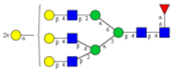 | 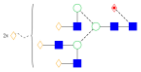 |
| 56.2 | G2FBG+2aGal | FA2BG3Ga2 | [h8n5f1] | 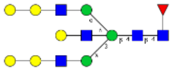 | 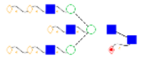 |
| 69 | [h8n5f1g1] | | [h8n5f1g1] | | |
| 69.1 | G3FS+2aGal (NeuGc) | F(6)A3G(4)3Ga(3)2Sg1 | [h8n5f1g1] | 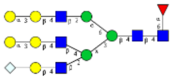 | 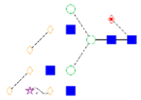 |
| 69.2 | G2FBGS+3aGal (NeuGc) | FA2BG3Ga3Sg1 | [h8n5f1g1] | 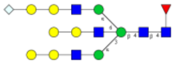 | 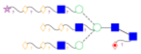 |
| 76 | [h8n5f2] | | [h8n5f2] | | |
| 106 | [h8n8f3a4] | | [h8n8f3a4] | | |
| 77 | [h9n2] | | [h9n2] | | |
| 77.1 | Man9 | M9 | [h9n2] | 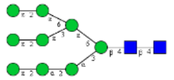 | 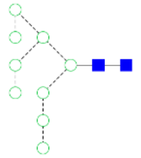 |
| 34 | [h9n5f1] | | [h9n5f1] | | |
| 34.1 | G3F+3aGal | F(6)A3G(4)3Ga(3)3 | [h9n5f1] | 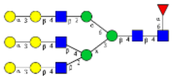 | 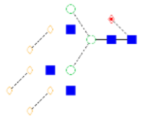 |

| Index[a] | Code[b] | Measurand[c] | Oxford[d] | Composition[e] | CFG[f] | Oxford[g] |
|---|---|---|---|---|---|---|
| 34.2 | | G2FBG+3aGal | FA2BG3Ga3 | [h9n5f1] | 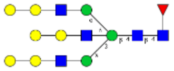 | 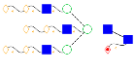 |
| 107 | | [n1f1] | | [n1f1] | | |
| 108 | | [n2f1] | | [n2f1] | | |
| 108.1 | | Fragment FN2 | F(6)GlcNAc2 | [n2f1] | 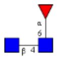 | 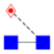 |
| | | G0F-N/G0F | FA1/FA2 | [h3n3f1]/ [h3n4f1] | | |
| | | G0/G1F | A2/FA2G1 | [h3n4]/ [h3n4f1] | | |
| | | G0F/G1F | FA2/FA2G1 | [h3n4f1]/ [h4n4f1] | | |
| | | G1F/G2F | FA2G1/FA2G2 | [h4n4f1]/ [h5n4f1] | | |
| | | G2F/ G2F+1aGal | FA2G2/FA2G2Ga1 | [h5n4f1]/ [h6n4f1] | | |
| | | | | [h5n5f1g1]/ [h6n5a1] | | |
| | | | | [h6n5a3]/ [h5n5f1a2g1]/ [h4n5f2a1g2]/ [h3n5f3g3] | | |
| | | | | [h6n5f1a3]/ [h5n5f2a2g1]/ [h4n5f3a1g2]/ [h3n5f4g3] | | |
| | | [Unknown] | *Sum of results reported for "unidentified" glycan-like signals* | | | |

a Index: Each different glycan composition is represented by an integer. Individual glycans are represented by a digit following a decimal point – isomers of these are represented by an additional digit. Indices in **bold** are glycans with complete structural assignments.

b Code: These correspond to numbers in the data reporting template. Entries with code *Other* are glycans reported by participants but not in the data reporting template.

c Measurands: text in square brackets correspond to monosaccharide compositions (see Composition). Common names are listed when available.

d Oxford: Oxford naming convention: All N-glycans have two core GlcNAcs; F at the start of the abbreviation indicates a core fucose, (6) after the F indicates that the fucose is α1-6 linked to the inner GlcNAc; Mx, number (x) of mannose on core GlcNAcs; Ax, number of antenna (GlcNAc) on trimannosyl core; A2, biantennary with both GlcNAcs as β1-2 linked; A3, triantennary with a GlcNAc linked β1-2 to both mannose and the third GlcNAc linked β1-4 to the α1-3 linked mannose; A3',

triantennary with a GlcNAc linked β1-2 to both mannose and the third GlcNAc linked β1-6 to the α1-6 linked mannose; A4, GlcNAcs linked as A3 with additional GlcNAc β1-6 linked to α1-6 mannose; B, bisecting GlcNAc linked β1-4 to β1-3 mannose; Gx, number (x) of linked galactose on antenna, (4) or (3) after the G indicates that the Gal is β1-4 or β1-3 linked; [3]G1 and [6]G1 indicates that the galactose is on the antenna of the α1-3 or α1-6 mannose; Gax, number (x) of linked alpha galactose on antenna; Sx, number (x) of sialic acids linked to galactose; Sa is used when the sialic acid is known to be *N*-acetylneuraminic acid, or Sg is used when it is known to be *N*-glycolyl neuraminic acid; the numbers 3 or 6 in parentheses after S indicate whether the sialic acid is in an α2-3 or α2-6 linkage. (Courtesy of Louise Royle, Ludger)

e [Composition] denotes monosaccharide composition. Small letters are used to avoid confusion with elements (hydrogen, nitrogen, fluorine, etc.): h=hexose, n=N-acetylhexosamine, f=deoxyhexose (e.g. fucose), a=NeuAc, g=NeuGc. Number after the letter denotes the number of residues. For example: [h6n4f1a1] = 6 hexoses, 4 N-acetylhexosamine, 1 fucose, 1 NeuAc. For sulfonated glycans, S=sulfur.

f CFG: Structure using the Consortium for Functional Glycomics (CFG) Notation: Symbol representations of glycans: galactose= glucose=, mannose=, N-Acetylgalactosamine= N-Acetylglucosamine= fucose=, xylose=, N-Acetylneuraminic acid=, N-Glycolylneuraminic acid= (Varki A et al. *Proteomics* 9, 5398–5399, **2009**; Ceroni A et al. *J Proteome Res*, 7(4), 1650–1659, **2008**)

g Oxford: Structure using the Oxford Glycobiology Institute (UOXF) Notation: galactose= , glucose=, mannose=, N-Acetylgalactosamine=, N-Acetylglucosamine=, fucose=, xylose=, N-Acetylneuraminic acid=, N-Glycolylneuraminic Acid= (Ceroni A et al. *J Proteome Res*, 7(4), 1650–1659, **2008**)

h A hybrid glycan if GlcNAc is on the 3-linked mannose (bottom) arm of the trimannosyl core

**Supplementary Table 3.** Summary of Reported and Derived Values

| Measurand | | | Sample A, % | | | | Sample B, % | | | | A/B Ratio | | | |
|---|---|---|---|---|---|---|---|---|---|---|---|---|---|---|
| Index | Composition | Common Name | # | 25% | Median | 75% | # | 25% | Median | 75% | # | 25% | Median | 75% |
| 1 | [h4n4f1] | | 103 | 27.72 | 31.61 | 33.31 | 103 | 36.36 | 38.37 | 39.84 | 103 | 0.77 | 0.83 | 0.85 |
| 1.1 | [h4n4f1] Gly | G1F | 103 | 27.72 | 31.61 | 33.31 | 103 | 36.36 | 38.37 | 39.84 | 103 | 0.77 | 0.83 | 0.85 |
| 1.11 | [h4n4f1] GlyIso | G1F[6] | 58 | 19.04 | 21.21 | 22.06 | 58 | 27.60 | 28.12 | 29.12 | 58 | 0.71 | 0.76 | 0.77 |
| 1.12 | [h4n4f1] GlyIso | G1F[3] | 59 | 9.38 | 10.38 | 10.72 | 59 | 9.82 | 10.18 | 10.75 | 59 | 0.93 | 1.02 | 1.03 |
| 1.2 | [h4n4f1] Gly | G1FB-N | 2 | | 1.06 | | 2 | | 1.21 | | 2 | | 0.92 | |
| 1.21 | [h4n4f1] GlyIso | G1FB-N[3] | 2 | | 0.64 | | 2 | | 0.74 | | 2 | | 1.28 | |
| 1.22 | [h4n4f1] GlyIso | G1FB-N[6] | 2 | | 0.43 | | 2 | | 0.47 | | 2 | | 0.94 | |
| 2 | [h3n4f1] | | 103 | 47.06 | 51.47 | 55.04 | 102 | 35.73 | 39.10 | 40.76 | 102 | 1.28 | 1.31 | 1.38 |
| 2.1 | [h3n4f1] Gly | G0F | 103 | 47.06 | 51.47 | 55.04 | 102 | 35.73 | 39.09 | 40.71 | 102 | 1.28 | 1.31 | 1.38 |
| 2.2 | [h3n4f1] Gly | G0FB-N | 1 | | 0.77 | | 1 | | 0.80 | | 1 | | 0.96 | |
| 2.3 | [h3n4f1] Gly | Man3F+2N | 2 | | 2.26 | | 2 | | 1.71 | | 2 | | 1.32 | |
| 3 | [h5n4f1] | | 99 | 3.19 | 3.97 | 4.80 | 102 | 7.47 | 8.52 | 9.48 | 99 | 0.42 | 0.47 | 0.55 |
| 3.1 | [h5n4f1] Gly | G2F | 96 | 1.27 | 3.08 | 4.67 | 101 | 6.53 | 7.51 | 8.93 | 96 | 0.23 | 0.46 | 0.71 |
| 3.2 | [h5n4f1] Gly | G1F+1aGal | 35 | 1.64 | 2.53 | 3.06 | 32 | 1.27 | 1.60 | 2.20 | 32 | 0.83 | 1.39 | 1.68 |
| 3.21 | [h5n4f1] GlyIso | G1F[3]+1aGal | 2 | | 0.25 | | 1 | | 0.17 | | 1 | | 1.47 | |
| 3.22 | [h5n4f1] GlyIso | G1F[6]+1aGal | 2 | | 2.88 | | 2 | | 1.61 | | 2 | | 1.79 | |
| 3.3 | [h5n4f1] Gly | G1FB-N+1aGal | 2 | | 1.18 | | 2 | | 4.01 | | 2 | | 0.58 | |
| 4 | [h3n3f1] | | 88 | 3.03 | 3.65 | 4.26 | 89 | 1.87 | 2.13 | 2.93 | 87 | 1.40 | 1.65 | 1.89 |
| 4.1 | [h3n3f1] Gly | G0F-N | 88 | 3.03 | 3.65 | 4.26 | 89 | 1.87 | 2.13 | 2.93 | 87 | 1.40 | 1.65 | 1.89 |
| 4.11 | [h3n3f1] GlyIso | G0F-N[6] | 10 | 0.88 | 1.73 | 3.31 | 9 | 0.94 | 1.61 | 1.84 | 9 | 1.31 | 1.39 | 1.82 |
| 4.12 | [h3n3f1] GlyIso | G0F-N[3] | 11 | 0.88 | 3.47 | 3.90 | 11 | 0.42 | 1.92 | 2.12 | 11 | 1.26 | 1.61 | 1.87 |
| 5 | [h5n2] | | 77 | 0.56 | 0.78 | 1.12 | 79 | 0.53 | 0.73 | 1.01 | 76 | 0.94 | 1.02 | 1.13 |
| 5.1 | [h5n2] Gly | Man5 | 77 | 0.56 | 0.78 | 1.12 | 79 | 0.53 | 0.73 | 1.01 | 76 | 0.94 | 1.02 | 1.13 |
| 6 | [h7n4f1] | | 73 | 0.70 | 0.90 | 1.09 | 71 | 0.68 | 0.90 | 1.22 | 71 | 0.90 | 0.98 | 1.03 |
| 6.1 | [h7n4f1] Gly | G2F+2aGal | 71 | 0.69 | 0.87 | 1.05 | 70 | 0.67 | 0.89 | 1.06 | 70 | 0.89 | 0.98 | 1.03 |
| 6.2 | [h7n4f1] Gly | Man5G2F hybrid | 1 | | 1.82 | | 1 | | 1.60 | | 1 | | 1.14 | |
| 7 | [h6n4f1] | | 70 | 0.44 | 0.63 | 0.92 | 73 | 1.48 | 1.80 | 2.09 | 69 | 0.29 | 0.35 | 0.45 |

| Measurand | | | Sample A, % | | | | Sample B, % | | | | A/B Ratio | | | |
|---|---|---|---|---|---|---|---|---|---|---|---|---|---|---|
| Index | Composition | Common Name | # | 25% | Median | 75% | # | 25% | Median | 75% | # | 25% | Median | 75% |
| 7.1 | [h6n4f1] Gly | G2F+1aGal | 70 | 0.44 | 0.63 | 0.92 | 73 | 1.48 | 1.80 | 2.09 | 69 | 0.29 | 0.35 | 0.45 |
| 7.11 | [h6n4f1] GlyIso | G2F+1aGal[6] | 2 | | 0.49 | | 2 | | 1.62 | | 2 | | 0.30 | |
| 7.12 | [h6n4f1] GlyIso | G2F+1aGal[3] | 2 | | 0.23 | | 2 | | 0.28 | | 2 | | 0.80 | |
| 8 | [h4n5f1] | | 70 | 0.46 | 0.70 | 0.89 | 67 | 0.49 | 0.68 | 0.88 | 67 | 0.89 | 1.03 | 1.14 |
| 8.1 | [h4n5f1] Gly | G1FB | 61 | 0.43 | 0.67 | 0.89 | 57 | 0.49 | 0.67 | 0.87 | 57 | 0.88 | 1.03 | 1.14 |
| 8.2 | [h4n5f1] Gly | G1F+N (tri) | 9 | 0.51 | 0.55 | 0.81 | 9 | 0.46 | 0.51 | 0.83 | 9 | 0.98 | 1.08 | 1.17 |
| 8.3 | [h4n5f1] Gly | G0F+Hex+HexNAc | 1 | | 0.78 | | 1 | | 0.86 | | 1 | | 0.90 | |
| 8.4 | [h4n5f1] Gly | G1F+N | 1 | | 0.89 | | 1 | | 1.01 | | 1 | | 0.89 | |
| 9 | [h5n3f1] | | 69 | 0.78 | 0.88 | 1.17 | 68 | 0.81 | 0.89 | 1.22 | 67 | 0.90 | 0.98 | 1.06 |
| 9.1 | [h5n3f1] Gly | G1F-N+1aGal | 62 | 0.77 | 0.87 | 1.15 | 61 | 0.81 | 0.89 | 1.21 | 60 | 0.91 | 0.99 | 1.04 |
| 9.2 | [h5n3f1] Gly | Man5G0F hybrid | 7 | 0.23 | 0.28 | 0.80 | 5 | 0.22 | 0.83 | 0.88 | 5 | 0.96 | 1.10 | 1.13 |
| 9.3 | [h5n3f1] Gly | Man4G1F | 4 | 0.75 | 0.84 | 1.60 | 4 | 0.79 | 0.97 | 2.34 | 4 | 0.77 | 0.87 | 0.95 |
| 9.31 | [h5n3f1] GlyIso | Man4G1[3]F hybrid | 2 | | 2.32 | | 2 | | 3.62 | | 2 | | 0.72 | |
| 10 | [h4n3f1] | | 68 | 0.24 | 0.48 | 1.11 | 77 | 1.68 | 2.20 | 2.85 | 66 | 0.16 | 0.27 | 0.44 |
| 10.1 | [h4n3f1] Gly | G1F-N | 66 | 0.26 | 0.50 | 1.13 | 76 | 1.67 | 2.19 | 2.77 | 64 | 0.17 | 0.27 | 0.46 |
| 10.11 | [h4n3f1] GlyIso | G1F-N[6] | 8 | 0.19 | 0.22 | 0.72 | 8 | 0.23 | 1.28 | 2.44 | 7 | 0.51 | 0.89 | 1.02 |
| 10.12 | [h4n3f1] GlyIso | G1F-N[3] | 13 | 0.21 | 0.42 | 0.75 | 12 | 1.00 | 1.78 | 2.22 | 11 | 0.12 | 0.26 | 0.38 |
| 10.2 | [h4n3f1] Gly | Man4F+N | 3 | 9.9E-2 | 0.12 | 0.13 | 2 | | 1.50 | | 2 | | 0.83 | |
| 11 | [h3n3] | | 69 | 0.32 | 0.46 | 0.78 | 63 | 0.32 | 0.44 | 0.76 | 61 | 0.97 | 1.08 | 1.17 |
| 11.1 | [h3n3] Gly | G0-N | 69 | 0.29 | 0.46 | 0.72 | 63 | 0.32 | 0.43 | 0.76 | 61 | 0.97 | 1.08 | 1.17 |
| 11.11 | [h3n3] GlyIso | G0-N[6] | 7 | 0.35 | 0.38 | 0.54 | 6 | 0.34 | 0.39 | 0.41 | 6 | 1.00 | 1.01 | 1.18 |
| 11.12 | [h3n3] GlyIso | G0-N[3] | 4 | 0.10 | 0.34 | 0.80 | 4 | 7.3E-2 | 0.27 | 0.54 | 4 | 1.21 | 1.35 | 1.55 |
| 11.2 | [h3n3] Gly | Man3B | 2 | | 0.48 | | 2 | | 0.57 | | 2 | | 0.90 | |
| 12 | [h4n3f1g1] | | 60 | 0.70 | 0.95 | 1.30 | 60 | 0.79 | 1.01 | 1.33 | 59 | 0.90 | 0.97 | 1.06 |
| 12.1 | [h4n3f1g1] Gly | G1FS-N (NeuGc) | 60 | 0.70 | 0.95 | 1.30 | 60 | 0.79 | 1.01 | 1.33 | 59 | 0.90 | 0.97 | 1.06 |
| 13 | [h3n4] | | 63 | 0.16 | 0.28 | 0.84 | 58 | 0.13 | 0.19 | 0.74 | 57 | 1.16 | 1.25 | 1.47 |
| 13.1 | [h3n4] Gly | G0 | 62 | 0.16 | 0.25 | 0.81 | 57 | 0.13 | 0.19 | 0.74 | 56 | 1.16 | 1.25 | 1.47 |
| 13.2 | [h3n4] Gly | G0B-N | 1 | | 0.98 | | 1 | | 0.69 | | 1 | | 1.43 | |

| Measurand | | | Sample A, % | | | | Sample B, % | | | | A/B Ratio | | | |
|---|---|---|---|---|---|---|---|---|---|---|---|---|---|---|
| Index | Composition | Common Name | # | 25% | Median | 75% | # | 25% | Median | 75% | # | 25% | Median | 75% |
| 13.21 | [h3n4] GlyIso | G0B-N[3] | 1 | | 0.26 | | 1 | | 0.19 | | 1 | | 1.37 | |
| 13.22 | [h3n4] GlyIso | G0B-N[6] | 1 | | 0.72 | | 1 | | 0.50 | | 1 | | 1.45 | |
| 14 | [h4n4f1g1] | | 53 | 0.31 | 0.51 | 0.77 | 56 | 0.21 | 0.35 | 0.65 | 52 | 1.04 | 1.34 | 1.58 |
| 14.1 | [h4n4f1g1] Gly | G1FS (NeuGc) | 53 | 0.31 | 0.51 | 0.77 | 56 | 0.21 | 0.35 | 0.65 | 52 | 1.04 | 1.34 | 1.58 |
| 14.11 | [h4n4f1g1] GlyIso | G1FS[6] (NeuGc) | 5 | 0.13 | 0.27 | 0.29 | 5 | 0.14 | 0.27 | 0.28 | 5 | 0.92 | 0.96 | 1.05 |
| 14.12 | [h4n4f1g1] GlyIso | G1FS[3] (NeuGc) | 4 | 0.42 | 0.48 | 0.48 | 4 | 0.32 | 0.46 | 0.64 | 4 | 0.59 | 1.06 | 1.50 |
| 15 | [h6n4f1g1] | | 50 | 0.31 | 0.44 | 0.62 | 49 | 0.37 | 0.46 | 0.72 | 49 | 0.85 | 0.98 | 1.05 |
| 15.1 | [h6n4f1g1] Gly | G2FS+1aGal (NeuGc) | 50 | 0.31 | 0.44 | 0.62 | 49 | 0.37 | 0.46 | 0.72 | 49 | 0.85 | 0.98 | 1.05 |
| 16 | [h5n4f1g1] | | 50 | 0.15 | 0.27 | 0.47 | 50 | 0.29 | 0.45 | 0.72 | 48 | 0.58 | 0.64 | 0.75 |
| 16.1 | [h5n4f1g1] Gly | G2FS (NeuGc) | 50 | 0.15 | 0.27 | 0.47 | 50 | 0.29 | 0.45 | 0.72 | 48 | 0.58 | 0.64 | 0.75 |
| 17 | [h5n5f1] | | 51 | 0.20 | 0.29 | 0.46 | 58 | 0.34 | 0.46 | 0.61 | 48 | 0.46 | 0.55 | 0.93 |
| 17.1 | [h5n5f1] Gly | G2FB | 44 | 0.19 | 0.28 | 0.46 | 51 | 0.34 | 0.47 | 0.61 | 42 | 0.47 | 0.55 | 0.84 |
| 17.2 | [h5n5f1] Gly | G1FB+1aGal | 1 | | 0.46 | | 1 | | 0.42 | | 1 | | 1.09 | |
| 17.3 | [h5n5f1] Gly | G2F+N (tri) | 5 | 0.32 | 0.37 | 0.39 | 7 | 0.17 | 0.24 | 0.46 | 4 | 0.90 | 1.15 | 4.97 |
| 17.31 | [h5n5f1] GlyIso | G(4)1F+N+1aGal | 2 | | 0.29 | | 2 | | 0.31 | | 1 | | 1.02 | |
| 17.32 | [h5n5f1] GlyIso | G(4)2F+N (tri) | 4 | 0.27 | 0.35 | 0.48 | 5 | 0.15 | 0.19 | 0.54 | 3 | 0.75 | 1.29 | 8.64 |
| 17.4 | [h5n5f1] Gly | G0F+2Hex+HexNAc | 2 | | 9.1E-2 | | 2 | | 0.19 | | 2 | | 1.05 | |
| 18 | [h3n5f1] | | 55 | 0.64 | 0.78 | 1.07 | 46 | 0.30 | 0.49 | 1.63 | 45 | 0.69 | 1.71 | 2.22 |
| 18.1 | [h3n5f1] Gly | G0FB | 50 | 0.64 | 0.77 | 1.01 | 41 | 0.30 | 0.48 | 1.36 | 40 | 1.07 | 1.79 | 2.20 |
| 18.2 | [h3n5f1] Gly | G0F+N (tri) | 4 | 0.83 | 1.11 | 1.30 | 4 | 1.96 | 2.44 | 2.46 | 4 | 0.37 | 0.46 | 0.53 |
| 18.3 | [h3n5f1] Gly | ManF+3N | 2 | | 1.06 | | 2 | | 0.44 | | 2 | | 2.37 | |
| 18.31 | [h3n5f1] GlyIso | G0F+N | 1 | | 1.48 | | 1 | | 0.59 | | 1 | | 2.52 | |
| 19 | [h3n2f1] | | 43 | 0.10 | 0.14 | 0.45 | 45 | 0.10 | 0.14 | 0.33 | 39 | 0.95 | 1.00 | 1.06 |
| 19.1 | [h3n2f1] Gly | Man3F | 43 | 0.10 | 0.14 | 0.45 | 45 | 0.10 | 0.14 | 0.33 | 39 | 0.95 | 1.00 | 1.06 |
| 20 | [h4n4] | | 41 | 0.16 | 0.47 | 1.06 | 35 | 0.18 | 0.49 | 1.88 | 35 | 0.63 | 0.89 | 1.35 |
| 20.1 | [h4n4] Gly | G1 | 40 | 0.15 | 0.52 | 1.08 | 34 | 0.18 | 0.58 | 1.98 | 34 | 0.61 | 0.90 | 1.35 |
| 20.11 | [h4n4] GlyIso | G1[6] | 5 | 0.10 | 0.95 | 1.01 | 5 | 0.18 | 1.38 | 1.60 | 5 | 0.55 | 0.60 | 1.00 |
| 20.12 | [h4n4] GlyIso | G1[3] | 7 | 0.13 | 0.25 | 0.53 | 5 | 7.3E-2 | 0.35 | 0.61 | 5 | 0.85 | 0.91 | 1.23 |

| Measurand | | | Sample A, % | | | | Sample B, % | | | | A/B Ratio | | | |
|---|---|---|---|---|---|---|---|---|---|---|---|---|---|---|
| Index | Composition | Common Name | # | 25% | Median | 75% | # | 25% | Median | 75% | # | 25% | Median | 75% |
| 20.2 | [h4n4] Gly | G1B-N | 1 | | 0.16 | | 1 | | 0.22 | | 1 | | 0.74 | |
| 21 | [h5n4] | | 36 | 0.44 | 0.70 | 0.97 | 36 | 0.31 | 0.54 | 1.08 | 35 | 0.75 | 1.05 | 1.20 |
| 21.1 | [h5n4] Gly | G2 | 36 | 0.38 | 0.63 | 0.94 | 36 | 0.31 | 0.53 | 1.01 | 35 | 0.71 | 1.02 | 1.20 |
| 21.2 | [h5n4] Gly | G1+1aGal | 1 | | 0.91 | | 1 | | 0.70 | | 1 | | 1.30 | |
| 22 | [h4n3] | | 26 | 5.6E-2 | 0.11 | 0.38 | 28 | 6.9E-2 | 0.12 | 0.44 | 23 | 0.68 | 1.02 | 1.39 |
| 22.1 | [h4n3] Gly | G1-N | 23 | 5.4E-2 | 9.6E-2 | 0.42 | 25 | 6.7E-2 | 0.12 | 0.47 | 20 | 0.63 | 0.98 | 1.37 |
| 22.2 | [h4n3] Gly | Man4+N | 3 | 0.14 | 0.15 | 0.16 | 3 | 7.7E-2 | 8.0E-2 | 0.10 | 3 | 1.48 | 1.83 | 2.14 |
| 22.21 | [h4n3] GlyIso | Man4+N[3] | 2 | | 0.16 | | 2 | | 7.7E-2 | | 2 | | 2.14 | |
| 23 | [h7n5f1] | | 22 | 6.2E-2 | 0.11 | 0.17 | 23 | 8.0E-2 | 0.11 | 0.16 | 20 | 0.72 | 0.84 | 1.04 |
| 23.1 | [h7n5f1] Gly | G2FB+2aGal | 17 | 5.6E-2 | 0.10 | 0.18 | 17 | 8.0E-2 | 1.0E-1 | 0.14 | 15 | 0.71 | 0.85 | 1.08 |
| 23.2 | [h7n5f1] Gly | G3F+1aGal | 3 | 7.6E-2 | 8.5E-2 | 0.11 | 3 | 8.0E-2 | 8.0E-2 | 0.15 | 3 | 0.72 | 0.83 | 0.95 |
| 23.21 | [h7n5f1] GlyIso | G(4)3F+1aGal | 1 | | 8.5E-2 | | 1 | | 8.0E-2 | | 1 | | 1.06 | |
| 23.3 | [h7n5f1] Gly | G2F+N+2aGal (tri) | 2 | | 0.10 | | 2 | | 0.12 | | 2 | | 0.89 | |
| 23.4 | [h7n5f1] Gly | Man5F+3N+2Gal | 0 | | | | 1 | | 0.17 | | 0 | | | |
| 24 | [h6n3f1] | | 21 | 7.8E-2 | 0.19 | 0.27 | 27 | 0.13 | 0.19 | 0.29 | 19 | 0.40 | 0.70 | 1.00 |
| 24.1 | [h6n3f1] Gly | Man5G1F hybrid | 18 | 6.9E-2 | 0.15 | 0.26 | 24 | 0.12 | 0.18 | 0.34 | 16 | 0.41 | 0.64 | 0.93 |
| 24.2 | [h6n3f1] Gly | Man4G1F+1aGal hybrid | 2 | | 0.23 | | 2 | | 0.21 | | 2 | | 1.13 | |
| 25 | [h6n5f1] | | 18 | 5.0E-2 | 0.11 | 0.20 | 38 | 0.20 | 0.28 | 0.43 | 18 | 0.14 | 0.33 | 0.61 |
| 25.1 | [h6n5f1] Gly | G2FB+1aGal | 12 | 7.2E-2 | 0.14 | 0.18 | 21 | 0.16 | 0.26 | 0.37 | 12 | 0.22 | 0.37 | 0.70 |
| 25.2 | [h6n5f1] Gly | G3F | 6 | 4.5E-2 | 6.0E-2 | 0.19 | 17 | 0.26 | 0.32 | 0.44 | 6 | 0.12 | 0.21 | 0.44 |
| 25.21 | [h6n5f1] GlyIso | G(4)3F | 2 | | 3.5E-2 | | 11 | 0.22 | 0.32 | 0.38 | 2 | | 8.8E-2 | |
| 26 | [h5n3f1g1] | | 20 | 8.9E-2 | 0.12 | 0.21 | 20 | 9.3E-2 | 0.12 | 0.17 | 17 | 0.94 | 1.06 | 1.20 |
| 26.1 | [h5n3f1g1] Gly | Man5G0FS (NeuGc) hybrid | 13 | 9.6E-2 | 0.13 | 0.21 | 11 | 9.1E-2 | 0.14 | 0.17 | 11 | 1.00 | 1.12 | 1.22 |
| 26.2 | [h5n3f1g1] Gly | Man4G1FS (NeuGc) | 4 | 0.11 | 0.12 | 0.13 | 6 | 0.10 | 0.12 | 0.13 | 3 | 1.03 | 1.07 | 1.14 |
| 27 | [h5n4a1] | | 18 | 0.21 | 0.36 | 0.54 | 19 | 0.20 | 0.26 | 1.04 | 16 | 0.69 | 1.24 | 1.51 |
| 27.1 | [h5n4a1] Gly | G2S (NeuAc) | 18 | 0.21 | 0.36 | 0.54 | 19 | 0.20 | 0.26 | 1.04 | 16 | 0.69 | 1.24 | 1.51 |
| 28 | [h6n3] | | 16 | 4.8E-2 | 9.5E-2 | 0.31 | 20 | 0.15 | 0.23 | 0.43 | 16 | 0.22 | 0.50 | 0.87 |
| 28.1 | [h6n3] Gly | Man5G1 hybrid | 16 | 4.8E-2 | 9.5E-2 | 0.31 | 20 | 0.15 | 0.23 | 0.43 | 16 | 0.22 | 0.50 | 0.87 |

| Measurand | | | Sample A, % | | | | Sample B, % | | | | A/B Ratio | | | |
|---|---|---|---|---|---|---|---|---|---|---|---|---|---|---|
| Index | Composition | Common Name | # | 25% | Median | 75% | # | 25% | Median | 75% | # | 25% | Median | 75% |
| 29 | [h5n3] | | 17 | 9.0E-2 | 0.13 | 0.23 | 17 | 3.9E-2 | 8.0E-2 | 9.7E-2 | 15 | 1.17 | 1.71 | 2.48 |
| 29.1 | [h5n3] Gly | G1-N+1aGal | 17 | 9.0E-2 | 0.13 | 0.23 | 17 | 3.9E-2 | 8.0E-2 | 9.7E-2 | 15 | 1.17 | 1.71 | 2.48 |
| 30 | [h6n2] | | 17 | 6.0E-2 | 0.30 | 0.47 | 17 | 4.0E-2 | 0.33 | 0.50 | 15 | 0.89 | 0.98 | 1.12 |
| 30.1 | [h6n2] Gly | Man6 | 17 | 6.0E-2 | 0.30 | 0.47 | 17 | 4.0E-2 | 0.33 | 0.50 | 15 | 0.89 | 0.98 | 1.12 |
| 31 | [h6n4f2] | | 18 | 8.6E-2 | 0.15 | 0.24 | 16 | 9.9E-2 | 0.12 | 0.24 | 15 | 0.82 | 0.98 | 1.04 |
| 31.1 | [h6n4f2] Gly | G2F2+1aGal | 17 | 0.11 | 0.16 | 0.24 | 16 | 9.9E-2 | 0.12 | 0.24 | 15 | 0.82 | 0.98 | 1.04 |
| 31.2 | [h6n4f2] Gly | Man5G1F2B hybrid | 1 | | 2.7E-2 | | 0 | | | | 0 | | | |
| 32 | [h6n3f1g1] | | 15 | 5.6E-2 | 0.10 | 0.19 | 16 | 4.1E-2 | 7.4E-2 | 0.13 | 13 | 0.95 | 1.00 | 1.14 |
| 32.1 | [h6n3f1g1] Gly | Man5G1FS (NeuAc) hybrid | 15 | 5.6E-2 | 0.10 | 0.19 | 16 | 4.1E-2 | 7.4E-2 | 0.13 | 13 | 0.95 | 1.00 | 1.14 |
| 33 | [h7n3f1] | | 14 | 4.6E-2 | 8.5E-2 | 0.13 | 16 | 2.7E-2 | 6.3E-2 | 0.11 | 13 | 1.01 | 1.13 | 1.25 |
| 33.1 | [h7n3f1] Gly | Man5G1F+1aGal hybrid | 14 | 4.6E-2 | 8.5E-2 | 0.13 | 16 | 2.7E-2 | 6.3E-2 | 0.11 | 13 | 1.01 | 1.13 | 1.25 |
| 34 | [h9n5f1] | | 13 | 5.0E-2 | 7.1E-2 | 7.5E-2 | 13 | 4.7E-2 | 5.7E-2 | 6.9E-2 | 13 | 0.96 | 1.06 | 1.10 |
| 34.1 | [h9n5f1] Gly | G3F+3aGal | 6 | 7.4E-2 | 7.6E-2 | 7.9E-2 | 6 | 6.9E-2 | 7.3E-2 | 8.1E-2 | 6 | 1.00 | 1.10 | 1.10 |
| 34.2 | [h9n5f1] Gly | G2FBG+3aGal | 1 | | 5.3E-2 | | 1 | | 5.7E-2 | | 1 | | 0.93 | |
| 35 | [h5n4f1a1] | | 16 | 0.10 | 0.31 | 0.82 | 17 | 8.3E-2 | 0.33 | 1.03 | 12 | 0.48 | 0.98 | 1.08 |
| 35.1 | [h5n4f1a1] Gly | G2FS (NeuAc) | 16 | 0.10 | 0.31 | 0.82 | 17 | 8.3E-2 | 0.33 | 1.03 | 12 | 0.48 | 0.98 | 1.08 |
| 36 | [h4n3f1a1] | | 13 | 0.58 | 0.83 | 1.32 | 13 | 0.24 | 0.59 | 1.12 | 11 | 0.95 | 1.00 | 1.12 |
| 36.1 | [h4n3f1a1] Gly | G1FS-N (NeuAc) | 13 | 0.58 | 0.83 | 1.32 | 13 | 0.24 | 0.59 | 1.12 | 11 | 0.95 | 1.00 | 1.12 |
| 37 | [h3n2] | | 11 | 3.5E-2 | 6.3E-2 | 0.42 | 10 | 3.1E-2 | 5.7E-2 | 0.30 | 10 | 0.90 | 1.02 | 1.29 |
| 37.1 | [h3n2] Gly | Man3 | 11 | 3.5E-2 | 6.3E-2 | 0.42 | 10 | 3.1E-2 | 5.7E-2 | 0.30 | 10 | 0.90 | 1.02 | 1.29 |
| 38 | [h4n4f2] | | 11 | 0.10 | 0.19 | 0.31 | 13 | 7.7E-2 | 0.15 | 0.21 | 10 | 1.27 | 1.45 | 1.53 |
| 38.1 | [h4n4f2] Gly | G1F2 | 11 | 0.10 | 0.19 | 0.31 | 13 | 7.7E-2 | 0.15 | 0.21 | 10 | 1.27 | 1.45 | 1.53 |
| 39 | [h7n3] | | 10 | 7.0E-2 | 7.3E-2 | 0.11 | 10 | 5.4E-2 | 8.8E-2 | 0.14 | 10 | 0.70 | 0.92 | 1.23 |
| 39.1 | [h7n3] Gly | Man5G1+1aGal hybrid | 10 | 7.0E-2 | 7.3E-2 | 0.11 | 10 | 5.4E-2 | 8.8E-2 | 0.14 | 10 | 0.70 | 0.92 | 1.23 |
| 40 | [h4n4f1a1] | | 9 | 0.17 | 0.26 | 0.62 | 10 | 0.20 | 0.41 | 0.81 | 7 | 0.33 | 0.70 | 1.11 |
| 40.1 | [h4n4f1a1] Gly | G1FS (NeuAc) | 9 | 0.17 | 0.26 | 0.62 | 10 | 0.20 | 0.41 | 0.81 | 7 | 0.33 | 0.70 | 1.11 |
| 41 | [h5n4f1a2] | | 7 | 0.38 | 0.43 | 0.83 | 8 | 0.32 | 0.41 | 0.69 | 7 | 0.87 | 0.90 | 1.00 |
| 41.1 | [h5n4f1a2] Gly | G2FS2 (NeuAc) | 7 | 0.38 | 0.43 | 0.83 | 8 | 0.32 | 0.41 | 0.69 | 7 | 0.87 | 0.90 | 1.00 |

| Measurand | | | Sample A, % | | | | Sample B, % | | | | A/B Ratio | | | |
|---|---|---|---|---|---|---|---|---|---|---|---|---|---|---|
| Index | Composition | Common Name | # | 25% | Median | 75% | # | 25% | Median | 75% | # | 25% | Median | 75% |
| 42 | [h2n3f1] | | 6 | 0.31 | 0.58 | 1.13 | 6 | 0.29 | 0.62 | 1.03 | 6 | 0.82 | 1.07 | 1.19 |
| 42.1 | [h2n3f1] Gly | Fragment Man2F+N | 6 | 0.31 | 0.58 | 1.13 | 6 | 0.29 | 0.62 | 1.03 | 6 | 0.82 | 1.07 | 1.19 |
| 43 | [h3n5] | | 6 | 0.33 | 0.42 | 0.60 | 7 | 0.10 | 0.17 | 0.42 | 6 | 1.12 | 1.85 | 3.36 |
| 43.1 | [h3n5] Gly | G0B | 6 | 0.32 | 0.42 | 0.60 | 7 | 0.10 | 0.17 | 0.42 | 6 | 1.12 | 1.85 | 3.36 |
| 43.2 | [h3n5] Gly | G0+N (tri) | 1 | | 1.0E-2 | | 1 | | 1.0E-2 | | 1 | | 1.00 | |
| 44 | [h4n3f2] | | 6 | 8.9E-2 | 0.14 | 0.23 | 7 | 5.6E-2 | 0.12 | 0.19 | 6 | 0.91 | 1.06 | 1.17 |
| 44.1 | [h4n3f2] Gly | G1F2-N | 4 | 0.15 | 0.20 | 0.33 | 4 | 0.16 | 0.19 | 0.32 | 4 | 0.88 | 1.00 | 1.13 |
| 45 | [h4n5f1g1] | | 8 | 3.5E-2 | 5.5E-2 | 7.9E-2 | 8 | 1.3E-2 | 3.1E-2 | 5.5E-2 | 6 | 1.30 | 2.05 | 2.37 |
| 45.1 | [h4n5f1g1] Gly | G1FBS (NeuGc) | 8 | 3.5E-2 | 5.5E-2 | 7.9E-2 | 8 | 1.3E-2 | 3.1E-2 | 5.5E-2 | 6 | 1.30 | 2.05 | 2.37 |
| 46 | [h5n3a1] | | 7 | 0.19 | 0.30 | 0.84 | 7 | 0.14 | 0.25 | 0.94 | 6 | 0.82 | 1.05 | 1.20 |
| 46.1 | [h5n3a1] Gly | Man4G1S hybrid | 3 | 0.22 | 0.30 | 0.65 | 2 | | 0.63 | | 2 | | 1.10 | |
| 46.2 | [h5n3a1] Gly | Fragment G2S-CoreN (NeuAc) | 1 | | 0.18 | | 1 | | 0.24 | | 1 | | 0.76 | |
| 47 | [h5n4f2] | | 7 | 2.7E-2 | 7.8E-2 | 0.42 | 11 | 6.4E-2 | 8.7E-2 | 0.27 | 6 | 0.34 | 0.79 | 1.17 |
| 47.1 | [h5n4f2] Gly | G2F2 | 7 | 2.7E-2 | 7.8E-2 | 0.42 | 10 | 7.7E-2 | 0.12 | 0.28 | 6 | 0.34 | 0.79 | 1.17 |
| 47.2 | [h5n4f2] Gly | Man4G1F2B hybrid | 0 | | | | 1 | | 5.2E-2 | | 0 | | | |
| 48 | [h5n4g2] | | 8 | 0.14 | 0.33 | 0.60 | 7 | 0.17 | 0.40 | 0.83 | 6 | 0.86 | 0.96 | 1.74 |
| 48.1 | [h5n4g2] Gly | G2S2 (NeuGc) | 8 | 0.14 | 0.33 | 0.60 | 7 | 0.17 | 0.40 | 0.83 | 6 | 0.86 | 0.96 | 1.74 |
| 49 | [h6n3g1] | | 7 | 4.0E-2 | 7.3E-2 | 9.2E-2 | 6 | 5.0E-2 | 6.8E-2 | 0.11 | 6 | 0.70 | 0.83 | 0.92 |
| 49.1 | [h6n3g1] Gly | Man5G1S (NeuGc) hybrid | 4 | 7.0E-2 | 9.2E-2 | 0.32 | 3 | 8.7E-2 | 0.13 | 0.13 | 3 | 0.69 | 0.82 | 0.83 |
| 50 | [h6n4] | | 6 | 9.1E-2 | 0.14 | 0.16 | 6 | 0.22 | 0.34 | 0.90 | 6 | 0.30 | 0.33 | 0.70 |
| 50.1 | [h6n4] Gly | G2+1aGal | 5 | 8.0E-2 | 0.12 | 0.16 | 5 | 0.27 | 0.42 | 1.06 | 5 | 0.30 | 0.30 | 0.37 |
| 50.2 | [h6n4] Gly | Fragment Man5+2N+Hex-CoreN hybrid | 1 | | 0.16 | | 1 | | 0.20 | | 1 | | 0.81 | |
| 51 | [h6n4f1a1] | | 7 | 4.6E-2 | 0.23 | 0.31 | 9 | 7.0E-2 | 0.12 | 0.47 | 6 | 0.74 | 0.86 | 1.16 |
| 51.1 | [h6n4f1a1] Gly | G2FS+1aGal (NeuAc) | 6 | 2.8E-2 | 0.16 | 0.25 | 8 | 5.8E-2 | 0.10 | 0.27 | 5 | 0.74 | 0.77 | 0.96 |
| 51.2 | [h6n4f1a1] Gly | Man5G1FBS (NeuAc) hybrid | 1 | | 0.91 | | 1 | | 0.57 | | 1 | | 1.60 | |
| 52 | [h7n2] | | 6 | 6.3E-2 | 0.25 | 0.95 | 7 | 0.25 | 0.55 | 0.87 | 6 | 0.70 | 1.00 | 1.59 |
| 52.1 | [h7n2] Gly | Man7 | 6 | 6.3E-2 | 0.25 | 0.95 | 7 | 0.25 | 0.55 | 0.87 | 6 | 0.70 | 1.00 | 1.59 |
| 53 | [h4n2] | | 5 | 9.3E-2 | 0.24 | 0.32 | 7 | 9.3E-2 | 9.7E-2 | 0.41 | 5 | 1.02 | 1.04 | 1.31 |

| Measurand | | | Sample A, % | | | | Sample B, % | | | | A/B Ratio | | | |
|---|---|---|---|---|---|---|---|---|---|---|---|---|---|---|
| Index | Composition | Common Name | # | 25% | Median | 75% | # | 25% | Median | 75% | # | 25% | Median | 75% |
| 53.1 | [h4n2] Gly | Man4 | 5 | 9.3E-2 | 0.24 | 0.32 | 7 | 9.3E-2 | 9.7E-2 | 0.41 | 5 | 1.02 | 1.04 | 1.31 |
| 53.11 | [h4n2] GlyIso | Man4D2 | 1 | | 0.70 | | 1 | | 0.58 | | 1 | | 1.21 | |
| 54 | [h4n3g1] | | 6 | 2.4E-2 | 4.1E-2 | 9.6E-2 | 5 | 3.3E-2 | 3.7E-2 | 0.13 | 5 | 0.63 | 0.85 | 0.85 |
| 54.1 | [h4n3g1] Gly | G1S-N (NeuGc) | 6 | 2.4E-2 | 4.1E-2 | 9.6E-2 | 5 | 3.3E-2 | 3.7E-2 | 0.13 | 5 | 0.63 | 0.85 | 0.85 |
| 55 | [h5n4f1g2] | | 7 | 3.2E-2 | 4.6E-2 | 0.24 | 5 | 6.3E-2 | 7.4E-2 | 0.79 | 5 | 0.53 | 0.82 | 0.83 |
| 55.1 | [h5n4f1g2] Gly | G2FS2 (NeuGc) | 7 | 3.2E-2 | 4.6E-2 | 0.24 | 5 | 6.3E-2 | 7.4E-2 | 0.79 | 5 | 0.53 | 0.82 | 0.83 |
| 56 | [h8n5f1] | | 5 | 1.3E-2 | 2.0E-2 | 6.6E-2 | 9 | 5.0E-2 | 6.0E-2 | 7.5E-2 | 5 | 0.22 | 0.24 | 0.75 |
| 56.1 | [h8n5f1] Gly | G3F+2aGal | 2 | | 4.0E-2 | | 4 | 6.6E-2 | 7.2E-2 | 7.8E-2 | 2 | | 0.50 | |
| 56.2 | [h8n5f1] Gly | G2FBG+2aGal | 1 | | 4.5E-3 | | 2 | | 4.9E-2 | | 1 | | 0.12 | |
| 57 | [h4n5] | | 4 | 0.30 | 0.37 | 2.87 | 4 | 9.5E-2 | 0.34 | 1.27 | 4 | 0.85 | 1.97 | 4.10 |
| 57.1 | [h4n5] Gly | G1B | 4 | 0.30 | 0.37 | 2.87 | 4 | 9.5E-2 | 0.34 | 1.27 | 4 | 0.85 | 1.97 | 4.10 |
| 57.11 | [h4n5] GlyIso | G1B[6] | 1 | | 4.0E-2 | | 1 | | 5.0E-2 | | 1 | | 0.80 | |
| 57.12 | [h4n5] GlyIso | G1B[3] | 1 | | 6.0E-2 | | 1 | | 6.0E-2 | | 1 | | 1.00 | |
| 58 | [h5n3f2] | | 5 | 2.4E-2 | 6.5E-2 | 0.66 | 5 | 2.8E-2 | 0.42 | 0.79 | 4 | 0.75 | 0.84 | 0.87 |
| 58.1 | [h5n3f2] Gly | G1F2-N+1aGal | 5 | 2.4E-2 | 6.5E-2 | 0.66 | 5 | 2.8E-2 | 0.42 | 0.79 | 4 | 0.75 | 0.84 | 0.87 |
| 59 | [h5n4a2] | | 5 | 0.20 | 0.27 | 0.74 | 5 | 0.13 | 0.13 | 0.20 | 4 | 1.26 | 1.44 | 2.50 |
| 59.1 | [h5n4a2] Gly | G2S2 (NeuAc) | 5 | 0.20 | 0.27 | 0.74 | 5 | 0.13 | 0.13 | 0.20 | 4 | 1.26 | 1.44 | 2.50 |
| 60 | [h5n4g1] | | 7 | 0.10 | 0.14 | 0.16 | 4 | 0.72 | 0.93 | 1.13 | 4 | 0.10 | 0.13 | 0.45 |
| 60.1 | [h5n4g1] Gly | G2S (NeuGc) | 7 | 0.10 | 0.14 | 0.16 | 4 | 0.72 | 0.93 | 1.13 | 4 | 0.10 | 0.13 | 0.45 |
| 61 | [h5n5f2] | | 4 | 1.0E-2 | 4.5E-2 | 0.25 | 4 | 1.1E-2 | 2.3E-2 | 0.21 | 4 | 1.00 | 1.01 | 1.32 |
| 61.1 | [h5n5f2] Gly | G2F2B | 1 | | 1.1E-2 | | 1 | | 1.1E-2 | | 1 | | 0.98 | |
| 61.2 | [h5n5f2] Gly | G2F2+N (tri) | 1 | | 1.0E-2 | | 1 | | 1.0E-2 | | 1 | | 1.00 | |
| 62 | [h7n4a1] | | 4 | 0.22 | 0.29 | 0.51 | 4 | 0.20 | 0.27 | 0.47 | 4 | 1.08 | 1.10 | 1.12 |
| 62.1 | [h7n4a1] Gly | G2S+2aGal (NeuAc) | 1 | | 0.33 | | 1 | | 0.32 | | 1 | | 1.05 | |
| 62.2 | [h7n4a1] Gly | Man5G2S (NeuAc) hybrid | 1 | | 1.05 | | 1 | | 0.95 | | 1 | | 1.11 | |
| 62.3 | [h7n4a1] Gly | G2S(6)+1aGal+Gal (NeuAc) | 1 | | 0.13 | | 1 | | 0.11 | | 1 | | 1.18 | |
| 63 | [h8n2] | | 4 | 0.13 | 0.19 | 0.44 | 4 | 0.13 | 0.27 | 0.50 | 4 | 0.77 | 1.11 | 1.34 |
| 63.1 | [h8n2] Gly | Man8 | 4 | 0.13 | 0.19 | 0.44 | 4 | 0.13 | 0.27 | 0.50 | 4 | 0.77 | 1.11 | 1.34 |

| Measurand | | | Sample A, % | | | | Sample B, % | | | | A/B Ratio | | | |
|---|---|---|---|---|---|---|---|---|---|---|---|---|---|---|
| Index | Composition | Common Name | # | 25% | Median | 75% | # | 25% | Median | 75% | # | 25% | Median | 75% |
| 64 | [h4n2f1] | | 3 | 0.16 | 0.18 | 4.92 | 3 | 0.92 | 1.73 | 7.79 | 3 | 0.40 | 0.70 | 0.95 |
| 64.1 | [h4n2f1] Gly | Man4F | 3 | 0.16 | 0.18 | 4.92 | 3 | 0.92 | 1.73 | 7.79 | 3 | 0.40 | 0.70 | 0.95 |
| 64.11 | [h4n2f1] GlyIso | Man4D2F | 1 | | 0.18 | | 1 | | 1.73 | | 1 | | 0.10 | |
| 65 | [h4n4a1] | | 3 | 0.37 | 0.50 | 0.70 | 3 | 0.12 | 0.24 | 0.47 | 3 | 1.16 | 1.29 | 25.65 |
| 65.1 | [h4n4a1] Gly | G1S (NeuAc) | 3 | 0.37 | 0.50 | 0.70 | 3 | 0.12 | 0.24 | 0.47 | 3 | 1.16 | 1.29 | 25.65 |
| 66 | [h5n3g1] | | 3 | 1.8E-2 | 2.0E-2 | 6.3E-2 | 3 | 2.1E-2 | 4.0E-2 | 7.5E-2 | 3 | 0.73 | 0.97 | 5.77 |
| 66.1 | [h5n3g1] Gly | Man4G1S1 (NeuGc) | 1 | | 0.11 | | 1 | | 0.11 | | 1 | | 0.97 | |
| 67 | [h5n5] | | 4 | 0.27 | 0.48 | 0.99 | 3 | 1.14 | 1.36 | 1.46 | 3 | 0.43 | 0.74 | 1.09 |
| 67.1 | [h5n5] Gly | G2B | 3 | 0.44 | 0.67 | 1.32 | 3 | 1.14 | 1.36 | 1.46 | 3 | 0.43 | 0.74 | 1.09 |
| 67.2 | [h5n5] Gly | G1+N+1aGal (tri) | 1 | | 0.29 | | 0 | | | | 0 | | | |
| 68 | [h6n5f1g1] | | 4 | 9.1E-3 | 2.1E-2 | 3.7E-2 | 3 | 1.1E-2 | 1.3E-2 | 2.9E-2 | 3 | 0.82 | 1.17 | 1.20 |
| 68.1 | [h6n5f1g1] Gly | G2FBS+1aGal (NeuGc) | 1 | | 1.0E-2 | | 1 | | 8.2E-3 | | 1 | | 1.22 | |
| 68.2 | [h6n5f1g1] Gly | G3FS (NeuGc) | 1 | | 3.2E-2 | | 0 | | | | 0 | | | |
| 69 | [h8n5f1g1] | | 3 | 2.6E-2 | 3.7E-2 | 4.0E-2 | 3 | 2.5E-2 | 3.3E-2 | 3.8E-2 | 3 | 0.92 | 1.00 | 1.07 |
| 69.1 | [h8n5f1g1] Gly | G3FS+2aGal (NeuGc) | 2 | | 4.0E-2 | | 2 | | 3.8E-2 | | 2 | | 1.07 | |
| 69.2 | [h8n5f1g1] Gly | G2FBGS+3aGal (NeuGc) | 1 | | 1.6E-2 | | 1 | | 1.8E-2 | | 1 | | 0.85 | |
| 70 | [h4n5f1a1] | | 2 | | 0.38 | | 4 | 6.3E-2 | 0.49 | 1.48 | 2 | | 1.41 | |
| 70.1 | [h4n5f1a1] Gly | G1FBS | 2 | | 0.38 | | 4 | 6.3E-2 | 0.49 | 1.48 | 2 | | 1.41 | |
| 71 | [h5n3f1a1] | | 3 | 0.29 | 0.50 | 0.66 | 2 | | 0.89 | | 2 | | 0.75 | |
| 71.1 | [h5n3f1a1] Gly | Man4G1[3]FS (NeuAc) hybrid | 2 | | 0.45 | | 1 | | 0.83 | | 1 | | 0.97 | |
| 71.2 | [h5n3f1a1] Gly | G1FS-N+1aGal (NeuAc) | 1 | | 0.50 | | 1 | | 0.95 | | 1 | | 0.53 | |
| 72 | [h6n3a1] | | 2 | | 4.3E-2 | | 3 | 1.4E-2 | 1.8E-2 | 4.0E-2 | 2 | | 1.09 | |
| 73 | [h6n4a1] | | 3 | 9.3E-2 | 0.12 | 0.15 | 3 | 0.18 | 0.22 | 0.26 | 2 | | 0.71 | |
| 73.1 | [h6n4a1] Gly | G2S+1aGal (NeuAc) | 2 | | 0.13 | | 1 | | 0.31 | | 1 | | 0.60 | |
| 74 | [h6n5] | | 2 | | 0.99 | | 2 | | 1.55 | | 2 | | 0.75 | |
| 74.1 | [h6n5] Gly | G3 | 2 | | 0.99 | | 2 | | 1.55 | | 2 | | 0.75 | |
| 74.11 | [h6n5] GlyIso | G(4)3 | 1 | | 1.08 | | 1 | | 2.20 | | 1 | | 0.49 | |
| 75 | [h6n5a1] | | 2 | | 7.1E-3 | | 2 | | 1.2E-2 | | 2 | | 1.05 | |

| Measurand | | | Sample A, % | | | | Sample B, % | | | | A/B Ratio | | | |
|---|---|---|---|---|---|---|---|---|---|---|---|---|---|---|
| Index | Composition | Common Name | # | 25% | Median | 75% | # | 25% | Median | 75% | # | 25% | Median | 75% |
| 76 | [h8n5f2] | | 2 | | 6.2E-3 | | 2 | | 1.7E-2 | | 2 | | 0.49 | |
| 77 | [h9n2] | | 2 | | 7.3E-2 | | 4 | 4.4E-2 | 6.7E-2 | 9.1E-2 | 2 | | 1.09 | |
| 77.1 | [h9n2] Gly | Man9 | 2 | | 7.3E-2 | | 4 | 4.4E-2 | 6.7E-2 | 9.1E-2 | 2 | | 1.09 | |
| 78 | [h3n3g1] | | 1 | | 9.8E-4 | | 1 | | 1.0E-3 | | 1 | | 0.95 | |
| 79 | [h3n4f1a1] | | 1 | | 2.17 | | 1 | | 2.64 | | 1 | | 0.82 | |
| 80 | [h3n4f1S] | | 1 | | 6.6E-2 | | 1 | | 7.6E-2 | | 1 | | 0.87 | |
| 80.1 | [h3n4f1S] Gly | G0F-N+GalNAc(4-Sul) | 1 | | 6.6E-2 | | 1 | | 7.6E-2 | | 1 | | 0.87 | |
| 81 | [h3n4f2] | | 1 | | 0.18 | | 1 | | 0.14 | | 1 | | 1.37 | |
| 81.1 | [h3n4f2] Gly | G0F2-N[3]+GalNAc | 1 | | 0.18 | | 1 | | 0.14 | | 1 | | 1.37 | |
| 82 | [h3n5f2] | | 1 | | 2.18 | | 1 | | 0.22 | | 1 | | 10.08 | |
| 83 | [h3n7f1] | | 1 | | 0.50 | | 1 | | 0.47 | | 1 | | 1.06 | |
| 83.1 | [h3n7f1] Gly | G0FB+2N (quad) | 1 | | 0.50 | | 1 | | 0.47 | | 1 | | 1.06 | |
| 84 | [h4n3a1] | | 1 | | 6.0E-2 | | 2 | | 16.12 | | 1 | | 0.23 | |
| 84.1 | [h4n3a1] Gly | G1S-N (NeuAc) | 1 | | 6.0E-2 | | 1 | | 0.26 | | 1 | | 0.23 | |
| 85 | [h4n4f1g2] | | 1 | | 2.0E-2 | | 1 | | 2.0E-2 | | 1 | | 1.00 | |
| 85.1 | [h4n4f1g2] Gly | G1FS2 (NeuGc) | 1 | | 2.0E-2 | | 1 | | 2.0E-2 | | 1 | | 1.00 | |
| 86 | [h4n4f1S] | | 1 | | 0.13 | | 1 | | 8.0E-2 | | 1 | | 1.68 | |
| 86.1 | [h4n4f1S] Gly | Man4F+N+GalNAc(4Sul) hybrid | 1 | | 0.13 | | 1 | | 8.0E-2 | | 1 | | 1.68 | |
| 87 | [h4n5a1] | | 1 | | 0.57 | | 1 | | 0.18 | | 1 | | 3.17 | |
| 87.1 | [h4n5a1] Gly | G1S+N (NeuAc) (tri) | 1 | | 0.57 | | 1 | | 0.18 | | 1 | | 3.17 | |
| 88 | [h5n2f1] | | 1 | | 0.31 | | 1 | | 2.28 | | 1 | | 0.14 | |
| 88.1 | [h5n2f1] Gly | Man5F | 1 | | 0.31 | | 1 | | 2.28 | | 1 | | 0.14 | |
| 89 | [h5n5f1a1] | | 1 | | 0.68 | | 1 | | 0.74 | | 1 | | 0.92 | |
| 89.1 | [h5n5f1a1] Gly | G2FS+N (NeuAc) (tri) | 1 | | 0.68 | | 1 | | 0.74 | | 1 | | 0.92 | |
| 90 | [h5n5f1a2] | | 1 | | 0.38 | | 1 | | 0.74 | | 1 | | 0.51 | |
| 90.1 | [h5n5f1a2] Gly | G2FS2+N (NeuAc) (tri) | 1 | | 0.38 | | 1 | | 0.74 | | 1 | | 0.51 | |
| 91 | [h5n5f1g1] | | 1 | | 4.6E-2 | | 1 | | 4.5E-2 | | 1 | | 1.03 | |
| 92 | [h6n2f1] | | 1 | | 4.8E-2 | | 1 | | 0.12 | | 1 | | 0.39 | |

| Measurand | | | Sample A, % | | | | Sample B, % | | | | A/B Ratio | | | |
|---|---|---|---|---|---|---|---|---|---|---|---|---|---|---|
| Index | Composition | Common Name | # | 25% | Median | 75% | # | 25% | Median | 75% | # | 25% | Median | 75% |
| 92.1 | [h6n2f1] Gly | Man6F | 1 | | 4.8E-2 | | 1 | | 0.12 | | 1 | | 0.39 | |
| 93 | [h6n3f2] | | 1 | | 1.9E-2 | | 1 | | 2.1E-2 | | 1 | | 0.89 | |
| 94 | [h6n4g1] | | 1 | | 0.36 | | 1 | | 0.46 | | 1 | | 0.78 | |
| 95 | [h6n5f1g2] | | 1 | | 3.7E-2 | | 1 | | 6.0E-2 | | 1 | | 0.61 | |
| 95.1 | [h6n5f1g2] Gly | G3FS2(NeuGc) | 1 | | 3.7E-2 | | 1 | | 6.0E-2 | | 1 | | 0.61 | |
| 96 | [h6n7f4a3] | | 1 | | 5.7E-2 | | 1 | | 3.0E-2 | | 1 | | 1.89 | |
| 97 | [h6n7f5a2] | | 1 | | 4.8E-2 | | 1 | | 6.0E-2 | | 1 | | 0.80 | |
| 98 | [h7n3f2] | | 1 | | 3.2E-2 | | 1 | | 8.5E-2 | | 1 | | 0.38 | |
| 99 | [h7n4] | | 2 | | 9.0E-2 | | 3 | 7.2E-2 | 0.10 | 0.23 | 1 | | 0.29 | |
| 99.1 | [h7n4] Gly | G2+2aGal | 2 | | 9.0E-2 | | 3 | 7.2E-2 | 0.10 | 0.23 | 1 | | 0.29 | |
| 100 | [h7n5f1g2] | | 1 | | 6.1E-3 | | 1 | | 6.3E-3 | | 1 | | 0.97 | |
| 100.1 | [h7n5f1g2] Gly | G2FBGS2+1aGal (NeuGc) | 1 | | 6.1E-3 | | 1 | | 6.3E-3 | | 1 | | 0.97 | |
| 101 | [h7n5f2] | | 1 | | 1.9E-2 | | 1 | | 3.3E-2 | | 1 | | 0.58 | |
| 102 | [h7n6f2a1] | | 1 | | 0.13 | | 1 | | 0.10 | | 1 | | 1.24 | |
| 102.1 | [h7n6f2a1] Gly | G4F2S (NeuAc) | 1 | | 0.13 | | 1 | | 0.10 | | 1 | | 1.24 | |
| 103 | [h7n8f3a4] | | 1 | | 1.8E-2 | | 1 | | 3.2E-2 | | 1 | | 0.56 | |
| 104 | [h7n9f1a4] | | 1 | | 1.5E-2 | | 1 | | 3.6E-2 | | 1 | | 0.43 | |
| 105 | [h8n10f5a3] | | 1 | | 6.5E-3 | | 1 | | 7.1E-3 | | 1 | | 0.92 | |
| 106 | [h8n8f3a4] | | 1 | | 5.0E-3 | | 1 | | 2.2E-2 | | 1 | | 0.23 | |
| 107 | [n1f1] | | 1 | | 4.9E-3 | | 1 | | 6.2E-3 | | 1 | | 0.79 | |
| 108 | [n2f1] | | 2 | | 9.6E-2 | | 1 | | 0.13 | | 1 | | 1.16 | |
| 108.1 | [n2f1] Gly | Fragment FN2 | 2 | | 9.6E-2 | | 1 | | 0.13 | | 1 | | 1.16 | |
| 109 | [h3n3f2] | | 0 | | | | 1 | | 0.20 | | 0 | | | |
| 109.1 | [h3n3f2] Gly | G0F2-N[3] | 0 | | | | 1 | | 0.20 | | 0 | | | |
| 110 | [h6n2g1] | | * | | | | * | | | | | | | |
| 110.1 | [h6n2g1] Gly | Man5+Gal+S (NeuGc) hybrid | * | | | | * | | | | | | | |
| 111 | [h6n3f1a1] | | 0 | | | | 1 | | 0.61 | | 0 | | | |
| 111.1 | [h6n3f1a1] Gly | Man5G1FS (NeuAc) hybrid | 0 | | | | 1 | | 0.61 | | 0 | | | |

| Measurand | | | Sample A, % | | | | Sample B, % | | | | A/B Ratio | | | |
|---|---|---|---|---|---|---|---|---|---|---|---|---|---|---|
| Index | Composition | Common Name | # | 25% | Median | 75% | # | 25% | Median | 75% | # | 25% | Median | 75% |
| 112 | [h6n3f2a1] | | 0 | | | | 1 | | 2.6E-2 | | 0 | | | |
| 112.1 | [h6n3f2a1] Gly | Man5G1F2S (NeuAc) hybrid | 0 | | | | 1 | | 2.6E-2 | | 0 | | | |
| 113 | [h7n5f1g1] | | 0 | | | | 2 | | 1.8E-2 | | 0 | | | |
| 113.1 | [h7n5f1g1] Gly | G2FBGS+1aGal (NeuGc) | 0 | | | | 1 | | 6.7E-3 | | 0 | | | |
| 114 | [h7n6f1a1g3] | | 0 | | | | 1 | | 5.48 | | 0 | | | |
| 114.1 | [h7n6f1a1g3] Gly | G4FS4 (3NeuGc) (1NeuAc) | 0 | | | | 1 | | 5.48 | | 0 | | | |
| 115 | [h7n8f5a3] | | 1 | | 2.4E-2 | | 0 | | | | 0 | | | |
| 116 | [h7n8f6a3] | | 1 | | 1.2E-2 | | 0 | | | | 0 | | | |
| | [h3n3f1]/[h3n4f1] | G0F-N/G0F | 1 | | 2.80 | | 1 | | 1.07 | | 1 | | 2.63 | |
| | [h3n4]/[h3n4f1] | G0/G1F | 1 | | 1.30 | | 1 | | 0.83 | | 1 | | 1.56 | |
| | [h3n4f1]/[h4n4f1] | G0F/G1F | 1 | | 30.57 | | 1 | | 37.13 | | 1 | | 0.82 | |
| | [h4n4f1]/[h5n4f1] | G1F/G2F | 1 | | 1.70 | | 1 | | 9.37 | | 1 | | 0.18 | |
| | [h5n4f1]/[h6n4f1] | G2F/G2F+1aGal | 0 | | | | 1 | | 0.50 | | 0 | | | |
| | [h5n5f1g1]/[h6n5a1] | | 1 | | 0.12 | | 1 | | 8.0E-2 | | 1 | | 1.46 | |
| | [h6n5a3]/[h5n5f1a2g1]/[h4n5f2a1g2]/[h3n5f3g3] | | 1 | | 5.3E-2 | | 0 | | | | 0 | | | |
| | [h6n5f1a3]/[h5n5f2a2g1]/[h4n5f3a1g2]/[h3n5f4g3] | | 1 | | 2.6E-2 | | 0 | | | | 0 | | | |
| | [Unknown] | | 26 | 0.99 | 1.68 | 5.24 | 25 | 0.77 | 2.60 | 7.05 | 25 | 0.65 | 0.95 | 1.14 |

* Identified but not quantified

**Legend for Supplementary Table 3**.

| Headings and Subheadings | Definition |
|---|---|
| Measurand | The glycan (or identified combination of glycans) for which measurement results were reported. |
| Index: | Decimal index of identified glycan compositions assigned by decreasing number of NISTmAb results.<br>Integers denote unique compositions, tenths glycoforms, and hundredths isomers. |
| Composition: | Composition of the glycan in the De Leoz-Stein notation (see **Table of Identified Glycans** for details.)<br>The result listed for a given composition is the sum of the reported results for all glycoforms of that composition,<br>where the glycoforms are indicated by "[composition] Gly".<br>The result listed for a given glycoform is the sum of the reported results for all isomers of that glycoform<br>where the isomers are indicated by "[composition] GlyIso". |
| Common Name: | When available, a common name of glycoforms and isomers. See **Table of Identified Glycans** for Oxford names. |
| Sample A, % | Summary statistics for results reported for sample A, a modified version of the NISTmAb material |
| #: | The number of participants reporting this measurand in this sample. |
| 25%: | The 25th percentile (1st quartile) of the distribution of the reported results. |
| Median: | The consensus median (50th percentile or 2nd quartile) of the distribution of the reported results. |
| 75%: | The75th percentile (3rd quartile) of the distribution of the reported results. |
| Sample B, % | Summary statistics for results reported for sample B, the NISTmAb material. |
| #: | The number of participants reporting this measurand in this sample. |
| 25%: | The 25th percentile (1st quartile) of the distribution of the reported results. |
| Median: | The consensus median (50th percentile or 2nd quartile) of the distribution of the reported results. |
| 75%: | The75th percentile (3rd quartile) of the distribution of the reported results. |
| A/B Ratio | Summary statistics for the ratio A/B when results were reported for both samples A and B. |
| #: | The number of A/B ratios. |
| 25%: | The 25th percentile (1st quartile) of the distribution of the calculated ratios. |
| Median: | The consensus median (50th percentile or 2nd quartile) of the distribution of the calculated ratios. |
| 75%: | The75th percentile (3rd quartile) of the distribution of the calculated ratios. |

**Supplementary Table 4.** Reported advantages and limitations of select methods as described by the participants.

Method codes: A = analyte, D = derivatization, S = separation, T = Analytical Technique, I = identification, Q = Quantification

| Method | Strength | Uncertainty |
|---|---|---|
| A: glycan<br>D: 2AB<br>T: LC-FD<br>S: HILIC<br>I: RT (GU) with sialic acid derivatization<br>Q: PA | Isomer Differentiation<br>• G1F(1,3) and G1F(1,6)<br>Sialic acid linkage<br>• NeuGc is a2,6 linked in G1FS[3] and G1FS[6] NeuGc | Same GU values<br>• G1F(1,6) and G1-N+1aGal(1,3)<br>• G2+1aGal(1,3) and G1FS-N (NeuGc) |
| A: glycan<br>D: 2AB<br>T: LC-FD<br>S: HILIC<br>I: RT (standard)<br>Q: PA | | Same retention times (co-elution)<br>• G2 and Man6<br>Peaks absent in standard could not be identified |
| A: glycan<br>D: ethyl esterification<br>T: MALDI-MS<br>S: none<br>I: MS mass with sialic acid derivatization<br>Q: summation of isotope peaks | Sialic acid linkage<br>• NeuGc is a2,6 in G1FS-N, G1FS, G2FS, G2FS+1aGal (NeuGc) | Same *m/z*<br>• G1F(1,3) and G1F(1,6)<br>• G2F and G1F+1aGal<br>• FA3G0 and FA2B<br>• G1F-N+1aGal and Man5G0F hybrid<br>• FA3G1 and FA2BG1<br>• FA3G2 or FA2BG2 |
| A: glycan<br>D: glycosylamine<br>T: LC-FD<br>S: HILIC<br>I: RT (standard)<br>Q: PA | | Same retention times (co-elution)<br>• G1F-N, G1(1,6) and Man 5<br>• G2F and G1FS<br>Uncertainty<br>• G1F(1,6) and G1F(1,3) |
| A: protein fragment<br>D: none<br>T: LC-MS<br>S: SEC<br>I: MS mass<br>Q: MS intensity | | Same nominal masses (close to each other but different compositions)<br>• G2FS + 1aGal (NeuGc) [h6n4f1g1] and G2S2 (NeuGc) [h5n4g2]<br>• G1F2-N+1aGal [h5n3f2] and G1FS-N (NeuGc) [h4n3f1g1]<br>• G2F2 [h5n4f2], G1FS (NeuGc) [h4n4f1g1], and G2S (NeuAc) [h5n4a1]<br>• G2F+1aGal [h6n4f1] and G2S [h5n4g1]<br>• G2F2+1aGal [h6n4f2] and G2FS (NeuGc) [h5n4f1g1] |

| Method | Strength | Uncertainty |
|---|---|---|
| A: glycan<br>D: reduction<br>T: LC-MS (QTOF)<br>S: PGC<br>I: MS mass<br>Q: PA, confirmation with MS/MS | Differentiation of isobaric species<br>• h5n4a1 and h4n4f1g1 have mass 1931.688 but by MS/MS confirmation, it was found that it only contained a NeuGc residue | |
| A: glycopeptide<br>D: none<br>S: RP<br>T: LC-MS<br>I: MS/MS<br>Q: MS intensity | Confirmation of glycans by high mass accuracy (< 3 ppm) and fragmentation patterns<br>• Man5, GFS-N<br>Detection of unglycosylated form by high mass accuracy (< 3 ppm) | Isomer differentiation<br>• [h6n5f1] G3F or bisecting G2FB+1aGal. Assignment based on other triantennary structures identified<br>• [h7n5f1] assignment to G3F+1aGal based on other triantennary structures identified<br>• Isolated case of G1FS-N (NeuGc) [h4n3f1g1]: glycopeptide abundance was higher than subunit abundance based on comparison with quantitative data from subunit analysis |
| A: fragment<br>D: none<br>S: C4<br>T: LC-MS<br>I: MS mass<br>Q: MS intensity | Detection of unglycosylated form | Same masses (delta mass ranged from 0.04 Da to 2 Da) |
| A: glycopeptide<br>D: none<br>S: RP<br>T: LC-MS<br>I: MS mass<br>Q: summation of isotope peaks | | Interferences |
| A: intact<br>D: none<br>S: RP<br>T: LC-MS<br>I: MS mass<br>Q: MS intensity | Glycan ratios in samples such as G0F/G1F | |
| A: glycopeptide<br>D: none<br>S: RP<br>T: LC-MS<br>I: MS mass<br>Q: PA | Purity of samples | |

| Method | Strength | Uncertainty |
|---|---|---|
| A: glycan<br>D: APTS<br>S: CE<br>T: CE-LIF<br>I: MTs, exo<br>Q: PH | Isomer differentiation<br>• G1F+1aGal [h5n4f1], G1F-N+1aGal [h5n3f1], G2F+1aGal [h6n4f1], G1FS-N (NeuGc), [h4n3f1g1], G1FS (NeuGc) [h4n4f1g1], G2FS (NeuGc) [h5n4f1g1] | |
